# Two new pterocarpans and a new pyrone derivative with cytotoxic activities from *Ptycholobium contortum* (N.E.Br.) Brummitt (Leguminosae): revised NMR assignment of mundulea lactone

**DOI:** 10.1186/s13065-016-0204-x

**Published:** 2016-10-05

**Authors:** Dominique Ngnintedo, Ghislain W. Fotso, Victor Kuete, Frederic Nana, Louis P. Sandjo, Oğuzhan Karaosmanoğlu, Hülya Sivas, Felix Keumedjio, Gilbert Kirsch, Bonaventure T. Ngadjui, Kerstin Andrae-Marobela

**Affiliations:** 1 Department of Organic Chemistry, Faculty of Science, University of Yaoundé I, Yaoundé, Cameroon; 2 Department of Biochemistry, Faculty of Science, University of Dschang, Dschang, Cameroon; 3 Department of Biology, Science Faculty, Anadolu University, Eskişehir, Turkey; 4 Molecular Engineering Laboratory and Formerly Pharmacological Biochemistry, UMR-SRSMC 7565, University of Lorraine, 1 Boulevard Arago, Metz Technopole, 57070 Nancy, France; 5 Department of Pharmaceutical Sciences, Universidade Federal de Santa Catarina, Campus Universitário, Trindade, Florianópolis, SC 88040–900 Brazil; 6 Department of Biology, Kamil Özdağ Science Faculty, Karamanoğlu Mehmetbey University, Karaman, Turkey; 7 Department of Pharmacognosy and Pharmaceutical Sciences, Faculty of Medicine and Biomedical Science, University of Yaoundé I, Yaoundé, Cameroon; 8 Department of Biological Sciences, Faculty of Science, University of Botswana, Block 235, Private Bag, 0022 Gaborone, Botswana

**Keywords:** Cytotoxic activities, *Ptycholobium contortum*, Ptycholopyrone A, Seputhecarpan C, Seputhecarpan D

## Abstract

**Background:**

*Ptycholobium* is a genus related to *Tephrosia* which comprises only three species. Compared to *Tephrosia*, which has been phytochemically and pharmacologically studied, *Ptycholobium* species have only few or no reports on their chemical constituents. Moreover, no studies on the cytotoxic activities of its secondary metabolites have been previously documented.

**Results:**

From the non polar fractions of the roots bark of *Ptycholobium contortum* (syn *Tephrosia contorta*), two new pterocarpans: seputhecarpan C **1** and seputhecarpan D **2** and a new pyrone derivative, ptycholopyrone A **3** were isolated. Alongside, five known compounds identified as 3-α,α-dimethylallyl-4-methoxy-6-styryl-α-pyrone or mundulea lactone **4**, glyasperin F **5**, seputhecarpan A **6**, seputheisoflavone **7** and 5-*O*-methyl-myo-inositol or sequoyitol **8** were also obtained. Their structures were established by the mean means of spectroscopic data in conjunction to those reported in literature. The NMR assignment of the major compound mundulea lactone **4** is revised in this paper. In addition, the cytotoxicity of the isolated metabolites was evaluated on two lung cancer cell lines A549 and SPC212. **8** was not active while compounds **1, 2, 4–7** displayed antiproliferative effects against the two carcinoma cell lines with IC_50_ values below 75 µM. IC_50_ values below 10 µM were obtained for **4, 6** and **7** on SPC212 cells.

**Conclusion:**

Based on the obtained results, *Ptycholobium contortum* turns to be a rich source of phenolic metabolites among them some bearing prenyl moieties. This study reports for the first time the isolation of pyrone derivatives **3** and **4** from *Ptycholobium* genus. The cytotoxicity observed for the isolate is also reported for the first time and shows that **4, 6** and **7** could be chemically explored in order to develop a hit candidate against lung cancer.

**Electronic supplementary material:**

The online version of this article (doi:10.1186/s13065-016-0204-x) contains supplementary material, which is available to authorized users.

**Graphical abstract:**
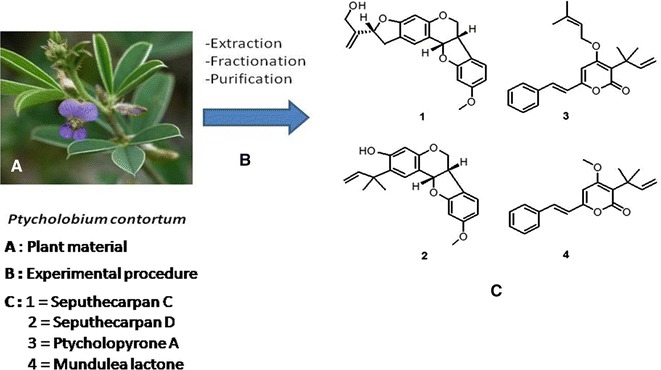
Two new pterocarpans and a new pyrone derivative with cytotoxic activities from ptycholobium contortum (N.E.Br.) Brummitt (Leguminosae): revised NMR assignment of mundulea lactone.

## Background

There is a considerable burden due to lung cancer which is the most common cause of death from the cancer diseases worldwide. Approximately 20 % (1.59 million deaths, 19.4 % of the total) of cancer death are victims of lung cancer [1]. This estimation is continuously constant since several decades and 1.8 million new cases were diagnosed in 2012 (12.9 % of the total, 58 % of which occurred in the less developed regions). The disease remains also prominent in men (1.2 million, 16.7 % of the total) with the highest estimated age-standardized incidence rates in Central and Eastern Europe (0.054 %) and Eastern Asia (0.050 %) [1]. The use of medicinal plants as an alternative or complementary solution remains a partial healthcare solution since the plant kingdom represents one of the sources of hit compounds and drugs candidates against cancer. Chemical constituents of *Tephrosia* species (a related genus of *Ptycholobium*) and their biological benefit (cytotoxic activities) are well known [2]. Recently, we reported on two pterocarpans and one isoflavanone together with their antimicrobial, α-glucosidase and antioxidant properties from the polar fractions of the root bark of *P. contortum.* This work is up to date the only on this genus [3]. This work is the only report on this genus up to date [3]. We herein report the isolation and the structure elucidation of two new pterocarpans, a new pyrone derivative along with the cytotoxic activities of the isolated compounds.

## Results and discussion

The crude extract of *P. contortum* roots was partitioned with *n*-hexane, chloroform, ethyl acetate and *n*-butanol. Purification of the hexane and ethyl acetate fractions by successive column chromatography afforded eight compounds among them three new (**1**–**3**).

**Fig. 1:**
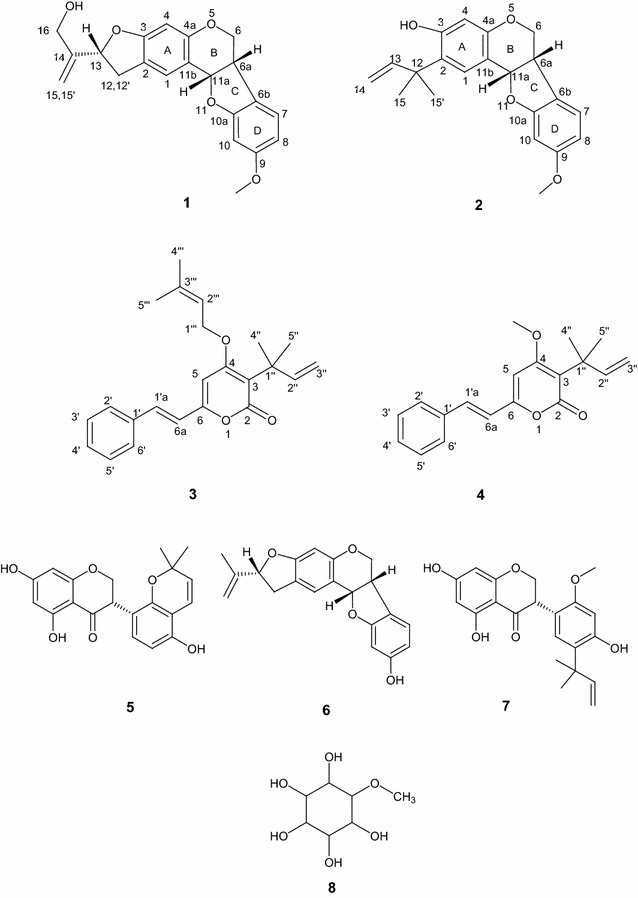
Structures of compounds 1–8

**Fig. 2:**
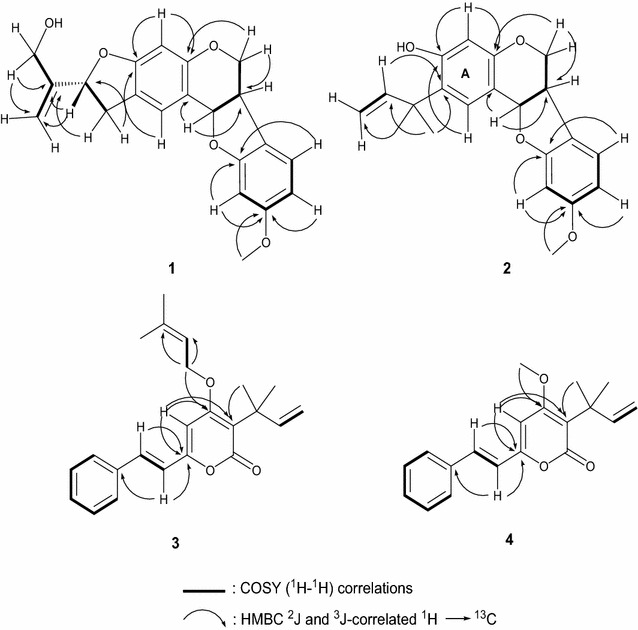
Key HMBC (→) and ^1^H–^1^H COSY (─) correlations of 1–3

Compound **1** was obtained as a brownish powder. Its HR-ESI–MS spectrum showed a pseudo-molecular ion peak ([M+H]^+^ *m*/*z* 353.1353) corresponding to C_21_H_20_O_5_. This elemental composition accounted for 12 (twelve) double bonds equivalents. The IR spectrum of **1** exhibited absorption bands for hydroxyl (3308 cm^−1^), olefines (1618 cm^−1^) and aromatic (1496 cm^−1^). On the ^1^H NMR of **1**, characteristic A/B/C/D patterns of pterocarpans arising from the 6a-, 11a-, 6 eq- and 6ax-hydrogens was observed respectively at δ 3.62 (1H, m, H-6a), 5.55 (1H, d, *J* = 6.0 Hz, H-11a), 4.02 (1H, dd, *J* = 5.4, 2.1 Hz, H-6 eq), and 3.62 (1H, m, H-6ax) suggesting that **1** is a pterocarpan [4]. The ^1^H NMR spectrum (Table 1) also showed signals of five aromatic hydrogens as two singlets at δ 7.29 (1H, s, H-1) and 6.40 (1H, s, H-4) of the ring A and an ABX aromatic system of the ring D at δ 7.25 (1H, d, *J* = 8.4 Hz, H-7), 6.47 (1H, dd, *J* = 8.4, 2.7 Hz, H-8), 6.31 (1H, br s, H-10). Additionally, signals of an hydroxylated 2′-isopropenyl dihydrofuran moiety were clearly displayed at [δ 3.12 (dd, 1H, 15.1, 9.3, H-12); 3.42 (dd, 1H, *J* = 15.1, 7.6, H-12′); 5.37 (t, 1H, *J* = 9.3, H-13); 5.22 (m, 2H, H-15,15′); 4.21(brs, 1H, H-16) and 4.29 (brs, 1H, H-16′)]. The presence on the ^13^C NMR spectrum of carbon signals at δ 149.1 (s, C-14), 109.1 (t, C-15), 84.1 (d, C-13), 34.1 (t, C-12) and 61.4 (t, C-16) confirmed the 2′-isopropenyl dihydrofuran ring core. This partial structure was also supported by the HMBC correlations H-15,15′/C-13-16 and H-16/C-13,-14,-15. The appearance of the two protons of ring A as sharp singlets confirmed that the hydroxylated 2′-isopropenyl dihydrofuran have a linear fusion with ring A of the pterocarpan. This information was supported by the long-range correlations between H-12 with C-1, C-2, C-3; H-1 with C-2, C-3 and H-4 with C-2, C-3. The ^1^H-NMR of **1** also displayed the signal of a methoxyl group as a singlet of three protons at δ 3.77. This substituent was located at the position 9 of the ring D based on the HMBC correlation (Fig. 2) between its hydrogens and C-9 (δ 161.2). The ^13^C-NMR and DEPT spectra of **1** (Table 1), exhibited 21 signals including 8 C, 8 CH, 4 CH_2_ and CH_3_ groups. The above mentioned spectroscopic data were close to those of seputhecarpan B previously identified from the same plant [3]. The only difference was the presence of a MeO group (see Additional file 1) suggesting that compound **1** is the methoxylated derivative of seputhecarpan B. To the best of our knowledge **1** is a new pterocarpan to which the trivial name seputhecarpan C was assigned (Fig. 1; Table 1).

**Table 1:** ^1^H- and ^13^C-NMR Data (300 and 75 MHz, resp) of **1** in (D_6_)acetone^a^ and **2** in CDCl_3_^a^. δ in ppm, *J* in Hertz

Atom	2		1	
	Proton	Carbon	Proton	Carbon
1	7.02 (*s*, 1H)	130.4 (*d*)	7.29 (s, 1H)	126.9 (*d*)
2	–	129.2 (*s*)		120.7 (*s*)
3	–	155.1 (*s*)		161.0 (*s*)
4	6.41 (*s*, 1H)	103.2 (*d*)	6.40 (s, 1H)	96.3 (*d*)
4_a_	–	154.8 (*s*)		156.2 (*s*)
6_ax_	4.11 (*t*, *J* = 10.3, 1H)	70.0 (*t*)	3.62 (*m*, 1H)	66.4 (*t*)
6 _eq_	4.37 (*ddd, J* = 10.3; 3.4; 2.0, 1H)		4.02 (*dd, J* = 5.4; 2.1, 1H)	
6_a_	3.51 (*m*, 1H),	32.2 (*d*)	3.62 (*m*, 1H)	39.5 (*d*)
6_b_	–	117.9 (*s*)		119.5 (*s*)
7	6.99 (*d, J* = 8.2, 1H)	126.3 (*d*)	7.25 (*d, J* = 8.4, 1H)	125.0 (*d*)
8	6.41 (*dd, J* = 8.2; 2.5, 1H)	108.0 (*d*)	6.47 (*dd, J* = 8.4; 2.7, 1H)	106.0 (*d*)
9	–	157.7 (*s*)	–	161.2 (*s*)
16-OH	–		4.02 (*s*, 1H)	
10	6.40 (*d, J* = 2.5 Hz, 1H)	100.7 (*d*)	6.31 (*brs*, 1H)	97.5 (*d*)
10_a_	–	152.5 (*s*)	–	160.9 (*s*)
11_a_	5.02 (*brs,* 1H)	77.2 (*d*)	5.55 (*d, J* = 6.0, 1H)	78.9 (*d*)
11_b_	–	114.8 (*s*)	–	112.6 (*s*)
12	–	40.2 (*s*)	3.42 (*dd, J* = 15.1; 9.3, 1H)	34.1 (*t*)
12′	–		3.12 (*dd, J* = 15.1; 7.6, 1H)	
13	6.18 (*dd, J* = 18.0; 10.1, 1H)	148.2 (*d)*	5.37 (*t, J* = 9.3, 1H)	84.1 (*d*)
14	4.99 (*m*, 2H)	109.7 (*t*)		149.1 (*s*)
15	1.44 (*s*, 3H)	27.3 (*q*)	5.22 (*m*, 1H)	109.1 (*t*)
15′	1.44 (*s*, 3H)		5.22 (*m*, 1H)	
16	–	–	4.21(*brs*, 1H) 4.29 (*brs*, 1H)	61.4 (*t*)
-OMe	3.78 (*s*, 3H),	55.3 (*q*)	3.77 (*s*, 3H)	54.8 (*q*)

Atom numbering as indicated in Fig. 1

^a^All assignments are based on 1H, 1H-COSY, HMQC, and HMBC data

Compound **2** was obtained as yellow oil. Its molecular formula was determined as C_21_H_22_O_4_ ([M + Na]^+^ *m*/*z* 361.1047) based on the HR-ESI–MS data. Comparison of NMR data (see Additional file 2) to those of seputhecarpans A and B, indicated that these compounds are related and have the same A/B/C/D ring system of a pterocarpan [3]. Protons at the para-positions on ring A were observed as singlets at δ 7.02 (1H, s, H-1) and δ 6.41 (1H, s, H-4). Those of the ring D resonated as an ABX aromatic system at δ 6.99 (1H, d, *J* = 8.2 Hz, H-7), 6.41 (1H, dd, *J* = 8.2, 2.5 Hz, H-8) and 6.40 (1H, dd, *J* = 2.5 Hz, H-10). ^13^C-NMR and DEPT data of 2 (Table 1), revealed 21 signals including 8 C, 8 CH, 2 CH_2_ and 3 CH_3_ groups. Four carbinol signals characteristic of the pterocarpan skeleton were observed at δ_H_/δ_C_ 5.02 (1H, brs, H-11a)/77.2, 4.37 (1H, ddd, *J* = 10.4, 3.4 and 2.0 Hz, H-6 eq)/70.0 and 4.10 (1H, t, *J* = 10.3 Hz, H-6ax)/70.0 and 3.51 (1H, m)/32.2. The cross analysis of the ^1^H, ^13^C NMR and HSQC spectra of **2** also showed the presence of a α,α-dimethylallyl group: [δ_H_/δ_C_ 6.18 (1H, dd, *J* = 18.0, 10.1 Hz, H-13)/148.2, 4.99 (2H, m, H-14)/109.7 and 1.44 (6H, s, 2xCH_3_, H-15,15′)/27.3], and a methoxyl group at δ_H_/δ_C_ 3.78 (s)/55.3. Their positions were deduced by the mean of heteronuclear long-range correlations (Fig. 2) of the methoxyl protons at δ 3.78 to C-9 (δ 157.7) and between the gem dimethyls of the α,α-dimethylallyl group at δ 1.44 and C-2 (δ 129.2) Compound **2** turned also to be a new pterocarpan congener to which the trivial name seputhecarpan D was assigned (Fig. 1; Table 1; see Additional file 2).

Compound **3** was obtained as yellow oil. Its molecular formula, C_23_H_26_O_3_ consistent with eleven double bonds equivalents was deduced from its HR-ESI–MS ([M+H]^+^,* m*/*z* 351.1940). IR absorption bands at 1686, 1524, 1348 and 1024 cm^−1^ indicated the presence of a carbonyl of an α,β-unsaturated lactone [5]. The negative ferric chloride test suggested the absence of free phenolic hydroxyl group. NMR data of **3** (see Additional file 3) revealed a singlet at δ_H_/δ_C_ 6.56/98.1 attributed to a CH group in ortho position of two oxygenated aromatic carbons. HMBC correlations of this proton with two oxygenated quaternary carbons at δ 166.5 (C-4) and 157.7 (C-6) coupled with the presence of the carbonyl of the lactone at δ 162.6 confirmed that **3** is a α-pyrone derivative [6]. Moreover, further diagnostic of the NMR data revealed signals of a mono-substituted aromatic ring with two sets of hydrogen at δ 7.51 (m, 2H) and δ 7.39 (m, 3H) and attached to the carbon atoms at δ 127.2 (C-2′/C-6′), δ 128.6 (C-3′/C-5′) and δ 129.0 (C-4′). A γ,γ-dimethylallyl moiety at [δ_H_/δ_C_ 4.71 (2H, d, *J* = 6.6 Hz)/65.9; 5.50 (1H, t, 1.2 Hz)/118.1; 1.83 (3H, s)/24.4 and 1.80 (3H, s)/16.9] and an α,α-dimethylallyl group at [δ_H_/δ_C_ 6.18 (1H, dd, *J* = 17.4, 10.5 Hz)/148.2; 4.95 (2H, m)/109.7 and 1.49 (6H, s, 2 × CH_3_)/27.7] were also observed on the NMR spectra. The downfield chemical shift of the methylene of the γ,γ-dimethylallyl group (δ 4.71) indicated this group to be attached to the pyrone by an ether function. The assumption was supported by HMBC correlations of the CH_2_ group at δ 4.71 with C-4 (δ 167.4). On the other hand, the HMBC correlations of both H-5 (δ 6.14) and the protons of the gem-dimethyl of the α,α-dimethylallyl at δ 1.54 with the quaternary carbon (C-3) at δ 112.2 confirmed the location of this substituent at C-3 (Fig. 2). In addition, trans-olefinic protons were observed at δ 7.44 (d, 1H, *J* = 15.0 Hz, H-1′a)/135.4 and δ 6.88 (d, 1H, *J* = 15.0 Hz, H-6a)/118.7. The downfield resonance of H-1′a compared to H-6a was in accordance with the electrons delocalization induced by the α-pyrone ring. HMBC correlations were observed between both H-1′a and H-6a with δ 157.7 (C-6) and δ 135.4 (C-1′) confirming that the *trans* olefinic carbons were linked to the pyrone ring at C-6 and to the phenyl group (Fig. 2). The foregoing data led to establish the structure of **3** as new pyrone derivative to which the trivial name ptycholopyrone A was assigned (Fig. 1; Table 2).

**Table 2:** ^1^H- and ^13^C-NMR Data (300 and 75 MHz, resp) of **3** in MeOD^**a**^ and **4** in CDCl_3_^**a**^, δ in ppm, J in Hertz

Atom	4		3	
	Proton	Carbon	Proton	Carbon
2	–	162.6 (*s*)	–	163.8 (*s*)
3	–	112.2 (*s*)	–	111.0 (*s*)
4	–	166.5 (*s*)	–	167.5 (*s*)
5	6.14 (*s*, 1H)	96.7 (*d*)	6.56 (s, 1H)	98.1 (*d*)
6	–	157.7 (*s*)	–	157.7 (*s*)
6a	6.63 (*d, J* = 15.2, 1H),	118.7 (*d*)	6.88 (*d, J* = 15.0, 1H)	118.7 (*d*)
1′a	7.53 (*d, J* = 15.2, 1H,)	135.4 (*d*)	7.44 (*d, J* = 15.0, 1H)	134.8 (*d*)
1′	–	135.4 (*s*)		135.5 (*s*)
2′, 6′	7.51 (*m*, 1H)	127.4 (*d*)	7.60 (*m*, 1H)	127.2 (*d*)
3′	7.39 (*m*, 3H)	128.9 (*d*)	7.38 (*m*, 3H)	128.6 (*d*)
4′		129.3 (*d*)		129.0 (*d*)
5′		128.9 (*d*)		128.6 (*d*)
1′′	–	40.1 (*s*)	–	39.6 (*s*)
2′′	6.23 (*dd, J* = 17.4; 10.5, 1H)	148.6 (*d*)	6.18 (*dd, J* = 17.4; 10.5, 1H)	148.4 (*d*)
3′′	4.98 (*dd*, *J* = 17.4; 1.2, 1H) 4.92 (*dd*; *J* = 10.5; 1.2, 1H)	108.4 (*t*)	4.87 (*m*, 2H)	107.2 (*t*)
4′′, 5′′	1.54 (*s*, 6H)	27.7 (*q*)	1.49 (*s*, 6H)	27.0 (*q*)
1′′′	–	–	4.71 (*d, J* = 6.6, 2H)	65.9 (*t*)
2′′′	–	–	5.50 (*t, J* = 1.2, 1H)	118.1 (*d*)
3′′′	–	–	–	139.5 (*s*)
4′′′	–	–	1.83 (*s*, 3H)	24.4 (*q*)
5′′′	–	–	1.80 (*s*, 3H)	16.9 (*q*)
-OMe	3.87(*s*, 3H)	55.9 (*q*)	–	–

Atom numbering as indicated in Fig. 1

^a^All assignments are based on 1H, 1H-COSY, HMQC, and HMBC data

Compound **4** was isolated as a yellow crystal, mp: 104.3–106.2 °C as the major constituent of the plant. Its molecular formula C_19_H_20_O_3_ was deduced from the analysis of HR-ESI–MS in which the pseudo-molecular ion [M+H]^+^ was observed at* m*/*z* 297.1514. NMR data of **4** (see Additional file 4; Table 2) were closely comparable to those of mundulea lactone **4** previously isolated from *Mundulea suberosa* by Dutta [7]. The structure was revised by Lalitha et al. [6] and the full NMR data were reported by Venkata et al. [8]. The ^13^C chemical shifts of 1′a and 6a were correctly assigned in the previous report. However, the ^1^H chemical shifts of H-1′a and H-6a were wrongly assigned at δ 6.55 (d, *J* = 16 Hz) and 7.50 (d, *J* = 16 Hz) respectively. The analysis of the HMQC spectra of **4** revealed correlations between the proton at δ 7.53 (current H-1′a) and the carbon at δ 135.4 and between the proton at δ 6.63 (current H-6a) and the carbon at δ 118.7. This can be justified by the fact that H-1′a is highly deshielded by the conjugation with pyrone ring; therefore, its ^1^H chemical shift should be higher than the one of H-6a. Additionally, the ^13^C chemical shifts of the aromatic oxymethines C-4 and C-6 and the carbonyl of the lactone C-2 were assigned as δ 157.7, 162.6 and 166.6 respectively [8]. We herein revise the above NMR assignment of **4**. Correlations were observed on the HMBC spectrum (Additional file 4) of **4** from the trio H-1′a, H-6a and H-5 to C-6 at δ 157.7. Based on this information, the chemical shift of C-6 was unequivocally assigned at δ 157.7. Furthermore, HMBC correlation was observed between the hydrogen atoms of the methoxyl at δ 3.87 and C-4 at δ 166.5 and no correlation was observed with the carbon at δ 162.6 suggesting that the chemical shift of C-4 and C-2 were respectively δ 166.5 and 162.6. Based on these data, the NMR assignment of mundulea lactone **4** was revised accordingly (Fig. 1; Table 2).

Four others known compounds were isolated: glyasperin F **5** [9], Seputhecarpan A **6** [3], Seputheisoflavone **7** [3] and 5-*O*-methyl-myo-inositol or sequoyitol **8** [10] (Fig. 1).

The anticancer activity of the isolated compounds was evaluated on two lung cancer cell lines A549 and SPC212 (Table 3). The results summarized in Table 3 showed that apart from compound **8**, others (**1**, **2**, **4**–**7**) displayed anti-proliferative effects against the two carcinoma cell lines with IC_50_ values below 75 µM. The recorded IC_50_ ranged from 11.39 µM (for compound **4**) to 73.49 µM (for compound **1**) towards A549 cells and from 0.59 µM (for compound **7**) to 63.47 µM (for compound **1**) towards SPC212 cells. A threshold of 4 µg/mL or 10 μM IC_50_ value after 48 and 72 h incubation has been set to identify sufficiently cytotoxic molecules [11–13]. IC_50_ values below 10 µM were obtained with **4**, **6** and **7** in SPC212 cells. However, doxorubicin, the reference anticancer drug had better cytotoxic effects than all tested compounds. These data suggest that compounds from *Ptycholobium contortum* and mostly **4**, **6** and **7** can be exploited in the fight against lung cancer.

**Table 3:** Cytotoxicity of compounds and doxorubicin towards lung carcinoma cells

Compounds	Cell lines and IC_50_ values (µM)	
	A549	SPC212
1	73.49 ± 8.64	63.47 ± 5.99
2	26.39 ± 1.27	12.99 ± 0.95
4	11.39 ± 1.52	*9.02* *±* *0.07*
5	13.19 ± 1.55	16.38 ± 1.89
6	46.70 ± 3.63	*9.35* *±* *0.98*
7	38.68 ± 3.65	*0.59* *±* *0.16*
8	>425.53	>425.53
Doxorubicin	1.01 ± 0.20	0.07 ± 0.00

Values in italics significant cytotoxic effect [13]

## Experimental part

### General comments

NMR spectra were recorded on Bruker DMX Avance 300 and 600 instruments equipped with an auto-tune probe and using the automation mode aided by the Bruker program, Icon-NMR using Acetone-*d6*, CDCl_3_ and CD_3_OD as solvents and internal standards. HR EISMS spectra were determined on a microTOF-Q 98 spectrometer. Infra-Red spectra were recorded as KBr disk. For column chromatography, silica gel 60 particles size 0.04–0.063 mm (Merck) or Sephadex LH-20 (Sigma) were used. Analytical and Preparative TLC were performed respectively using silica gel 60 PF_254 + 366_ (Merck) and silica gel 60-F_254_ precoated aluminum sheets (Merck). The plates were visualized using UV (254 and 366 nm) and revealed by spraying with vanillin-sulphuric acid.

### Plant material

The roots of *P. contortum* were collected around Maun, Ngamiland District in North-Western Botswana and were botanically authenticated by Joseph Madome of the Okavango Research Institute (ORI) Herbarium. Voucher specimen (No KM-1-Maun-2013; KM-2-Maun-2014) were deposited at the University of Botswana Herbarium and at ORI Herbarium, respectively.

### Extraction and isolation

Dried and powdered stem bark of *P. contortum* (1255 g) were extracted twice at room temperature with 4L of CH_2_Cl_2_–MeOH (1:1) for 48 h. The solvent was evaporated under reduced pressure to give 20.53 g of crude extract. The residue was extracted with 2 L of MeOH at room temperature for 24 h to give 7.39 g of crude extract. The two extracts were combined on the basis of their TLC profile to give 27.92 g of crude extract. This extract was defatted with *n-*hexane to give 4.33 g of *n-*hexane fraction. The residue was suspended in H_2_O and partitioned between CHCl_3_ (300 mL × 3), AcOEt (300 mL × 3) and *n-*butanol (300 mL × 3) to give 8.05 g of CHCl_3_; 12.41 g of AcOEt and 1.52 g of *n-*BuOH fractions. The chloroform fraction was subjected to silica gel column chromatography (40–63 μm, 4.5 × 50 cm) using *n-*hexane-AcOEt gradients as eluents. 83 fractions of 300 ml each were collected and combined on the basis of their TLC profile to give 9 sub-fractions (*F*_*1*_–*F*_*9*_) as follows *F*_*1*_ [(1–10), *n-*hexane-AcOEt 5 %, 0.80 g], *2* [(11–19), *n-*hexane-AcOEt 7.5 % 1.20 g], *3* [(20–27), *n-*hexane-AcOEt 10 %, 1.01 g], *4* [(28–49), *n-*hexane-AcOEt 15 %, 1.03 g], *5* [(50–55), *n-*hexane-AcOEt 20 % 0.60 g], *6* [(55–68), *n-*hexane-AcOEt 25 %, 1.05 g], *7* [(69–75), *n-*hexane-AcOEt 30 %, 0.50 g] *8* [(76–80), AE, 0.75 g] and *9* [(81–83), MeOH, 0.30 g] Purification of *F*_*1*_ by a preparative TLC plate afforded **3** [UV (+), Rf = 0.70 at Hex-AE 10 %, *2.1* *mg*], a yellow compound, The yellow precipitate in *F*_*2*_ was washed with Hex-AE 2.5 % followed by a filtration to yield **4** [UV (+); Rf = 0.33 at Hex-AE 10 %, *640.0* *mg*]. *F*_*3*_–*F*_*4*_ were subjected to silica gel column chromatography (40–63 μm, 4.5 × 50 cm) using *n-*hexane-AcOEt gradients as eluents. *F*_*3*_ afforded **6** [UV (+); Rf = 0.30 at Hex-AE 20 %, *12.3* *mg*] while **1** [UV (-); Rf = 0.50 at Hex-AE 20 %, *28.5* *mg*] and **7** [UV (+); Rf = 0.40 at Hex-AE 25 %, *32.0* *mg*] were isolated from *F*_*4*_ respectively as yellowish and brownish powders. *F*_*5*_ was purified using Sephadex LH-20 with CHCl_3_–MeOH (7:3) as eluent to afford **2** [UV (+); Rf = 0.35 at Hex-AE 20 %, *26.7* *mg*] as a red oil and **5** [UV (+); Rf = 0.30 at Hex-AE 20 %, *10.2* *mg*] as a white powder. Precipitate in *F*_*8*_ was washed twice with a mixture of Hexane–ethyl acetate (1:3) and compound **8** was obtained as a white powder. The *n-*hexane fraction (3.76 g) was absorbed on a silica gel and chromatographed on a silica gel column using a mixture of hexane–ethyl acetate of increasing polarity as eluent. From this fraction, compound **4** *(45.7* *mg)* was also re-isolated.

**Seputhecarpan C** (**1**). Brownish crystals. M.p. 108.5–109.9 °C. UV (acetone) λmax nm (log ε): 345 (3.73), 320 (3.67). IR KBr ν (cm^−1^): 3308, 1618, 1496, 963, 814. CD (c 5.0 × 10 ^−3^, MeOH): ([θ_230_] −44,925, [θ_300_] +10,135), [θ_475_] + 3885. ^1^H-and ^13^C-NMR: see Table 1. HR-ESI–MS: 353.1353 *([M+H]* ^+^, C_21_H_21_O_5_^+^; calc. 353.1389), 375.1178 (*[M* + *Na]* ^+^, C_21_H_20_O_5_Na^+^; calc. 375.1208).

**Seputhecarpan D** (**2**). Yellowish oil. UV (acetone) λmax nm (log ε): 340 (4.35), 320 (3.38), 324 (4.40). IR KBr ν (cm^−1^): 3395, 1610, 1490, 1215, 1150, 1080, 965, 902, 836. ^1^H-and ^13^C-NMR: see Table 1. HR-ESI–MS: 361.1047 (*[M* + *Na]* ^+^, C_21_H_22_O_4_Na^+;^ calc. 361.1416),

**Ptycholopyrone A** (=*4*-*(3*-*methylbut*-*2*-*enyloxy)*-*3*-*(2*-*methylbut*-*3*-*en-2*-*yl)*-*6*-*styryl*-*2H*-*pyran*-*2*-*one* **; 3**). Yellow oil. IR KBr ν (cm^−1^): 2956, 1686, 1524, 1348, 1024, 909, 685. ^1^H-and ^13^C-NMR: see Table 2. HR-ESI–MS: 351.1940 *([M+H]* ^+^, C_23_H_27_O_3_^+^; calc. 351.1960), 701.3817 (*[2* *M+H*]^+^, *C*_*46*_ *H*_*53*_ *O*_*6*_^+^; calc. 701.3842).

**Mundulea lactone** (=*4*-*methoxy*-*3*-*(2*-*methylbut*-*3*-*en*-*2*-*yl)*-*6*-*styryl*-*2H*-*pyran*-*2*-*one* **; 4**). Yellow crystals. M.p. 104.3-106.2 °C. IR KBr ν (cm^−1^): 2959, 1686, 1523, 1348, 1080, 909, 685. ^1^H-and ^13^C-NMR: see Table 2. HR-ESI–MS: 297.1514 *([M+H]* ^+^, C_19_H_21_O_3_^+^; calc. 297.1491), 593.2901 (*[2* *M+H]* ^+^, C_38_H_41_O_6_^+^; calc. 593.2903).

### Cell lines and culture

Two lung cancer cell lines were used in this study. They include the human non-small cell lung cancer (NSCLC) cell line A549, obtained from Institute for Fermentation, Osaka (IFO, Japan) and the human mesothelioma cell line, SPC212 provided by Doc. Dr. Asuman Demiroğlu Zergeroğlu, Department of Molecular Biology and Genetic, Gebze Technical University, Turkey. The cells were maintained as a monolayer in DMEM (Sigma-aldrich, Munich, Germany) medium supplemented with 10 % fetal calf serum and 1 % penicillin (100 U/mL)-streptomycin (100 μg/mL) in a humidified 5 % CO_2_ atmosphere at 37 °C.

### Neutral red uptake assay

The cytotoxicity of compounds and doxorubicin (purchased from Sigma Chemical Co., St. Louis, MO, USA) used as standard anticancer drug was performed by neutral red assay as previously described [14]. This method is based on the ability of viable cells to incorporate and bind the supravital dye neutral red in the lysosomes. The procedure is cheaper and more sensitive than other cytotoxicity tests [15]. Compounds were added in the culture medium so that dimethylsulfoxide (DMSO) used prior for dilution did not exceed 0.1 % final concentration. The viability was evaluated based on a comparison with untreated cells. IC_50_ values represent the sample’s concentrations required to inhibit 50 % of cell proliferation and were calculated from a calibration curve by linear regression using Microsoft Excel [16, 17].

## Conclusions

This work reports the chemical investigation of the non polar fractions of *Ptycholobium contortum* from which two new pterocarpans and a new pyrone derivative were isolated. The interesting cytotoxic activities obtained with mundulea lactone **4** seputhecarpan A **6** and seputheisoflavone **7** (IC50 values below 10 µM) gives evidence that the genus *Ptycholobium* is a rich source of prenylated flavonoids and pyrone derivatives with potent cytotoxic activities. These results open a way for the study of the two others species of this genus *P. plicatum* and *P. biflorum* on which no phytochemical nor pharmacological studies have been carried out so far.

## Additional files

10.1186/s13065-016-0204-x Comparision of ^1^H and ^13^C NMR spectra of seputhecarpan C **1** and seputhecapan B (Fotso et *al*, 2013). These spectra clearly show the presence of an additional methoxyle group in seputhecarpan C.

10.1186/s13065-016-0204-x ^1^H and ^13^C NMR spectra of seputhecarpan D. **2**.

10.1186/s13065-016-0204-x ^1^H and ^13^C NMR spectra of pythylopyrone A **3** showing the signals of the additional γ,γ-dimethylallyle group in position 4 of the molecule.

10.1186/s13065-016-0204-x ^1^H and ^13^C NMR spectra of mundulea lactone **4** as well as all the 2D NMR data justifying the revision of the NMR assignment of this compound as shown in Table 2.
