# Supplementary material for: Two new pterocarpans and a new pyrone derivative with cytotoxic activities from Ptycholobium contortum (N.E.Br.) Brummitt (Leguminosae): revised NMR assignment of mundulea lactone
Source: Chem Cent J. 2016 Oct 5;10:58. doi: 10.1186/s13065-016-0204-x (PMC5050614; doi:10.1186/s13065-016-0204-x)
Supplement: Supplementary file 1 — 10.1186/s13065-016-0204-x Comparision of 1H and 13C NMR spectra of seputhecarpan C 1 and seputhecapan B (Fotso et al, 2013). These spectra clearly show the presence of an additional methoxyle group in seputhecarpan C. [file 13065_2016_204_MOESM1_ESM.pdf]

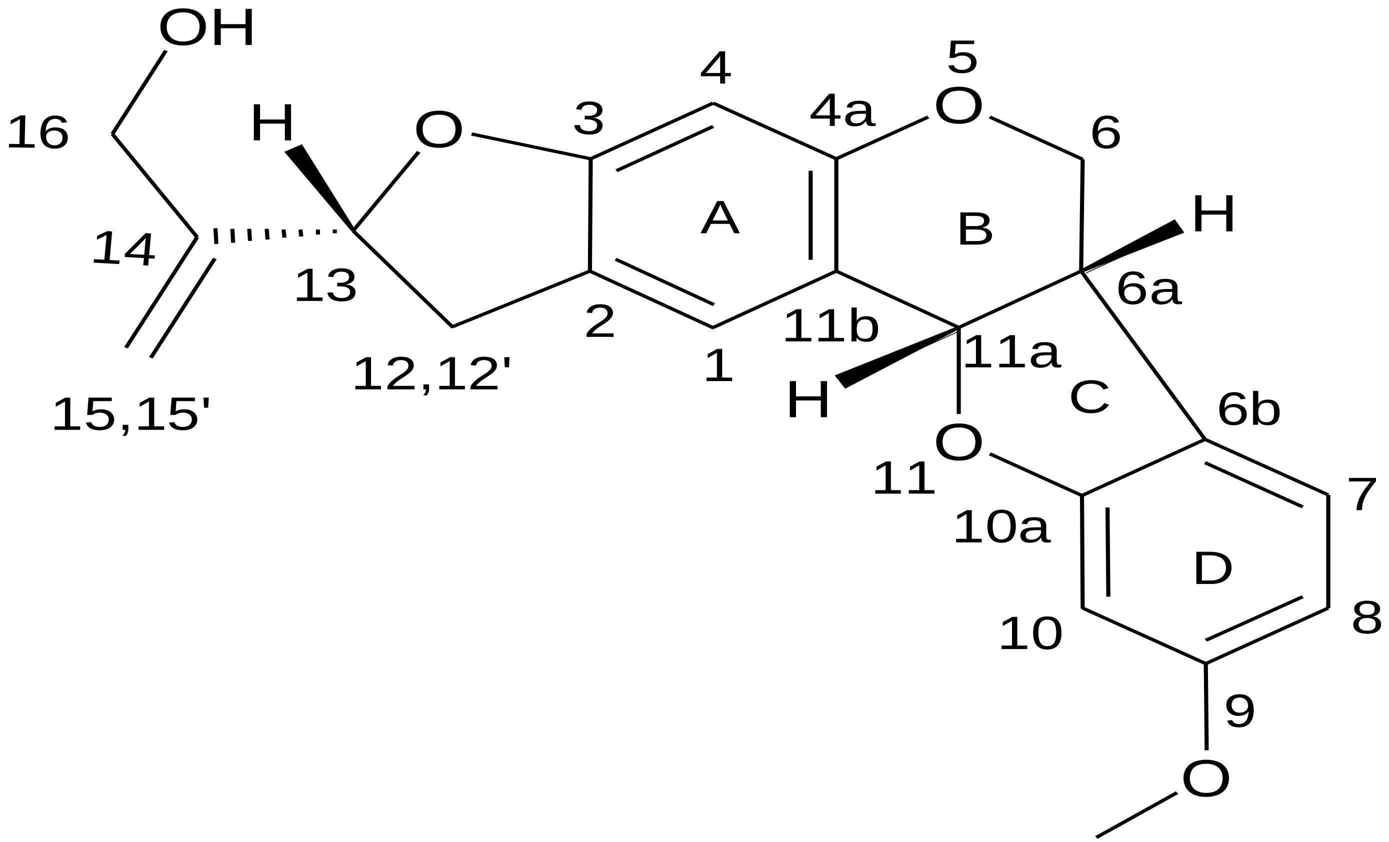

1

Seputhecarpan C

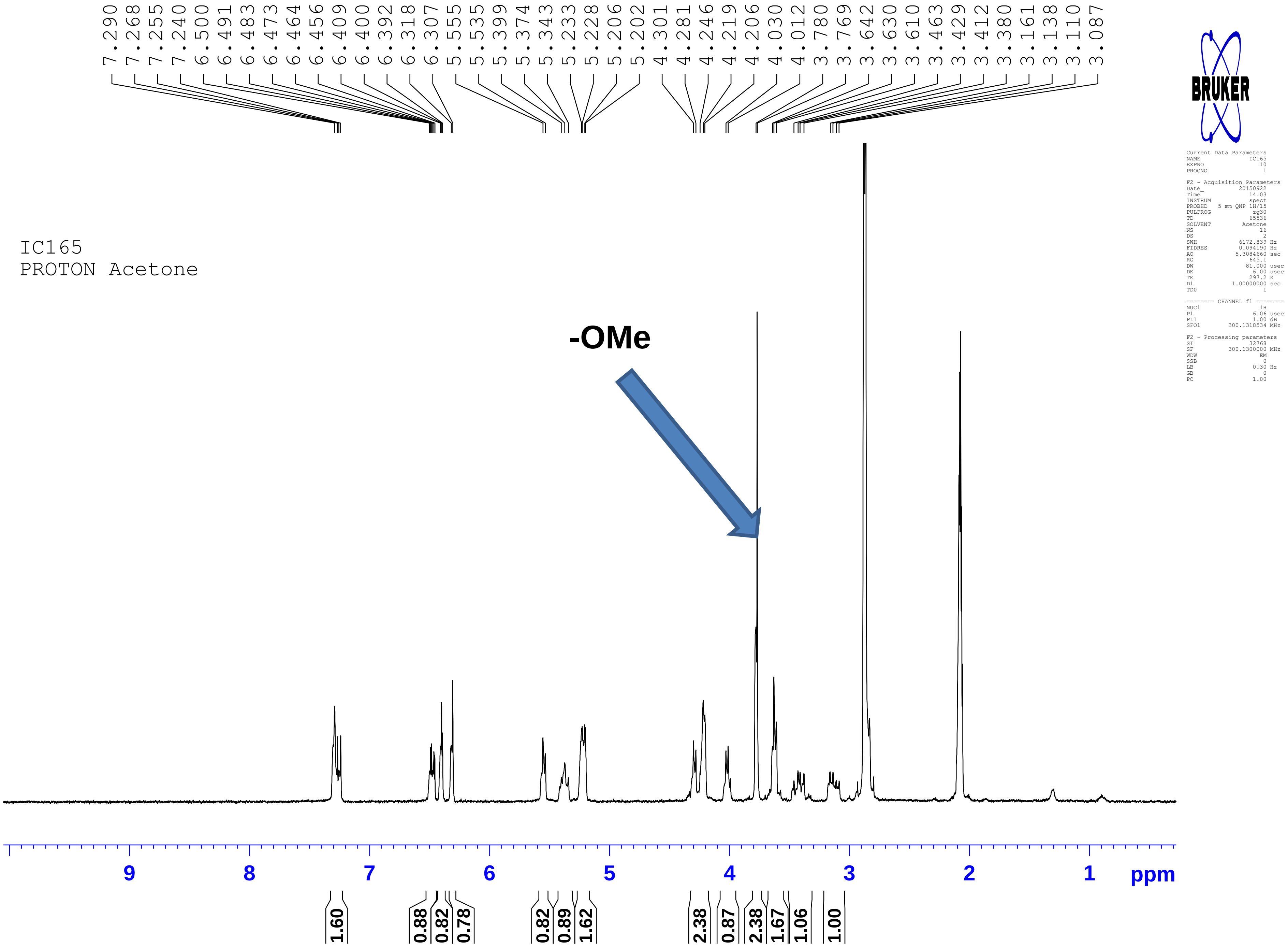

7.290 7.268 7.255 7.240 6.500 6.491 6.483 6.473 6.464 6.456 6.409 6.400 6.392 6.318 6.307 5.555 5.535 5.399 5.374 5.343 5.233 5.228 5.206 5.202 4.301 4.281 4.246 4.219 4.206 4.030 4.012 3.780 3.769 3.642 3.630 3.610 3.463 3.429 3.412 3.380 3.161 3.138 3.110 3.087
IC165
PROTON Acetone
-OMe
BRUKER
Current Data Parameters
NAME IC165
EXPNO 10
PROCNO 1
F2 - Acquisition Parameters
Date_ 20150922
Time 14.03
INSTRUM spect
PROBHD 5 mm QNP 1H/15
PULPROG zg30
TD 65536
SOLVENT Acetone
NS 16
DS 2
SWH 6172.839 Hz
FIDRES 0.094190 Hz
AQ 5.3084660 sec
RG 645.1
DW 81.000 usec
DE 6.00 usec
TE 297.2 K
D1 1.00000000 sec
TD0 1
======== CHANNEL f1 ========
NUC1 1H
P1 6.06 usec
PL1 1.00 dB
SFO1 300.1318534 MHz
F2 - Processing parameters
SI 32768
SF 300.1300000 MHz
WDW EM
SSB 0
LB 0.30 Hz
GB 0
PC 1.00
9 8 7 6 5 4 3 2 1 ppm
1.60 0.88 0.82 0.78 0.82 0.89 1.62 2.38 0.87 2.38 1.67 1.06 1.00

IC165
PROTON Acetone

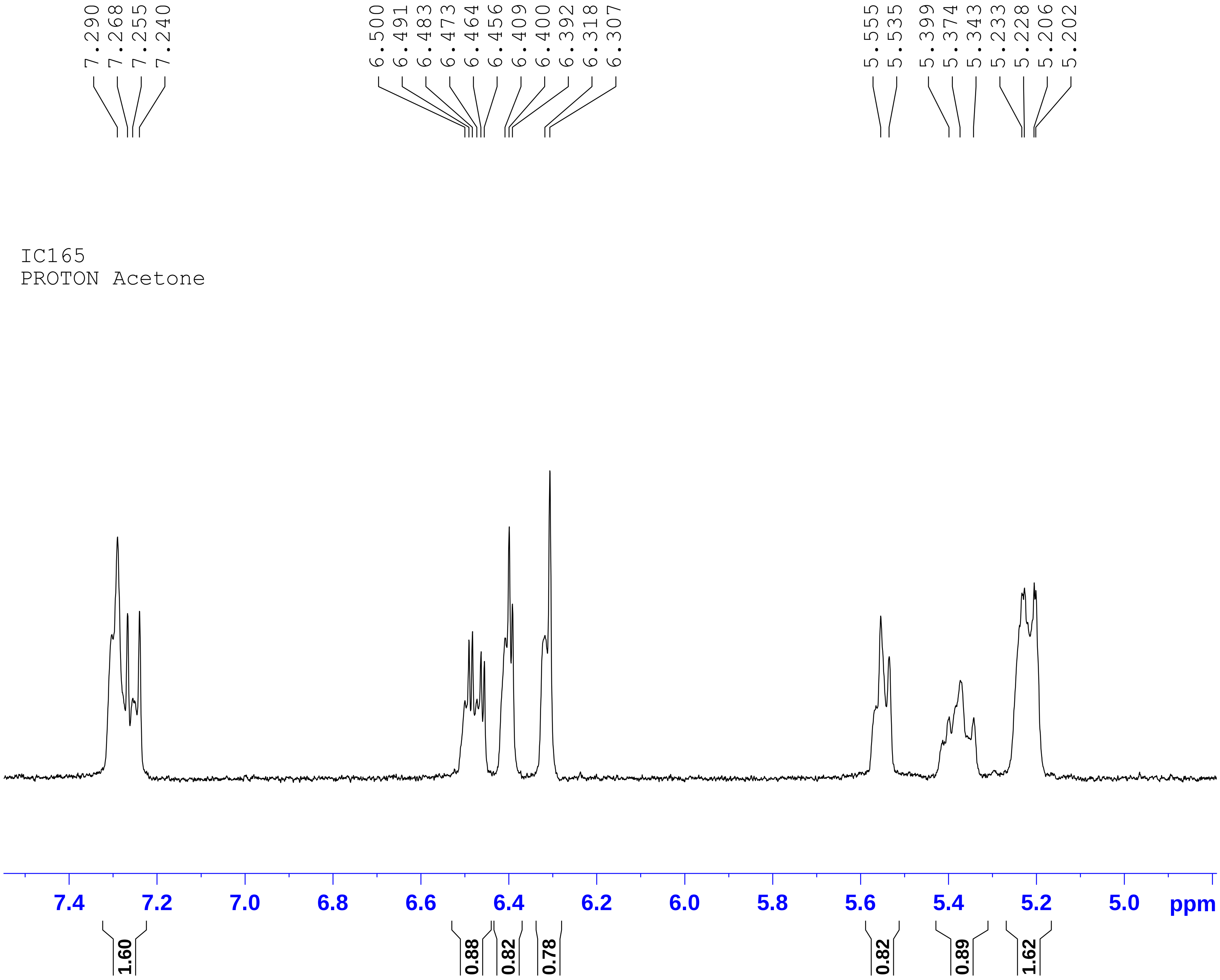

Current Data Parameters
NAME IC165
EXPNO 10
PROCNO 1

F2 - Acquisition Parameters
Date_ 20150922
Time 14.03
INSTRUM spect
PROBHD 5 mm QNP 1H/15
PULPROG zg30
TD 65536
SOLVENT Acetone
NS 16
DS 2
SWH 6172.839 Hz
FIDRES 0.094190 Hz
AQ 5.3084660 sec
RG 645.1
DW 81.000 usec
DE 6.00 usec
TE 297.2 K
D1 1.00000000 sec
TD0 1

======== CHANNEL f1 ========
NUC1 1H
P1 6.06 usec
PL1 1.00 dB
SFO1 300.1318534 MHz

F2 - Processing parameters
SI 32768
SF 300.1300000 MHz
WDW EM
SSB 0
LB 0.30 Hz
GB 0
PC 1.00

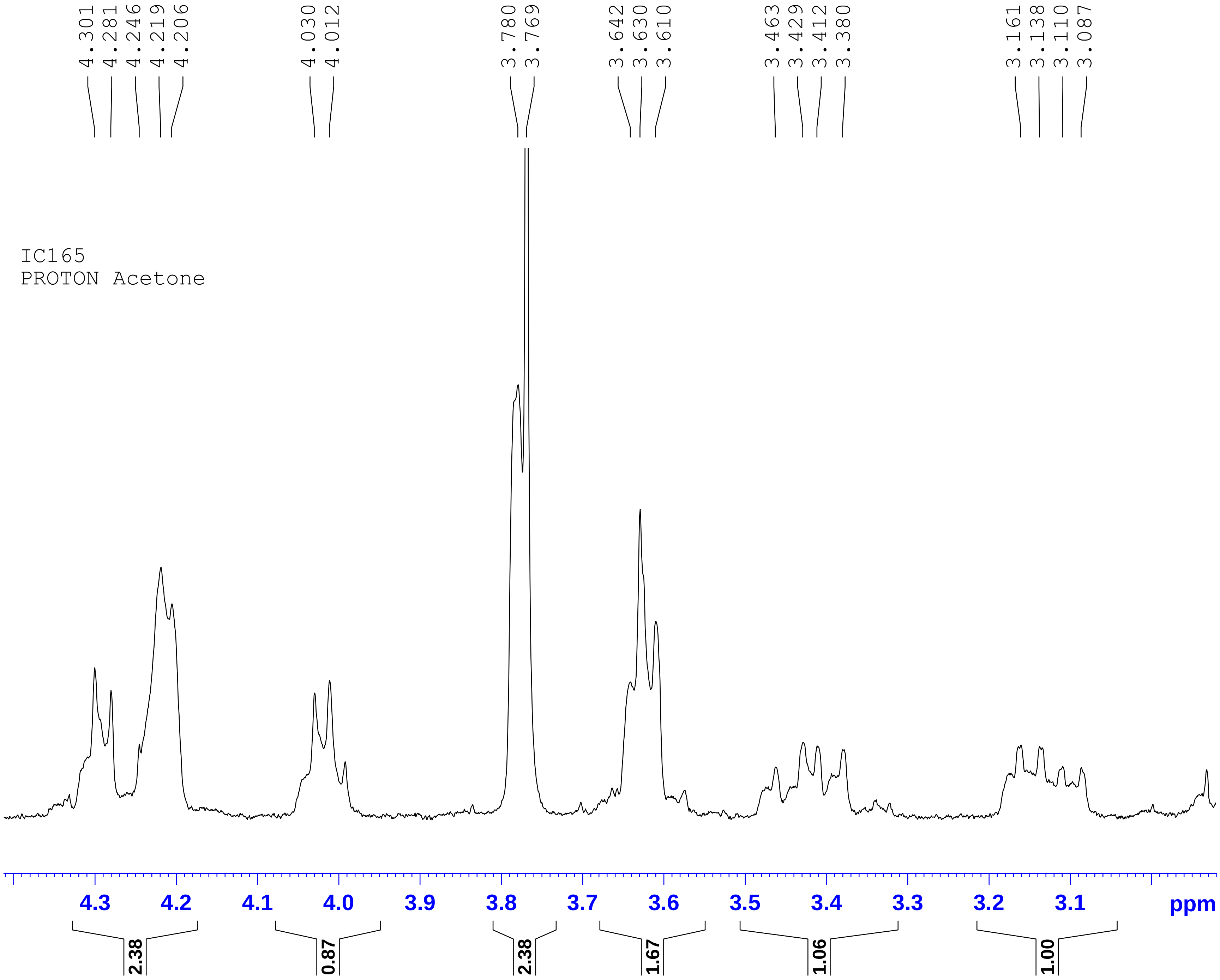

Current Data Parameters
NAME IC165
EXPNO 10
PROCNO 1

F2 - Acquisition Parameters
Date_ 20150922
Time 14.03
INSTRUM spect
PROBHD 5 mm QNP 1H/15
PULPROG zg30
TD 65536
SOLVENT Acetone
NS 16
DS 2
SWH 6172.839 Hz
FIDRES 0.094190 Hz
AQ 5.3084660 sec
RG 645.1
DW 81.000 usec
DE 6.00 usec
TE 297.2 K
D1 1.00000000 sec
TD0 1

======== CHANNEL f1 ========
NUC1 1H
P1 6.06 usec
PL1 1.00 dB
SFO1 300.1318534 MHz

F2 - Processing parameters
SI 32768
SF 300.1300000 MHz
WDW EM
SSB 0
LB 0.30 Hz
GB 0
PC 1.00

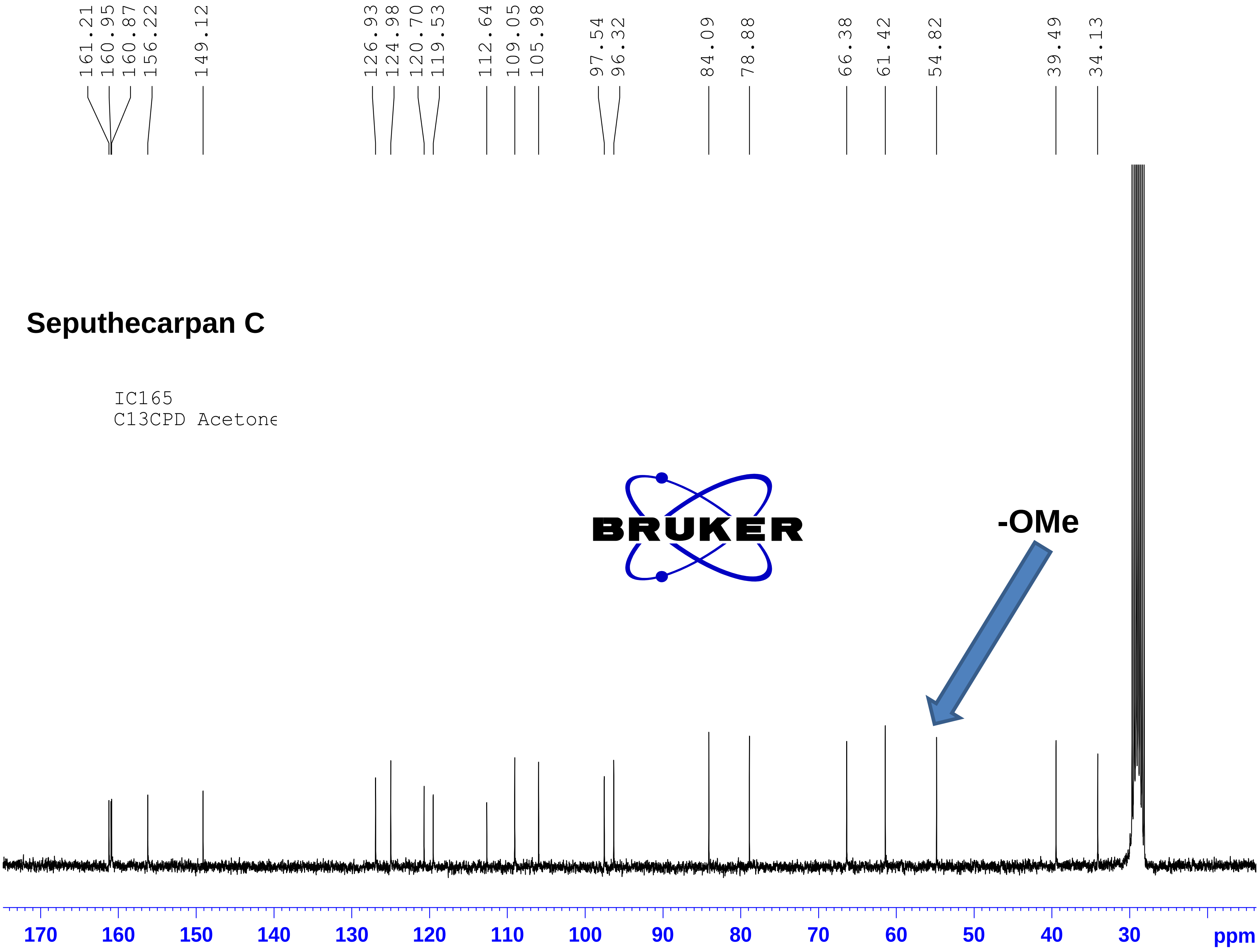
161.21
160.95
160.87
156.22
149.12
126.93
124.98
120.70
119.53
112.64
109.05
105.98
97.54
96.32
84.09
78.88
66.38
61.42
54.82
39.49
34.13
Seputhecarpan C
IC165
C13CPD Acetone
-OMe
170
160
150
140
130
120
110
100
90
80
70
60
50
40
30
ppm

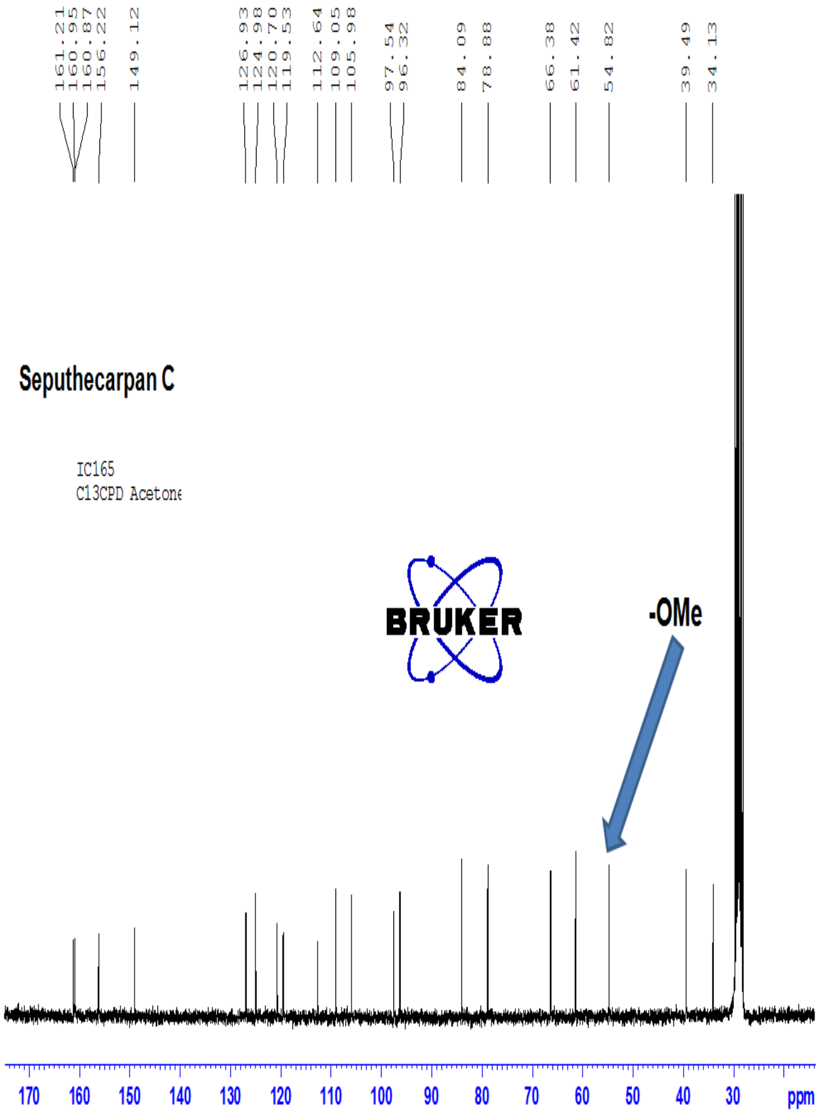
Seputhecarpan C
IC165
C13CPD Acetone
161.21
160.95
160.87
156.22
149.12
126.93
124.98
120.70
119.53
112.64
109.05
105.98
97.54
96.32
84.09
78.88
66.38
61.42
54.82
39.49
34.13
BRUKER
-OMe
170 160 150 140 130 120 110 100 90 80 70 60 50 40 30 ppm

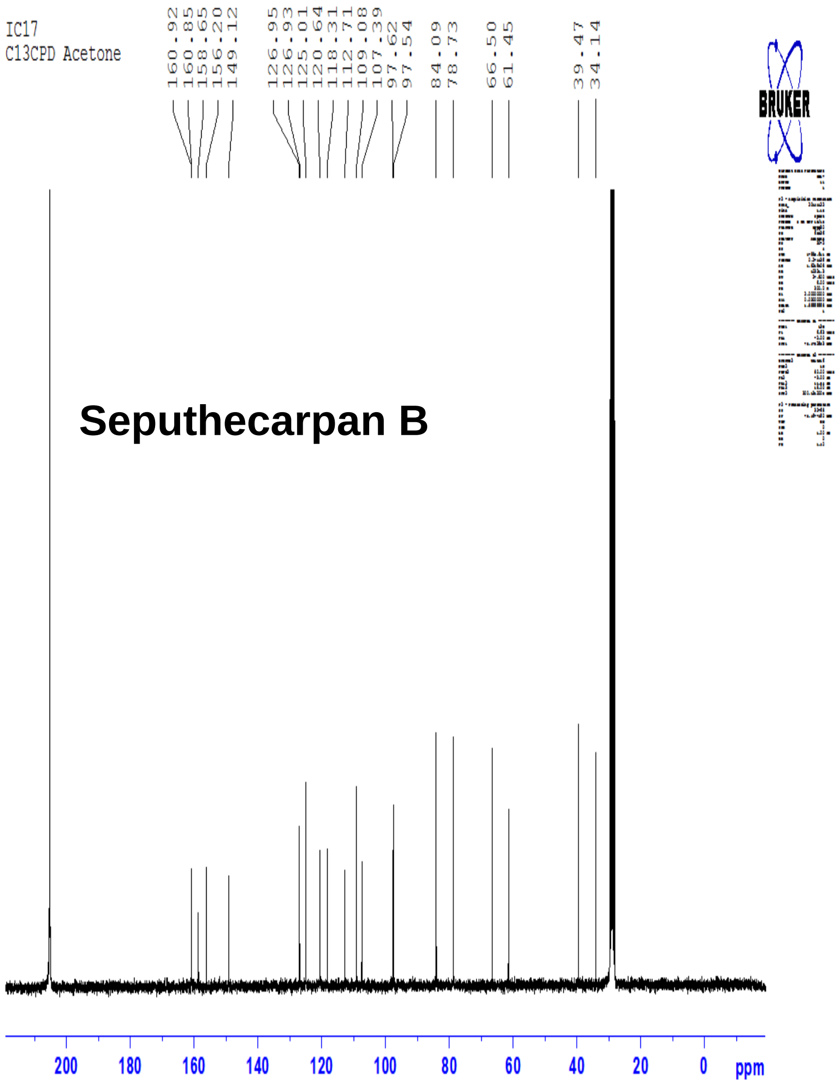
IC17
C13CPD Acetone
Seputhecarpan B
160.92
160.85
158.65
156.20
149.12
126.95
126.93
125.01
120.64
118.31
112.71
109.08
107.39
97.62
97.54
84.09
78.73
66.50
61.45
39.47
34.14
BRUKER
200 180 160 140 120 100 80 60 40 20 0 ppm

IC165
C13DEPT135 Acetone

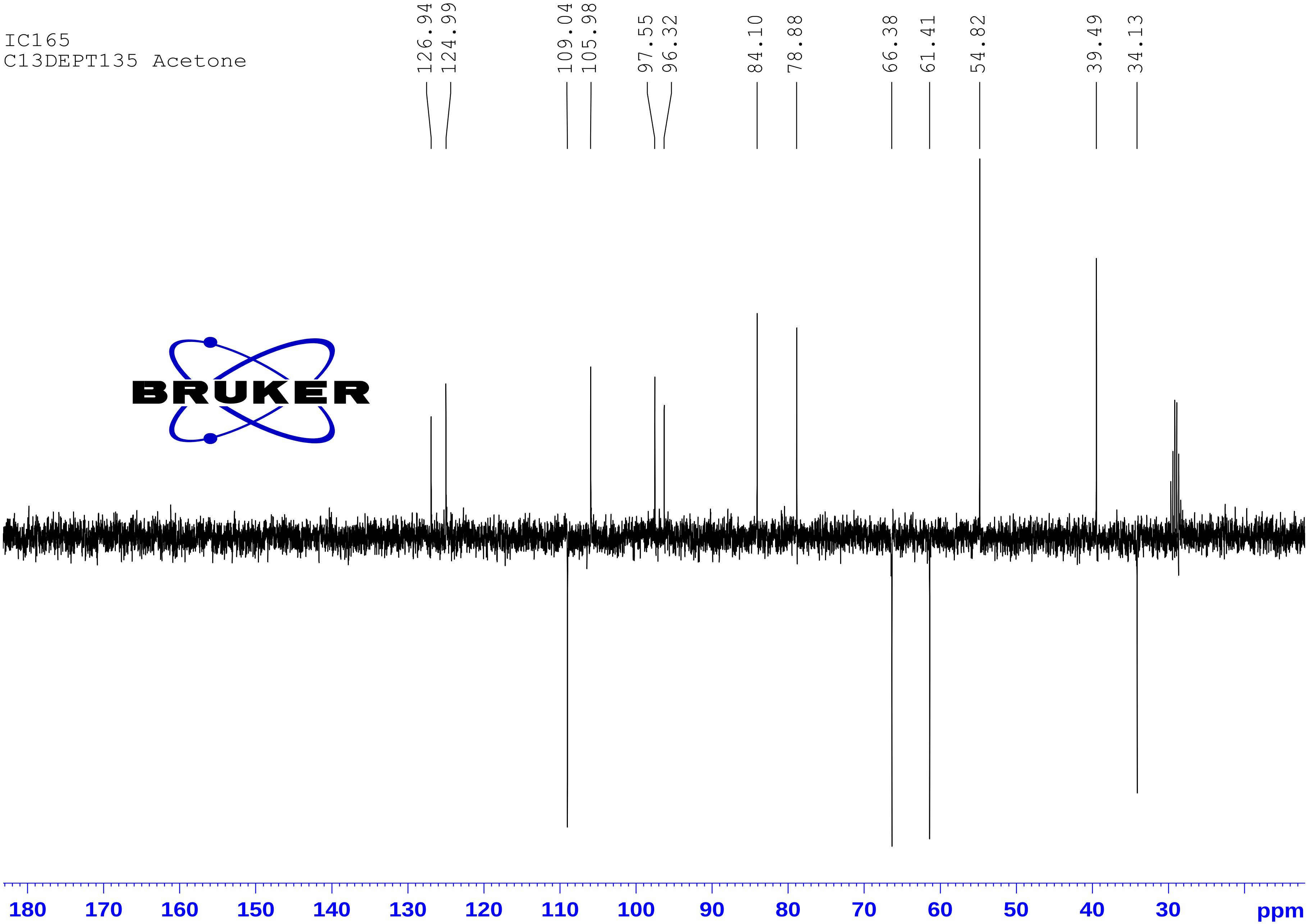

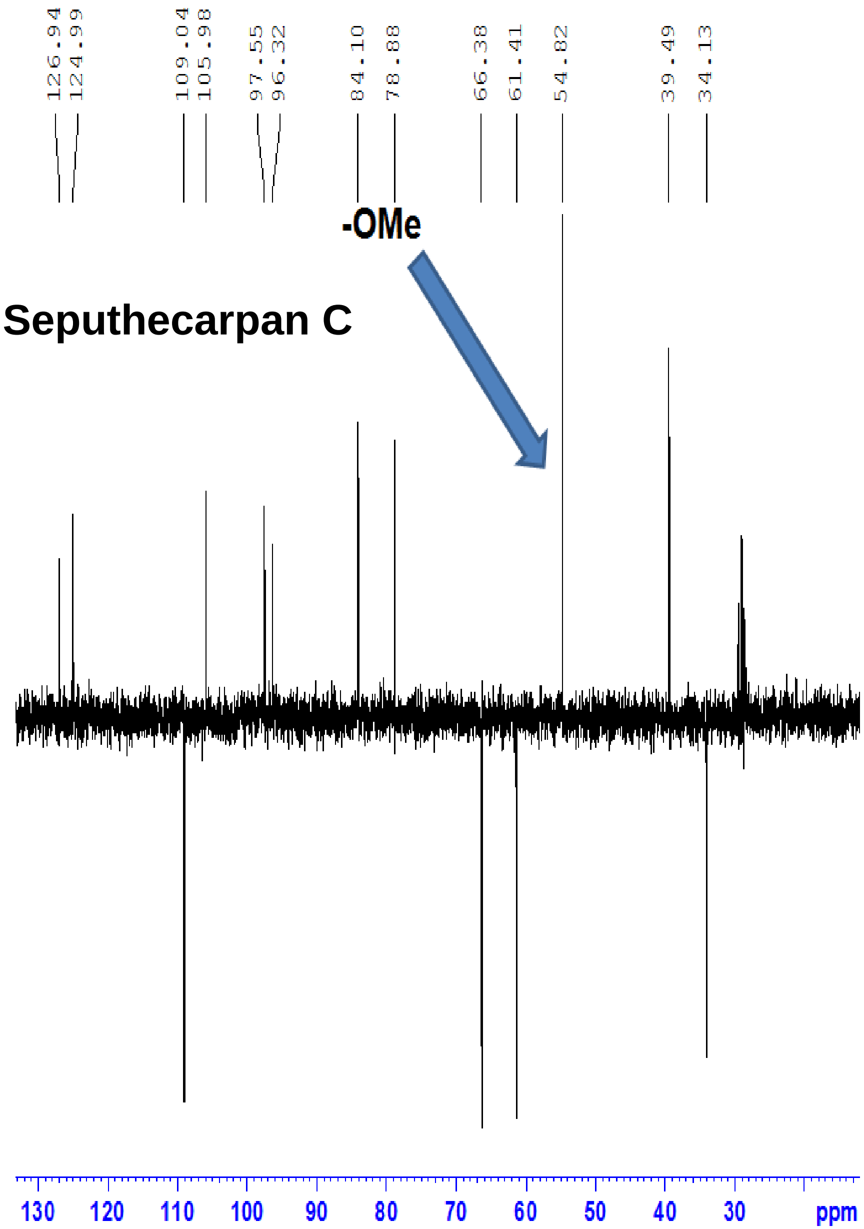
126.94
124.99
109.04
105.98
97.55
96.32
84.10
78.88
66.38
61.41
54.82
39.49
34.13
-OMe
Seputhecarpan C
130 120 110 100 90 80 70 60 50 40 30 ppm

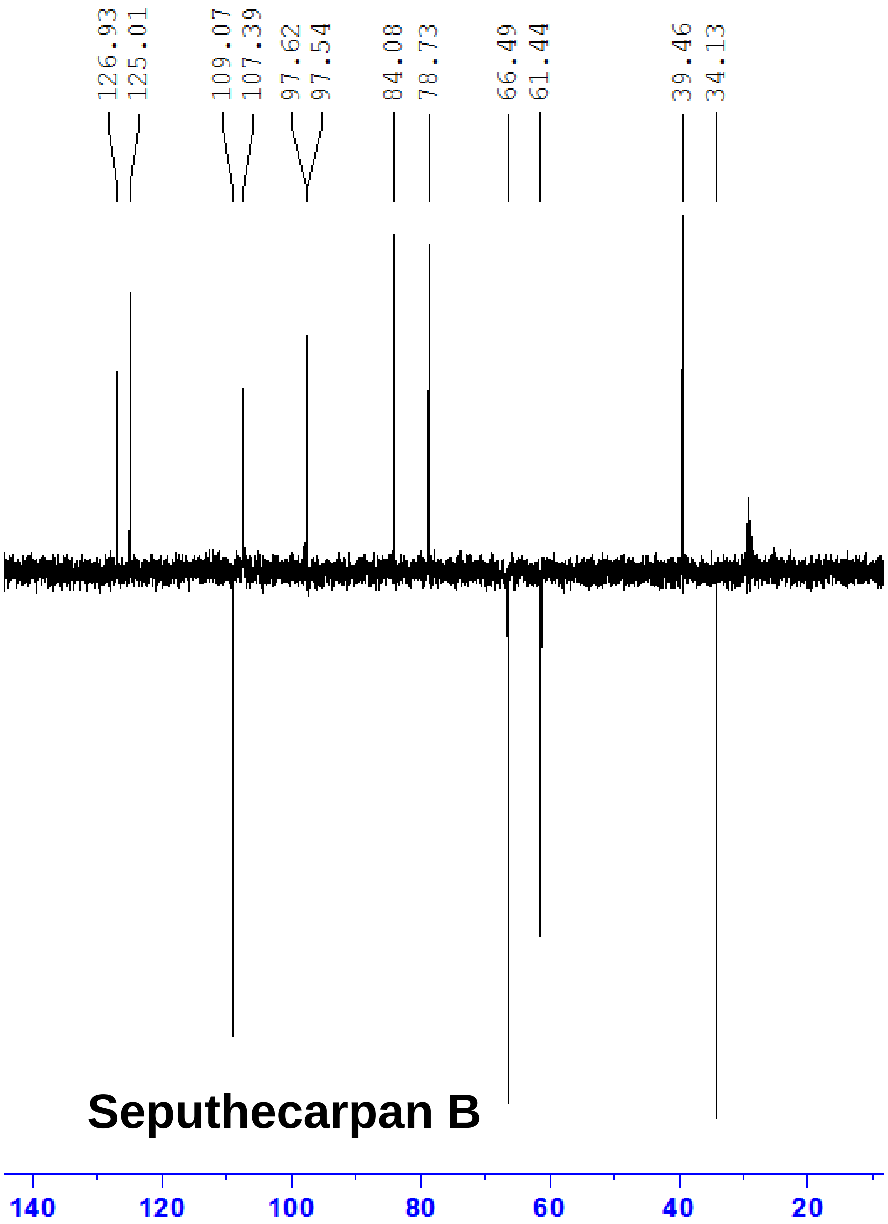
126.93
125.01
109.07
107.39
97.62
97.54
84.08
78.73
66.49
61.44
39.46
34.13
Seputhecarpan B
140 120 100 80 60 40 20

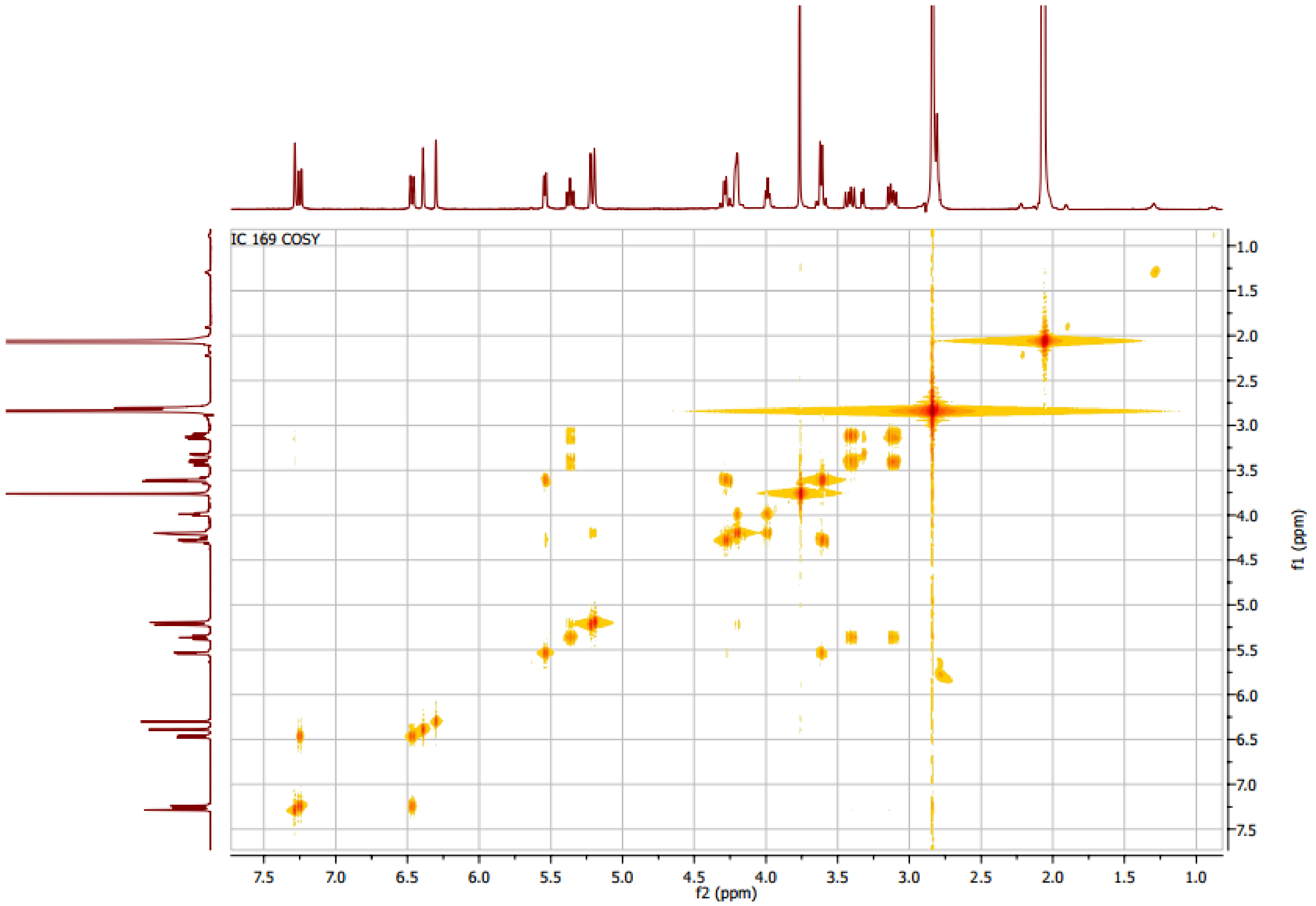

Full COSY spectrum of compound 1

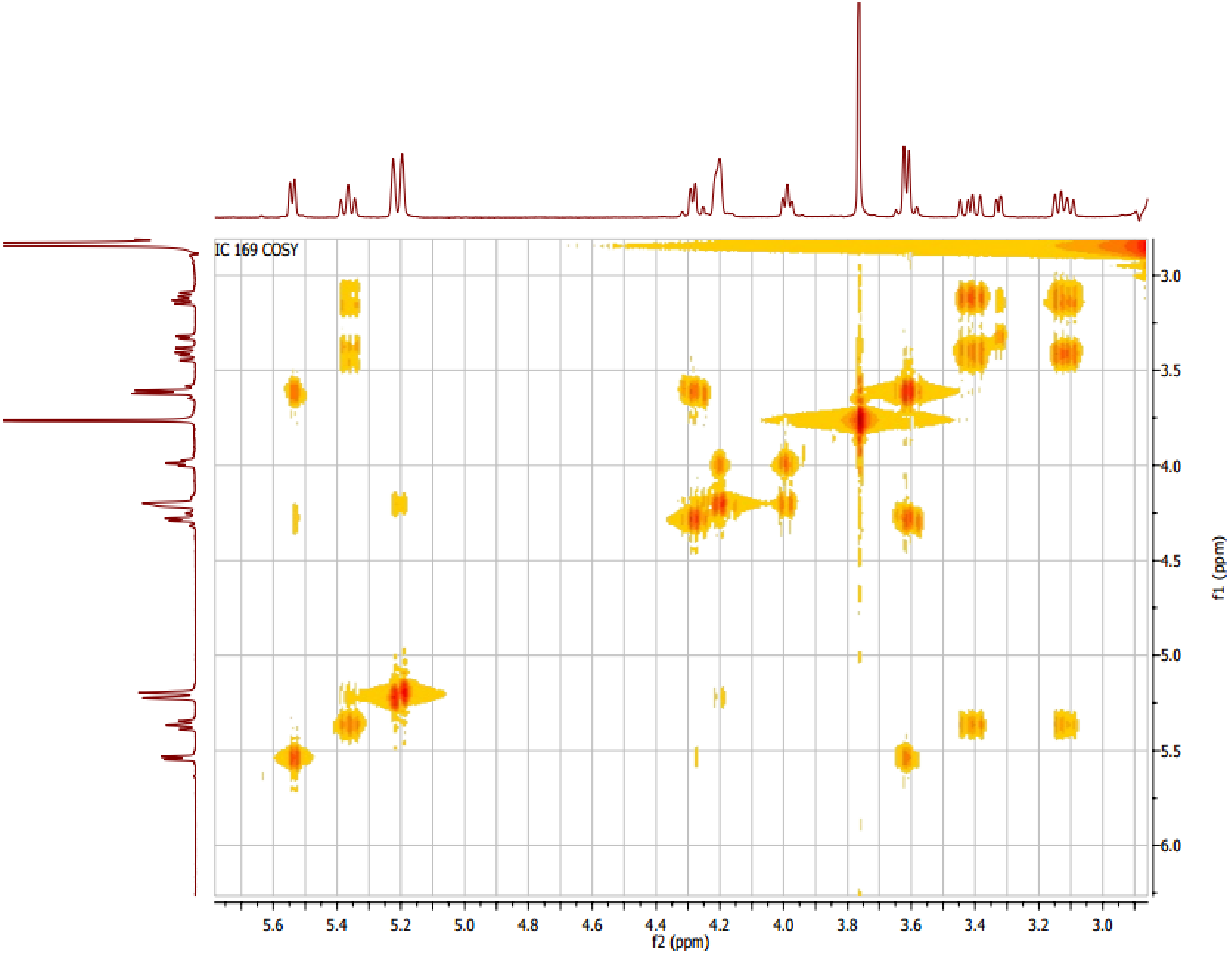

COSY spectrum of compound 1 (aliphatic region)

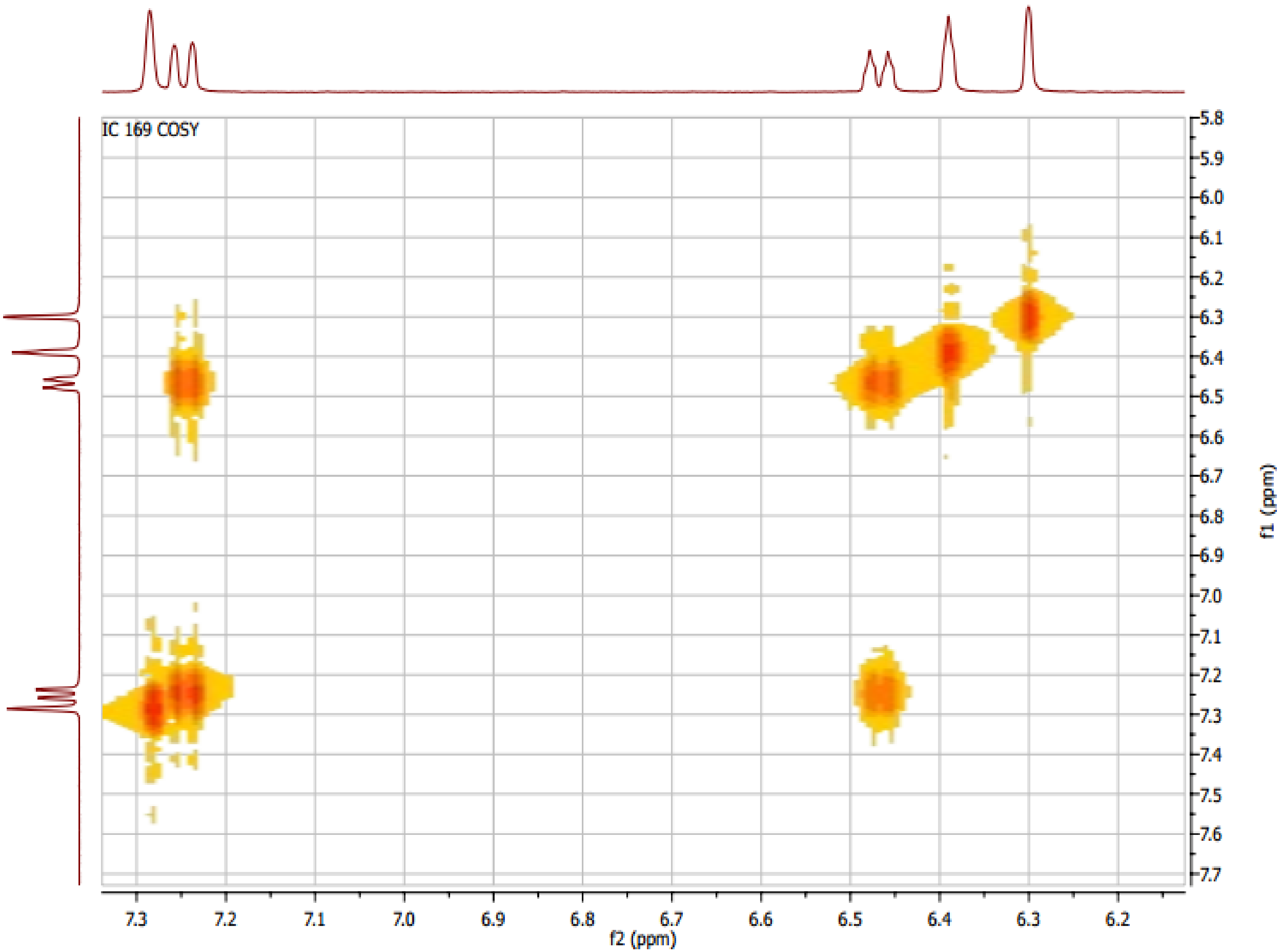

COSY spectrum of compound 1 (aromatic region)

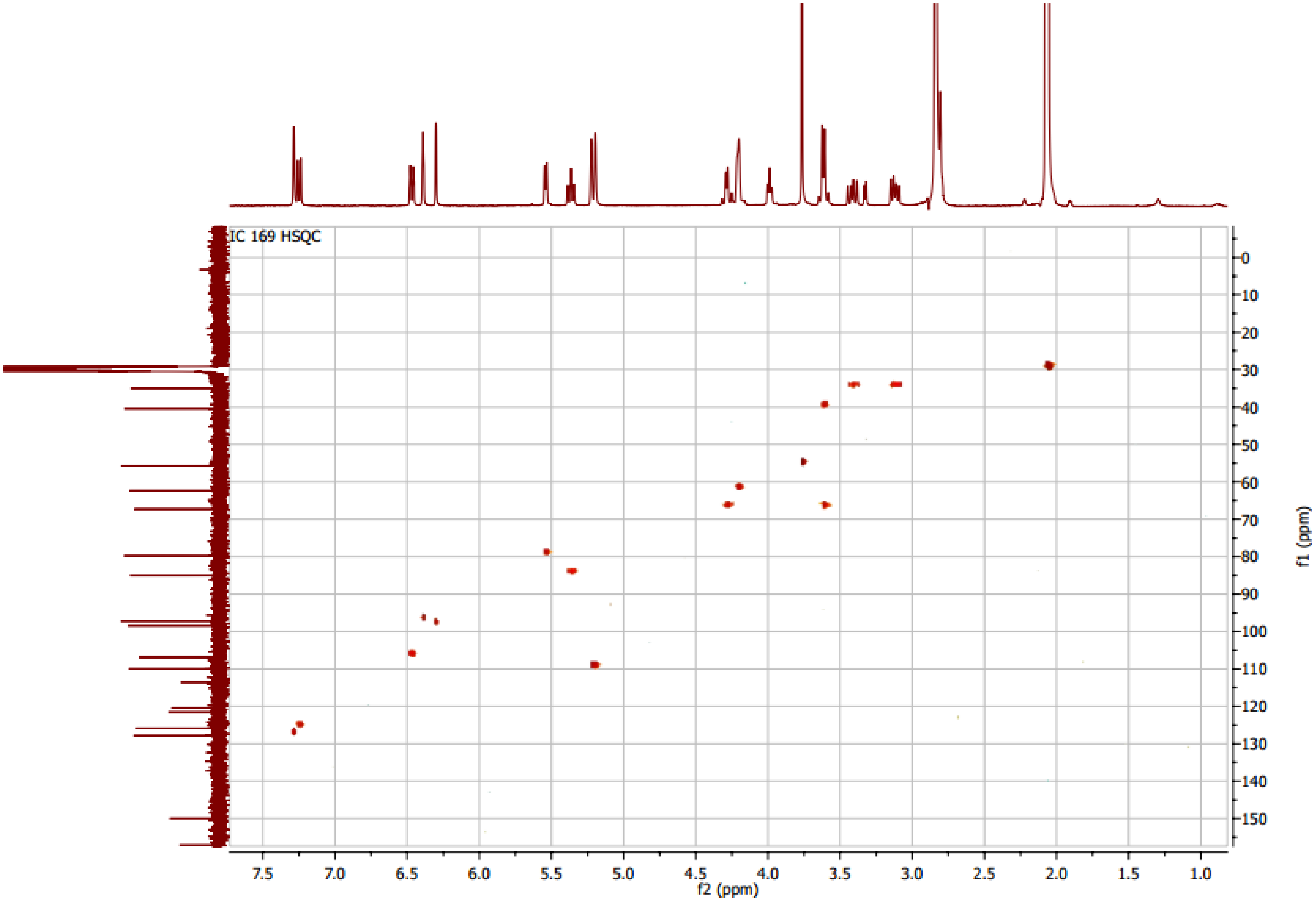

Full HSQC spectrum of compound 1

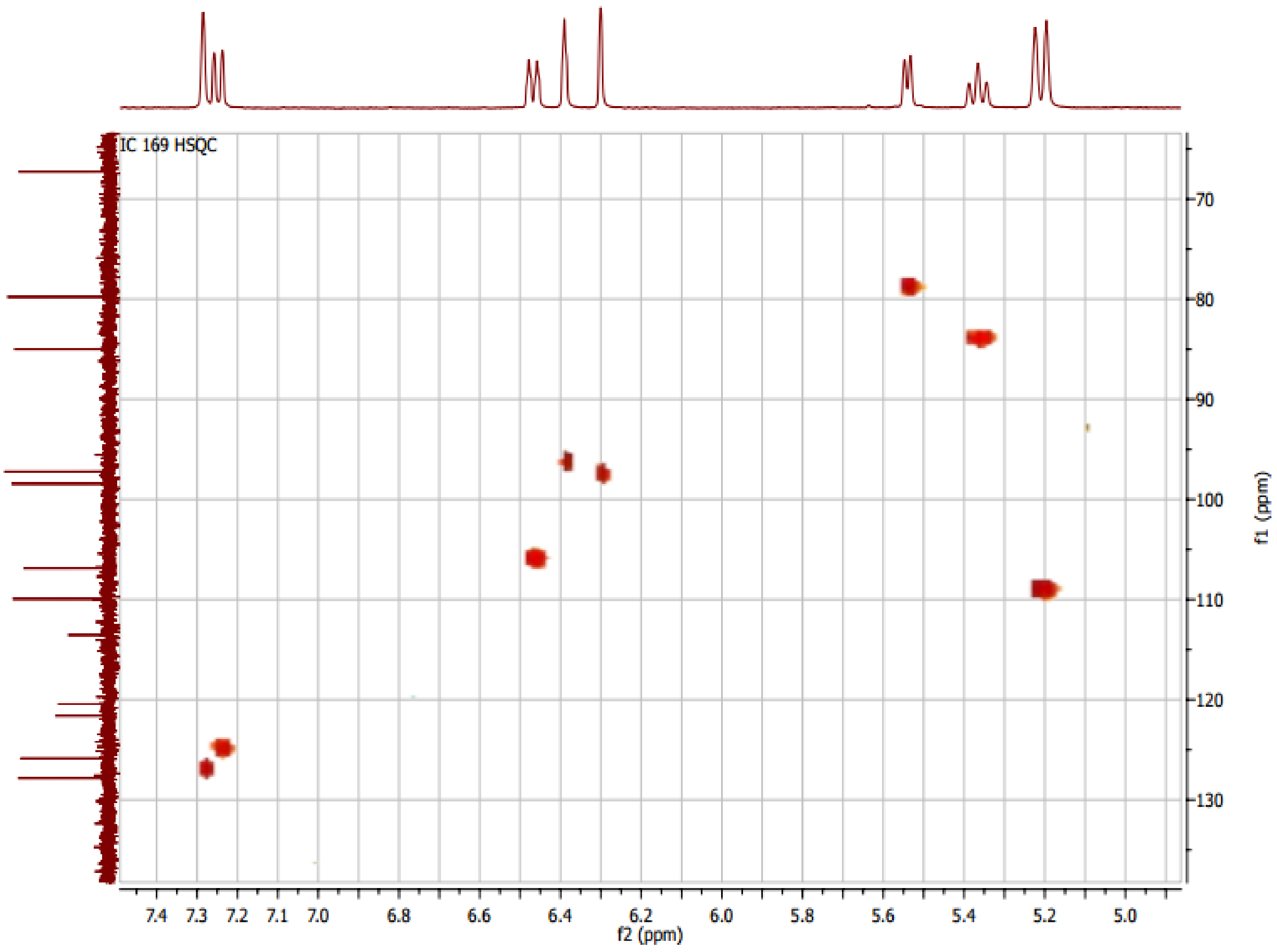

HSQC spectrum of compound 1 continued

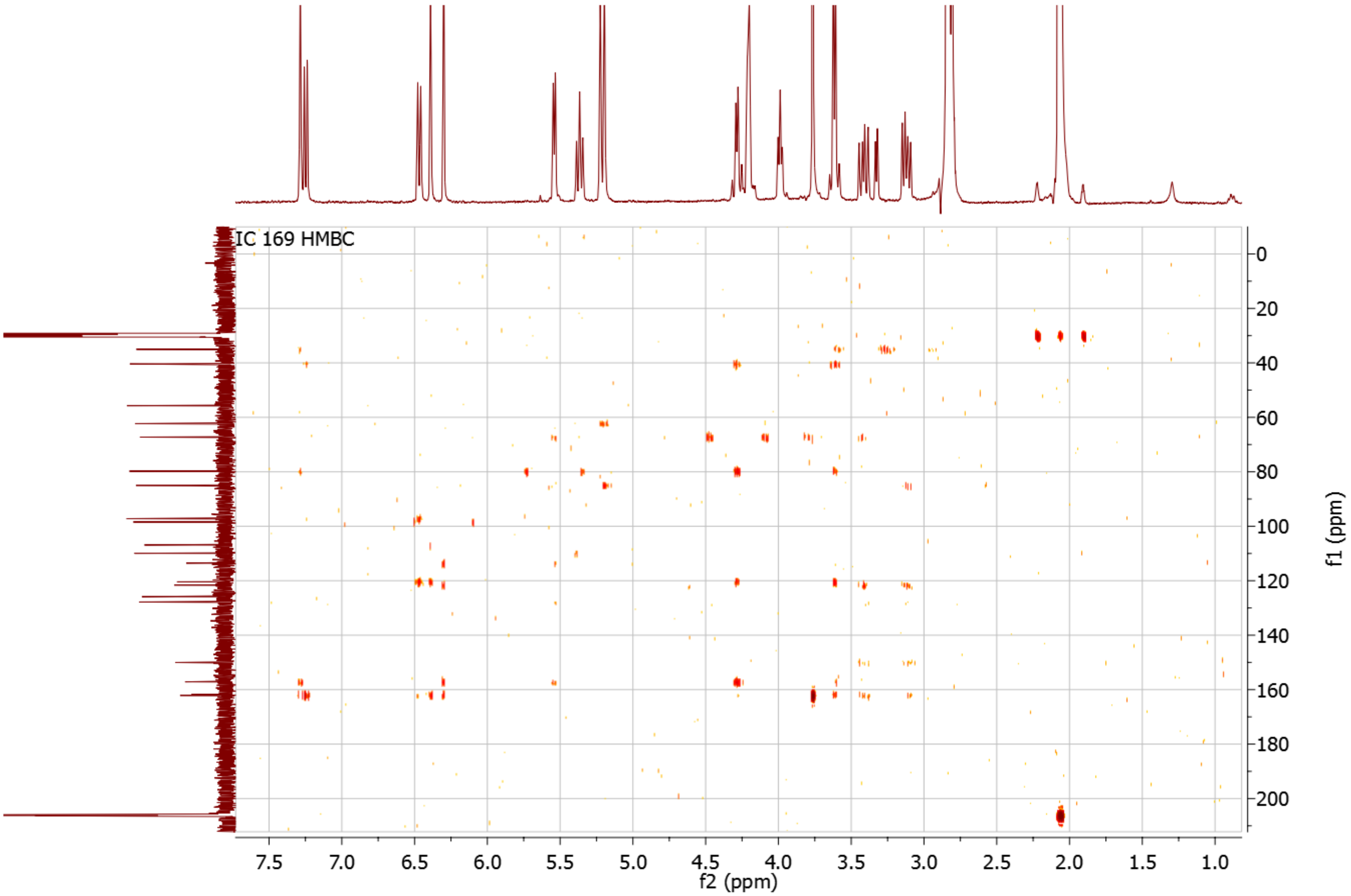

Full HMBC spectrum of compound 1

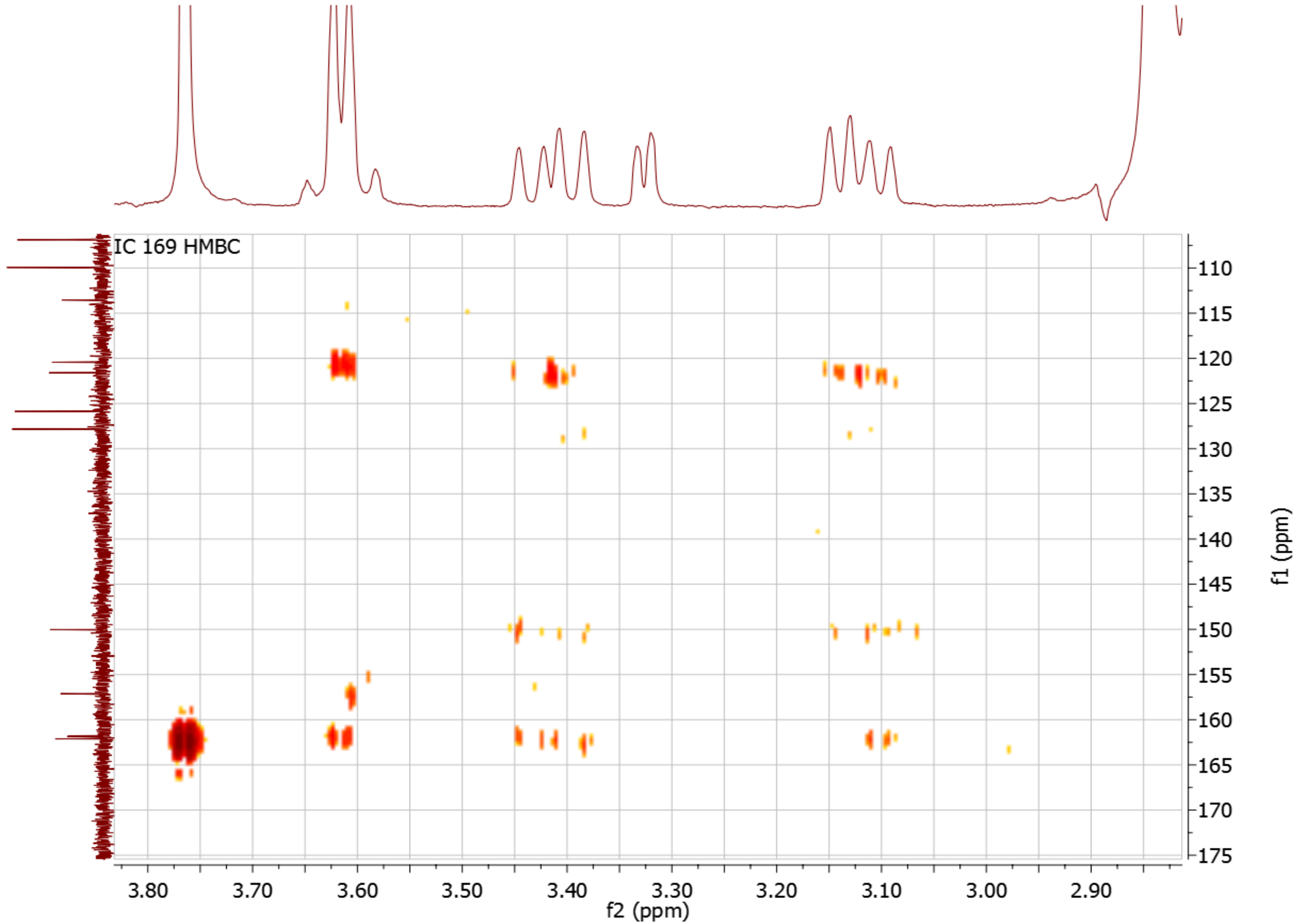

HMBC spectrum of compound 1 (aliphatic region)

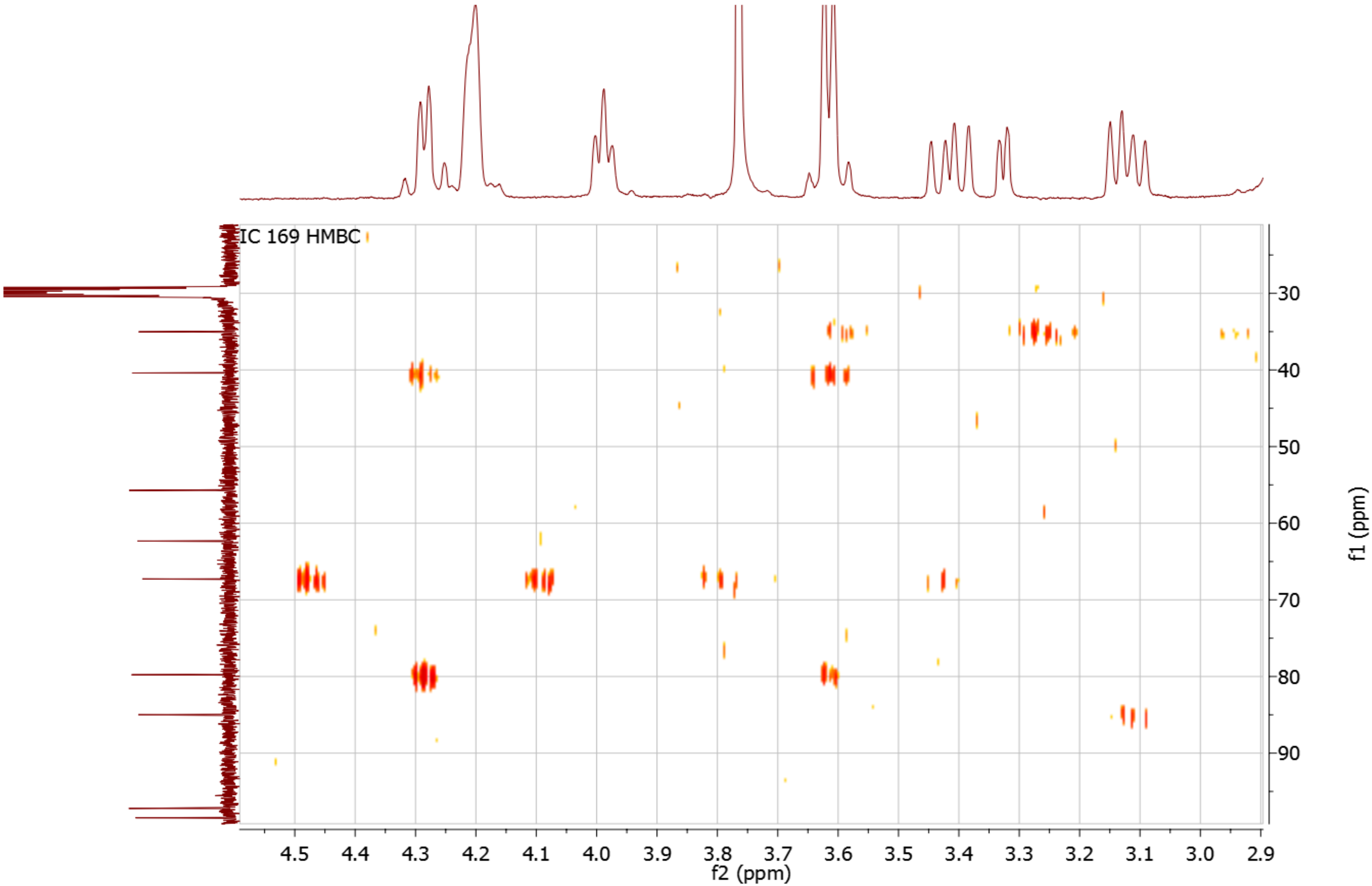

HMBC spectrum of compound 1 (aliphatic region) continued

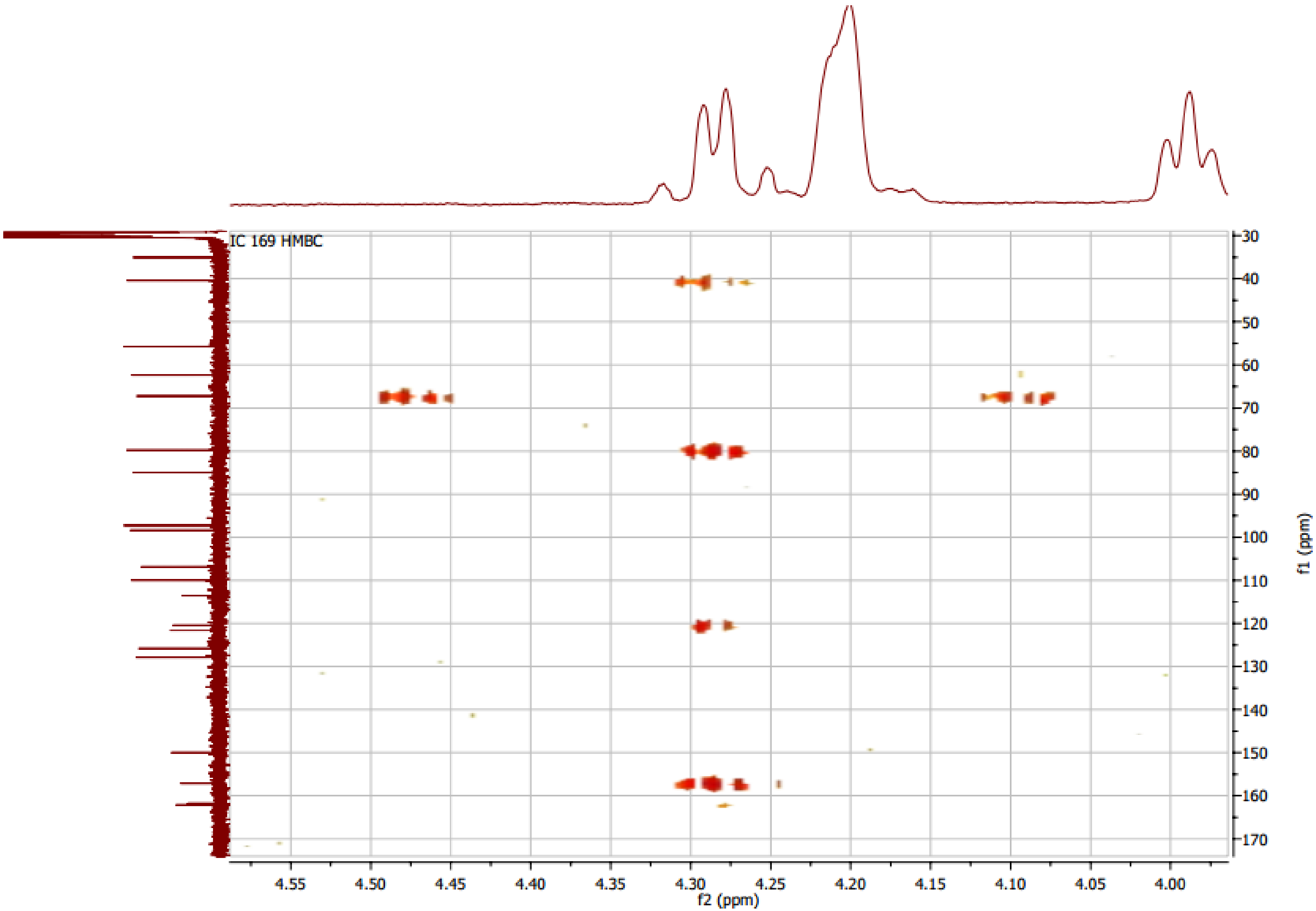

HMBC spectrum of compound 1 (aliphatic region) continued

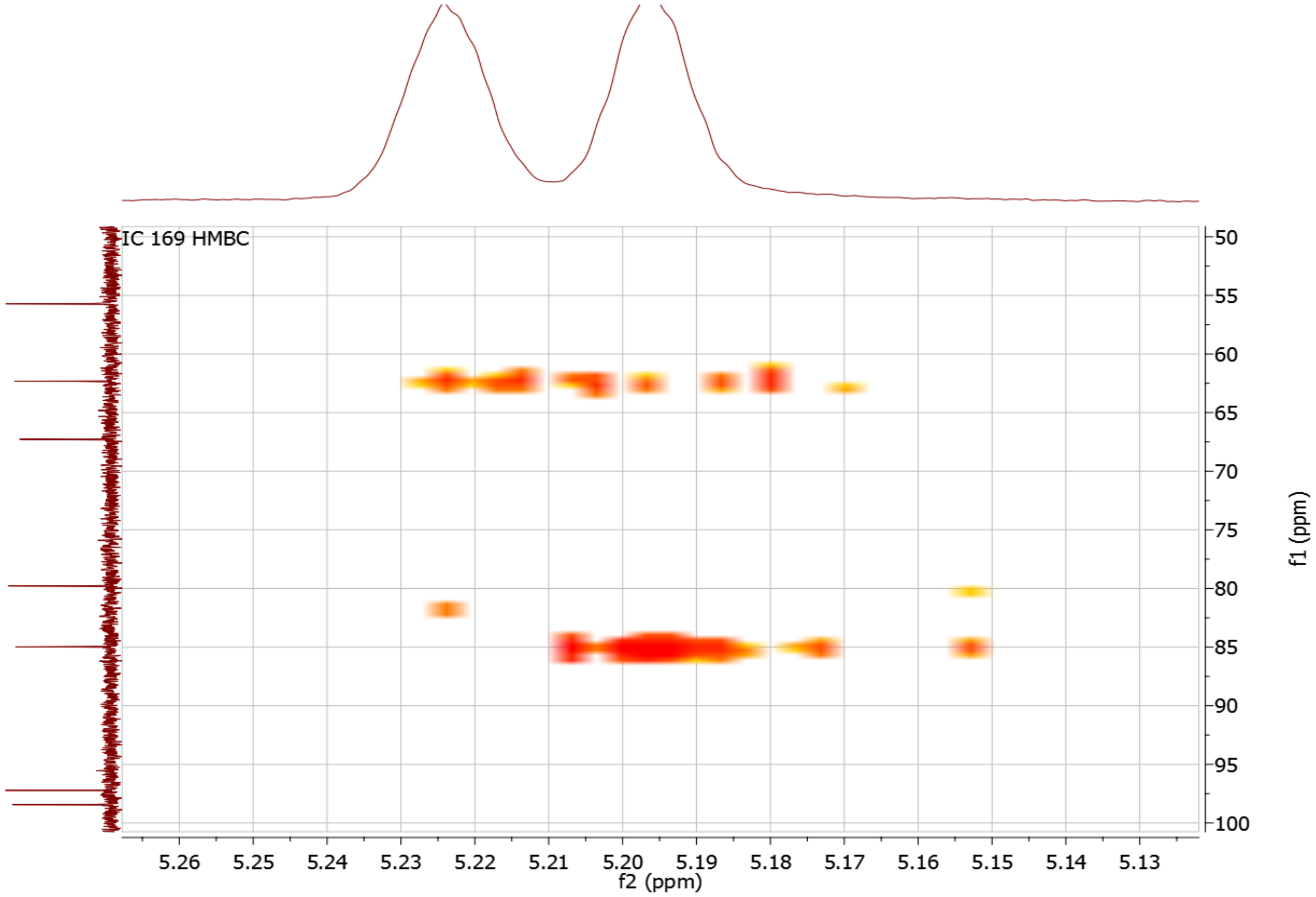

HMBC spectrum of compound 1 (correlations of H-15,15’ with C-13 and C-16)

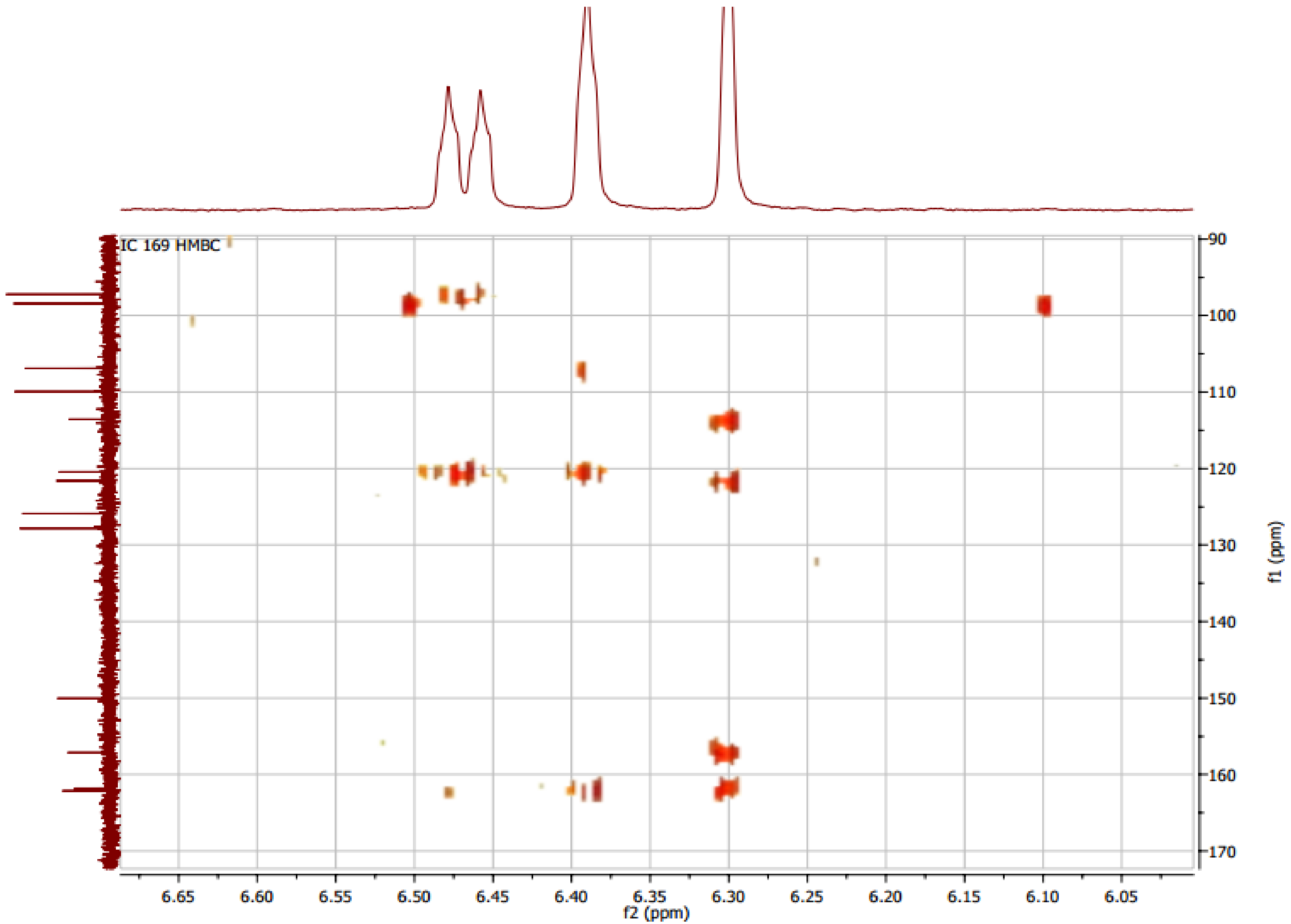

HMBC spectrum of compound 1 (aromatic region)

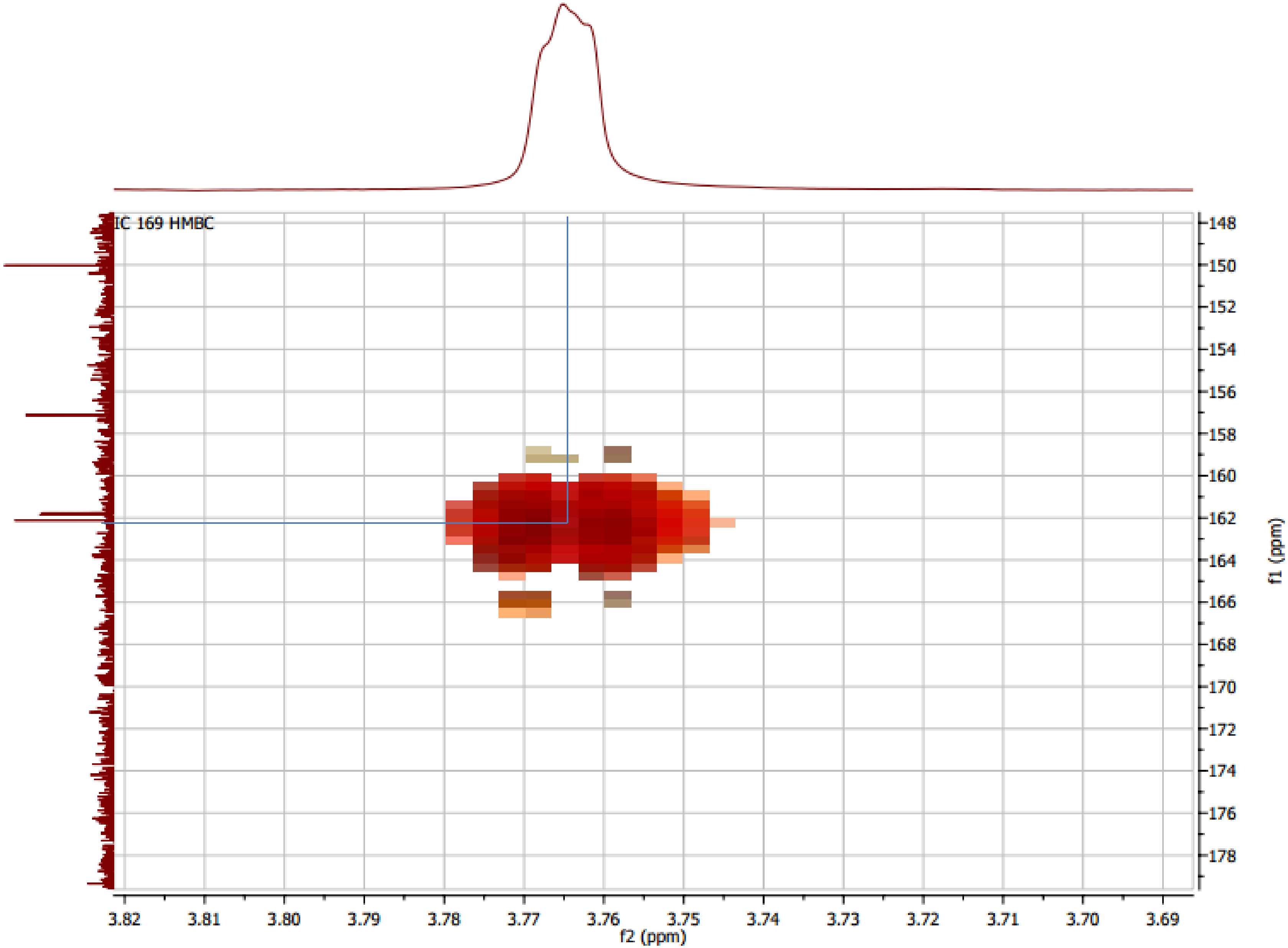

HMBC correlation of the methoxyl (δ 3.77) group with C-9 (δ 162.1)
