# Supplementary material for: Two new pterocarpans and a new pyrone derivative with cytotoxic activities from Ptycholobium contortum (N.E.Br.) Brummitt (Leguminosae): revised NMR assignment of mundulea lactone
Source: Chem Cent J. 2016 Oct 5;10:58. doi: 10.1186/s13065-016-0204-x (PMC5050614; doi:10.1186/s13065-016-0204-x)
Supplement: Supplementary file 2 — 10.1186/s13065-016-0204-x 1H and 13C NMR spectra of seputhecarpan D. 2. [file 13065_2016_204_MOESM2_ESM.pdf]

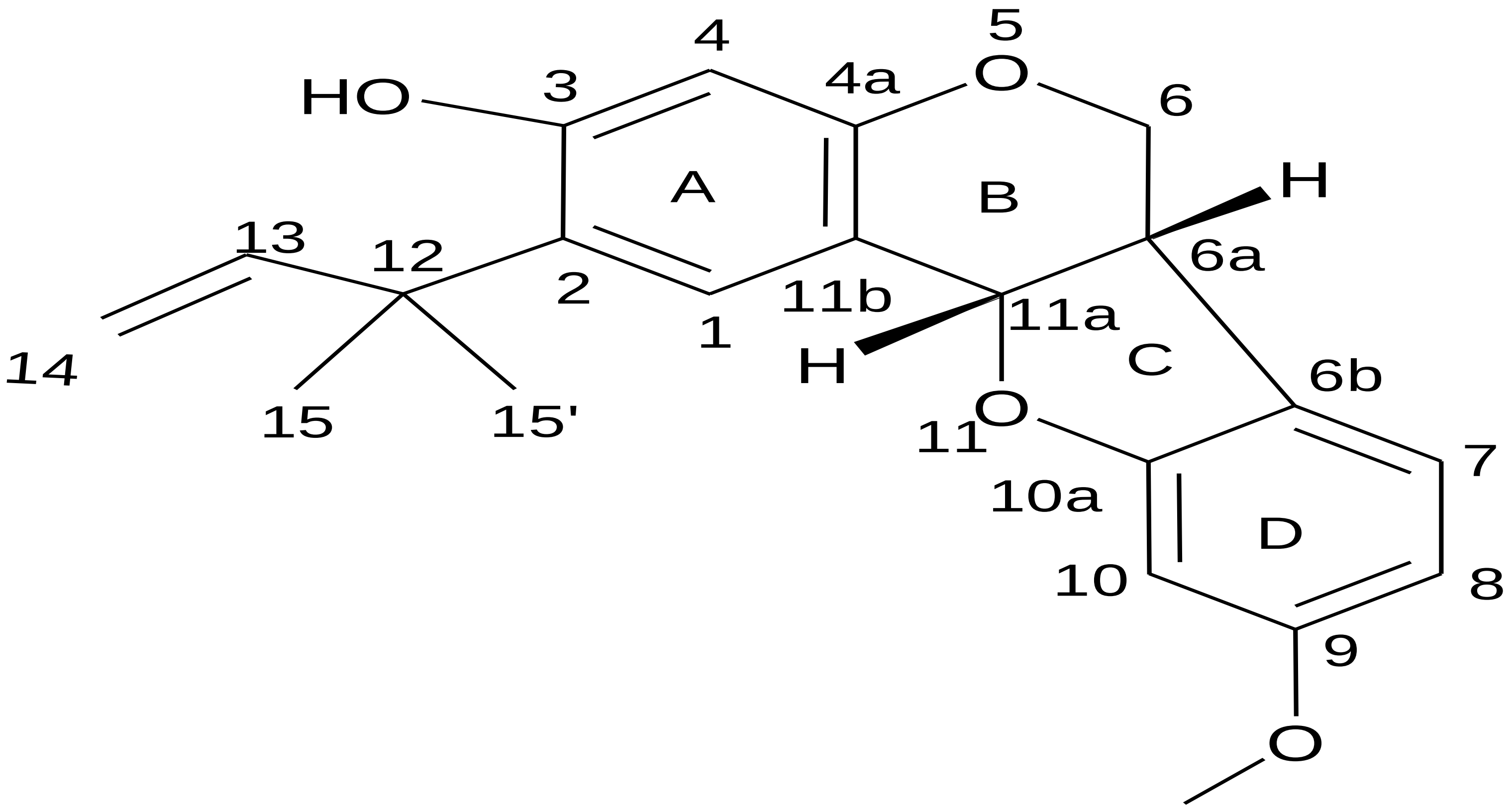

2

Seputhecarpan D

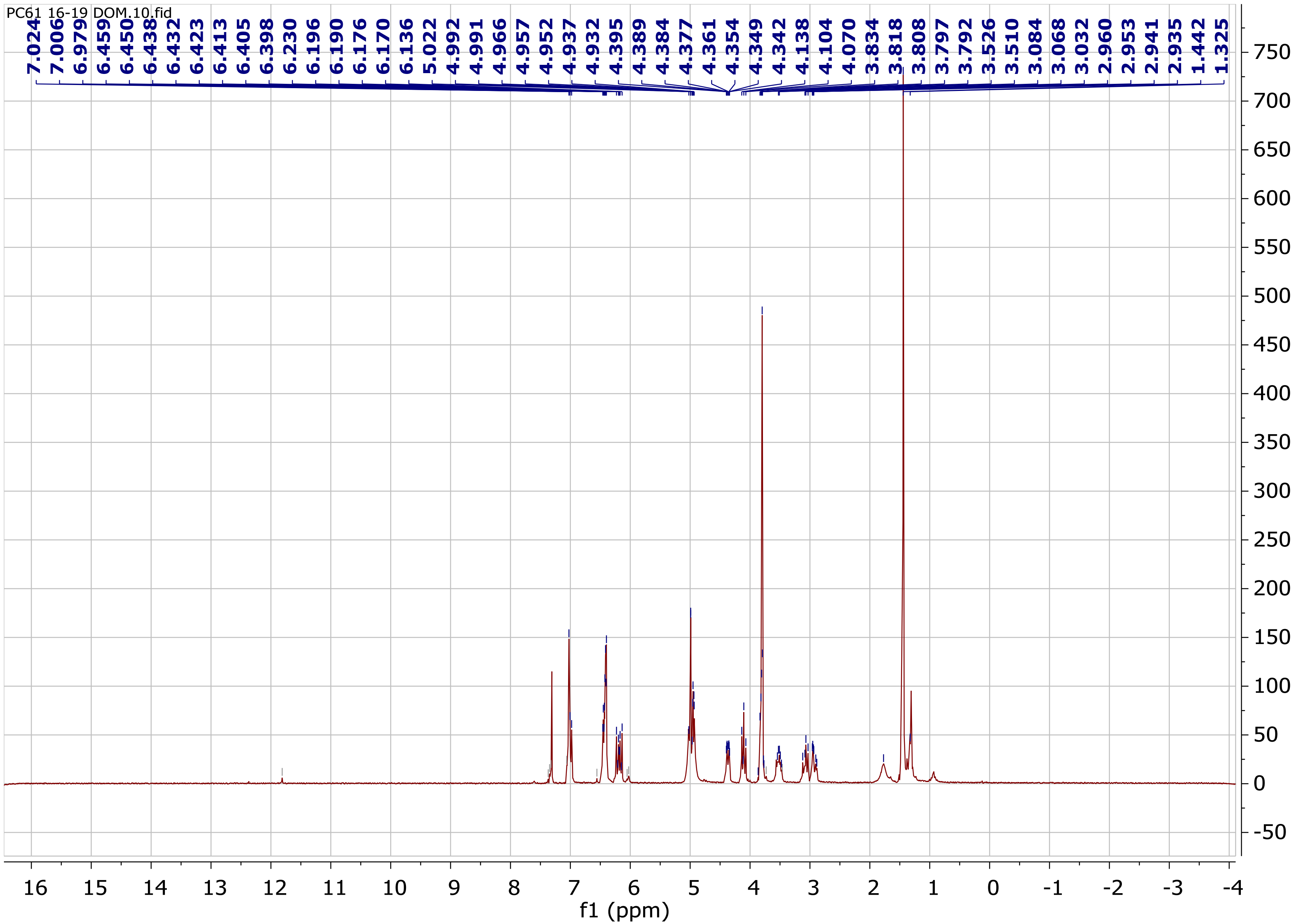

PC61 16-19 DOM.10.fid
7.024 7.006 6.979 6.459 6.450 6.438 6.432 6.423 6.413 6.405 6.398 6.230 6.196 6.190 6.176 6.170 6.136 5.022 4.992 4.991 4.966 4.957 4.952 4.937 4.932 4.395 4.389 4.384 4.377 4.361 4.354 4.349 4.342 4.138 4.104 4.070 3.834 3.818 3.808 3.797 3.792 3.526 3.510 3.084 3.068 3.032 2.960 2.953 2.941 2.935 1.442 1.325
f1 (ppm)

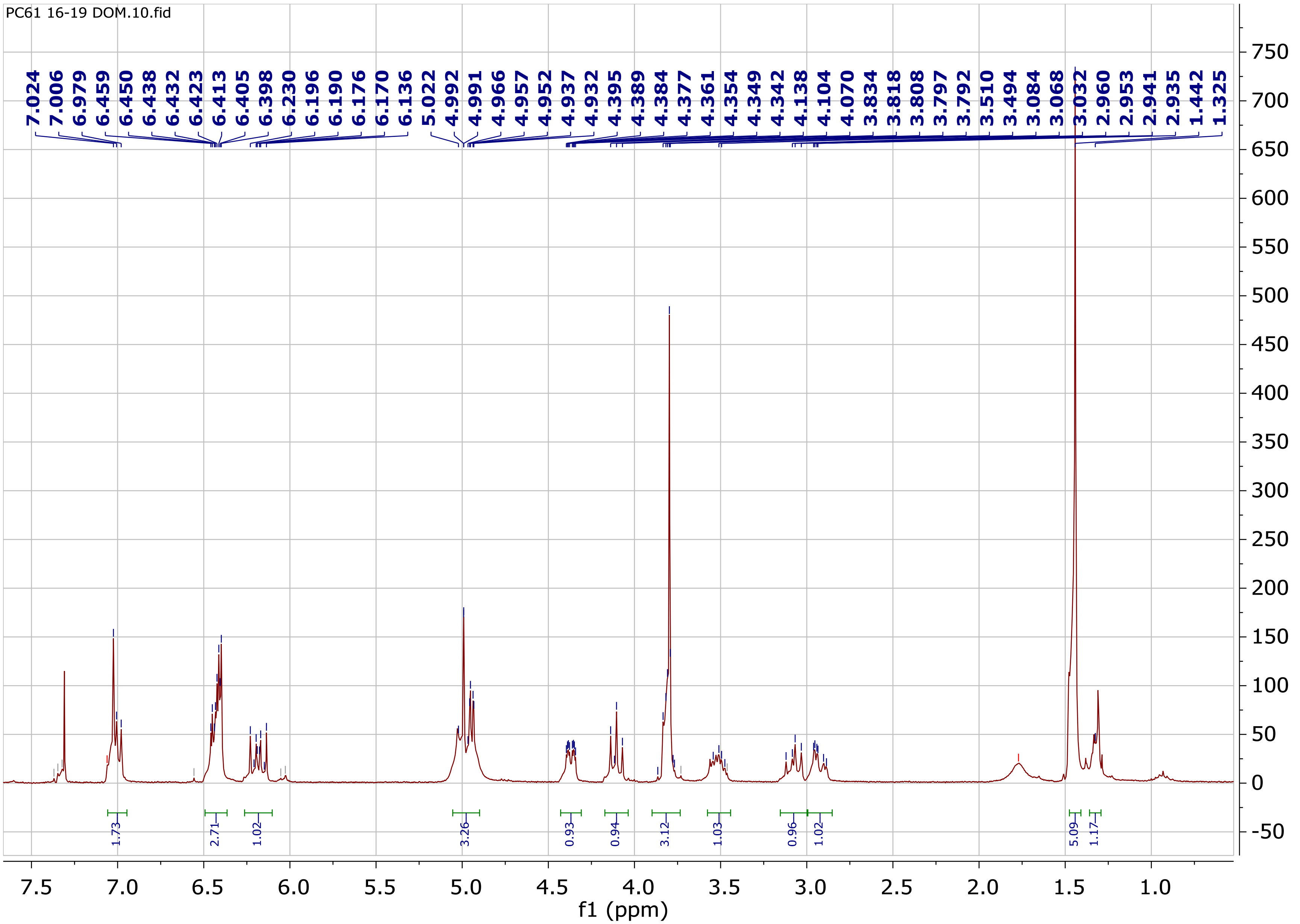
PC61 16-19 DOM.10.fid
7.024 7.006 6.979 6.459 6.450 6.438 6.432 6.423 6.413 6.405 6.398 6.230 6.196 6.190 6.176 6.170 6.136 5.022 4.992 4.991 4.966 4.957 4.952 4.937 4.932 4.395 4.389 4.384 4.377 4.361 4.354 4.349 4.342 4.138 4.104 4.070 3.834 3.818 3.808 3.797 3.792 3.510 3.494 3.084 3.068 3.032 2.960 2.953 2.941 2.935 1.442 1.325
1.73 2.71 1.02 3.26 0.93 0.94 3.12 1.03 0.96 1.02 5.09 1.17
f1 (ppm)

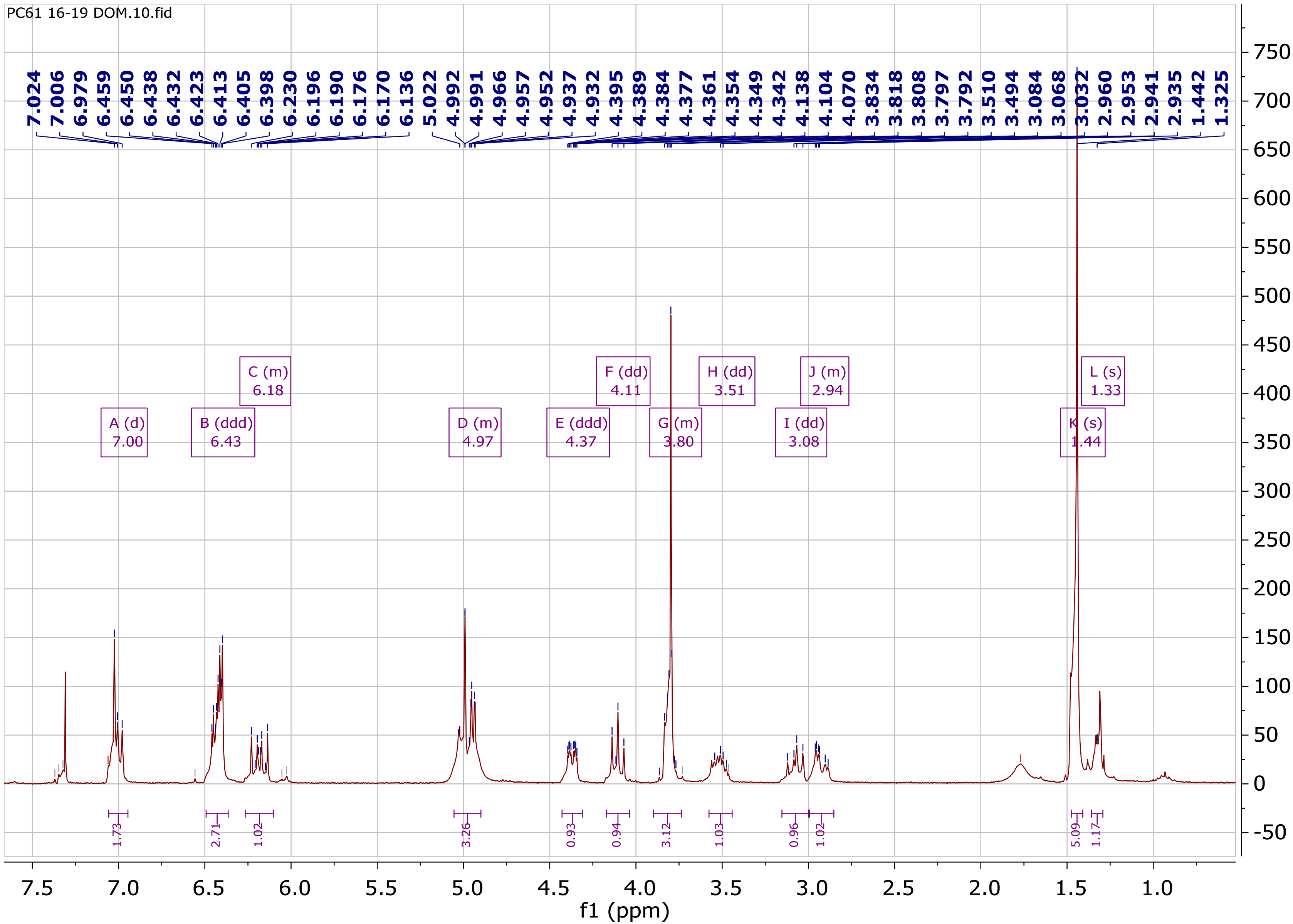
PC61 16-19 DOM.10.fid
7.024 7.006 6.979 6.459 6.450 6.438 6.432 6.423 6.413 6.405 6.398 6.230 6.196 6.190 6.176 6.170 6.136 5.022 4.992 4.991 4.966 4.957 4.952 4.937 4.932 4.395 4.389 4.384 4.377 4.361 4.354 4.349 4.342 4.138 4.104 4.070 3.834 3.818 3.808 3.797 3.792 3.510 3.494 3.084 3.068 3.032 2.960 2.953 2.941 2.935 1.442 1.325
A (d) 7.00
B (ddd) 6.43
C (m) 6.18
D (m) 4.97
E (ddd) 4.37
F (dd) 4.11
G (m) 3.80
H (dd) 3.51
I (dd) 3.08
J (m) 2.94
K (s) 1.44
L (s) 1.33
1.73 2.71 1.02 3.26 0.93 0.94 3.12 1.03 0.96 1.02 5.09 1.17
f1 (ppm)

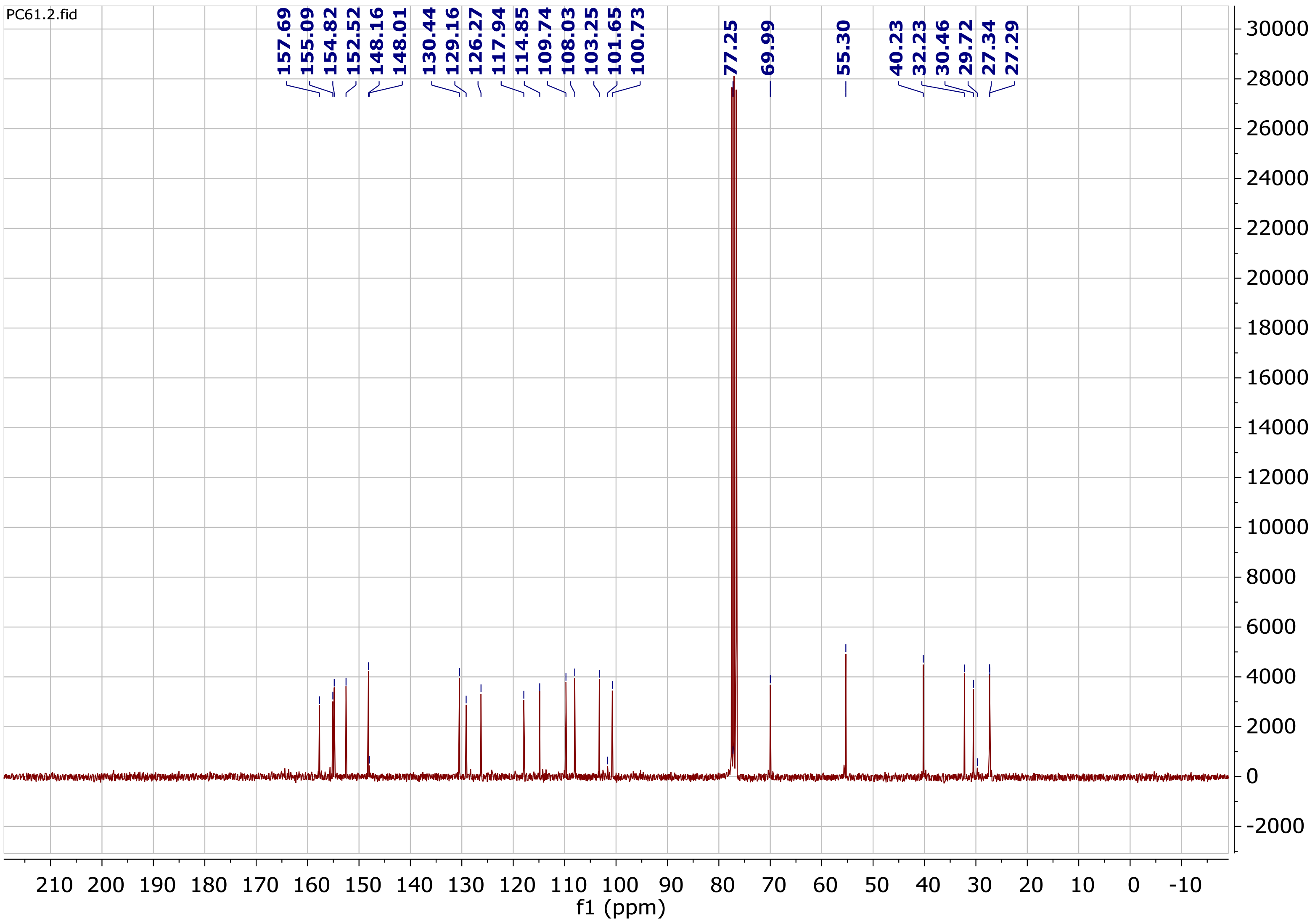

PC61.2.fid
157.69
155.09
154.82
152.52
148.16
148.01
130.44
129.16
126.27
117.94
114.85
109.74
108.03
103.25
101.65
100.73
77.25
69.99
55.30
40.23
32.23
30.46
29.72
27.34
27.29
f1 (ppm)

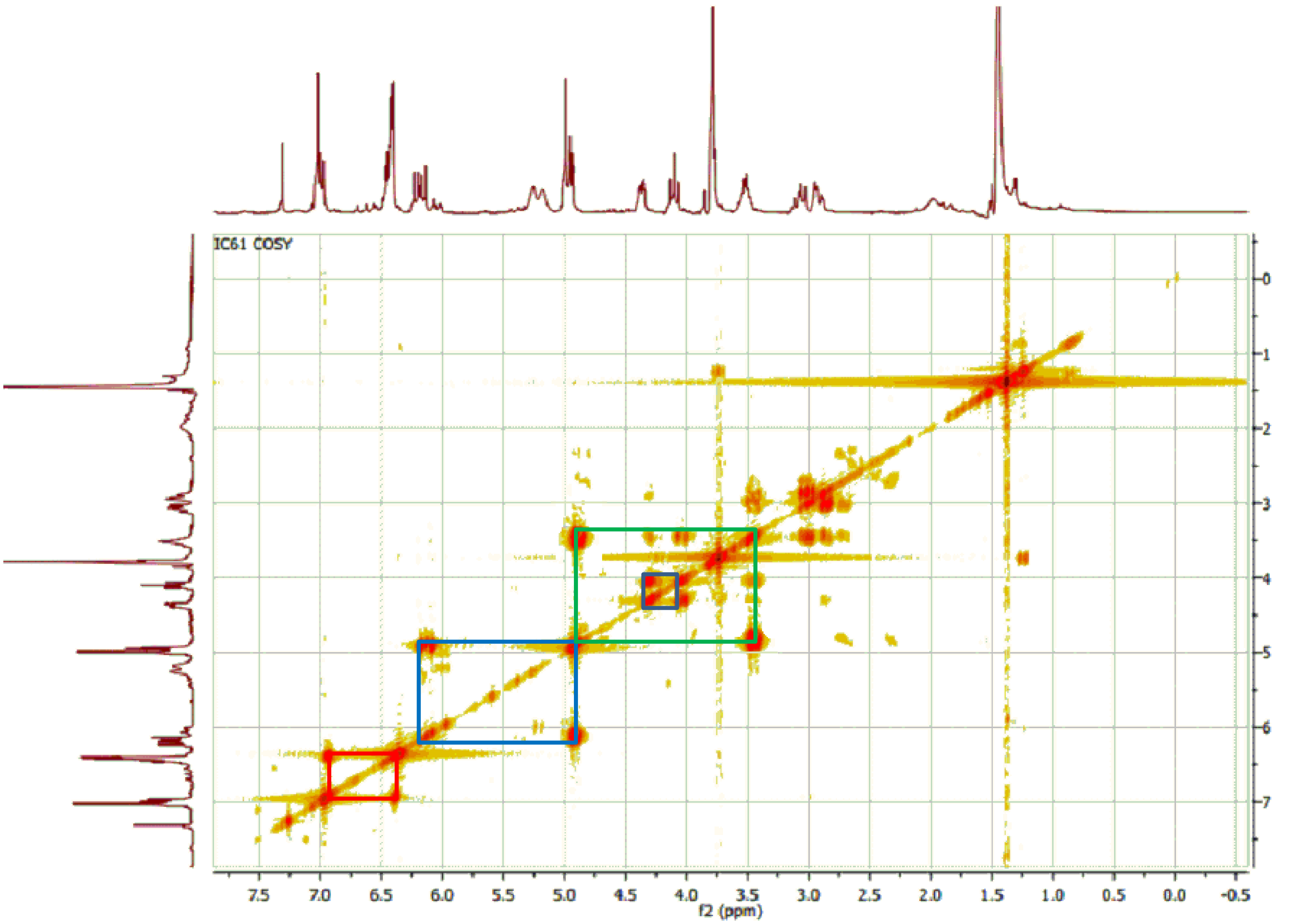

COSY spectrum of compound 2

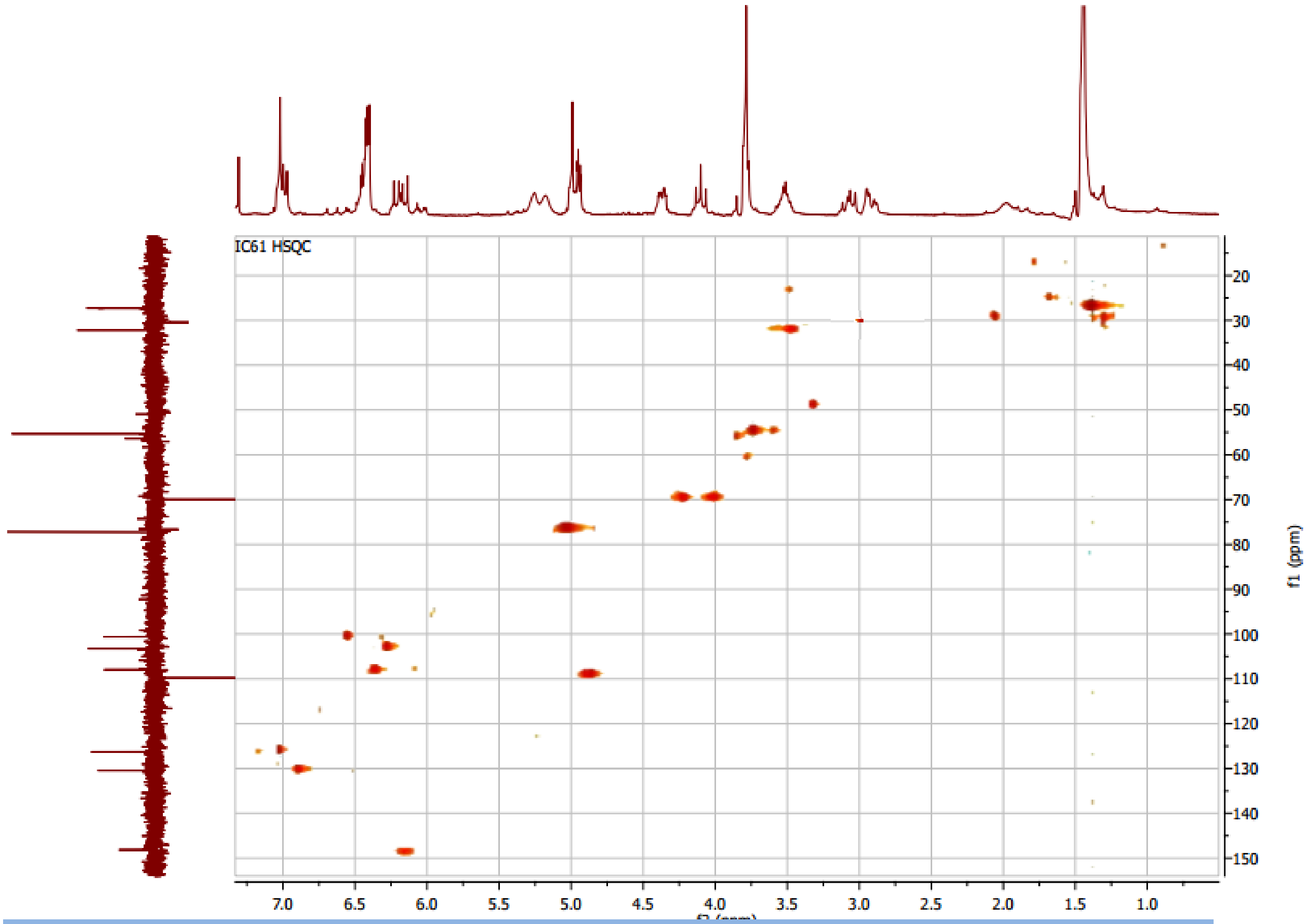

HSQC spectrum of compound 2

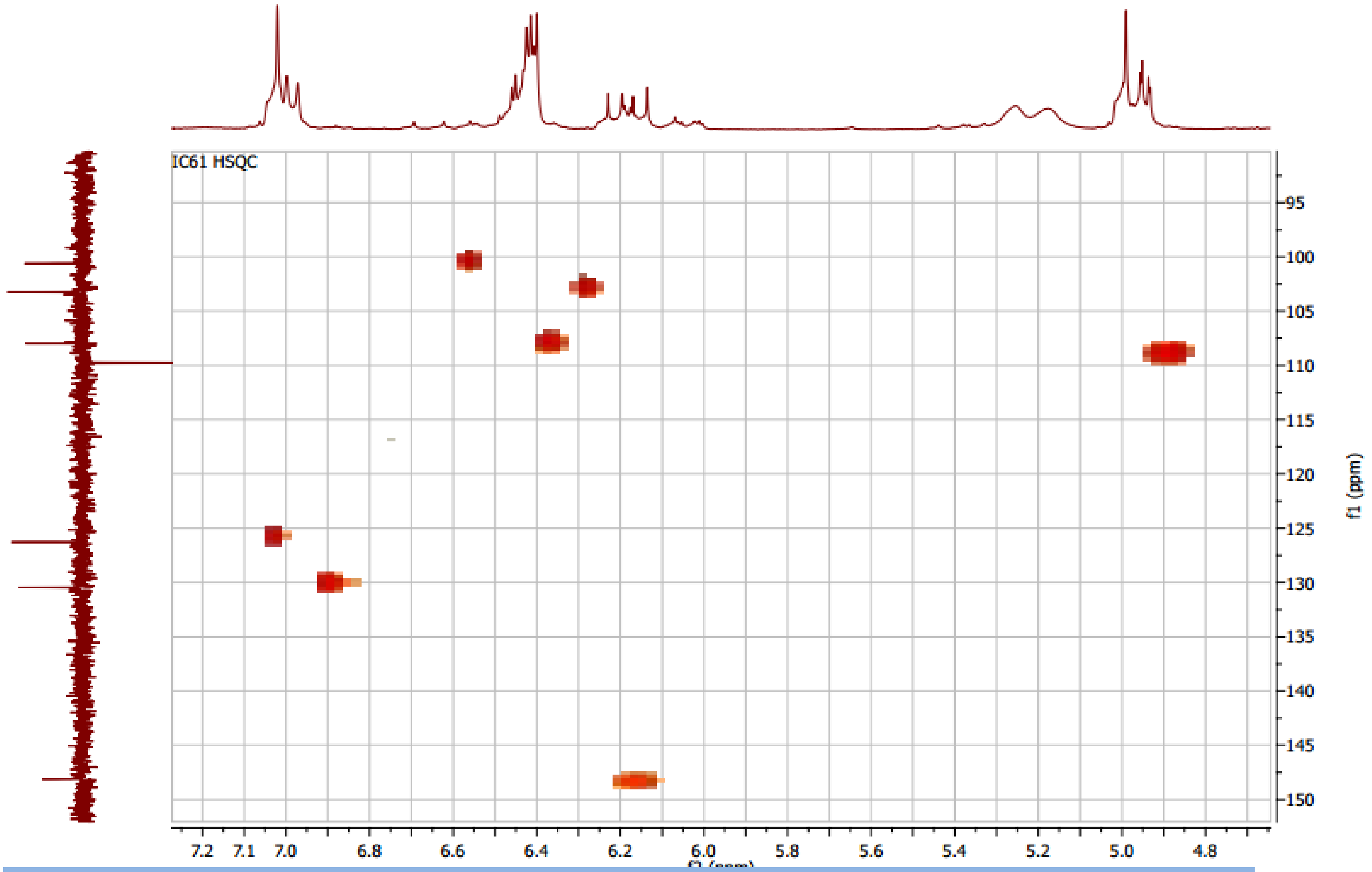

HSQC spectrum of compound 2 continued

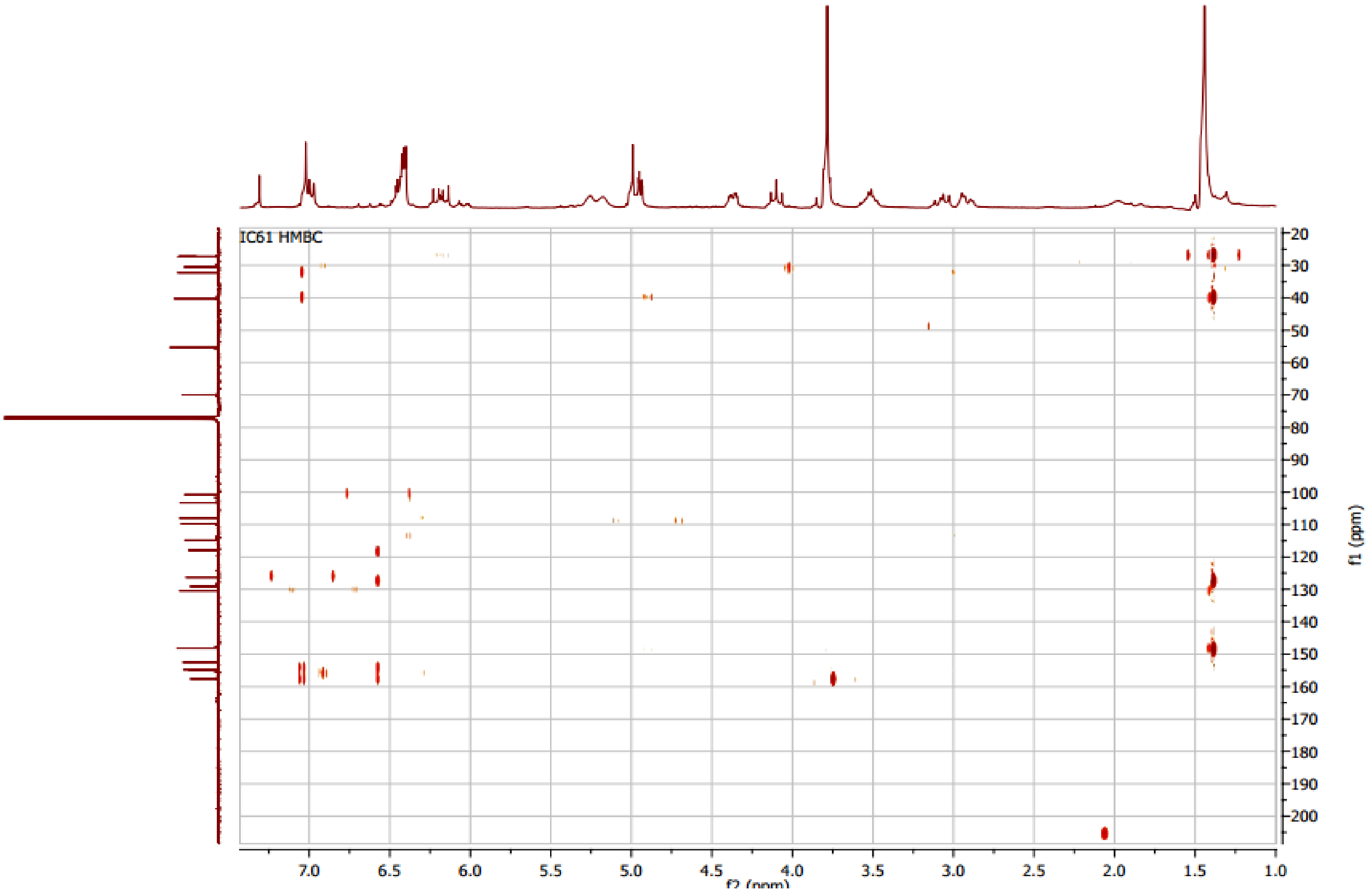

Full HMBC spectrum of compound 2

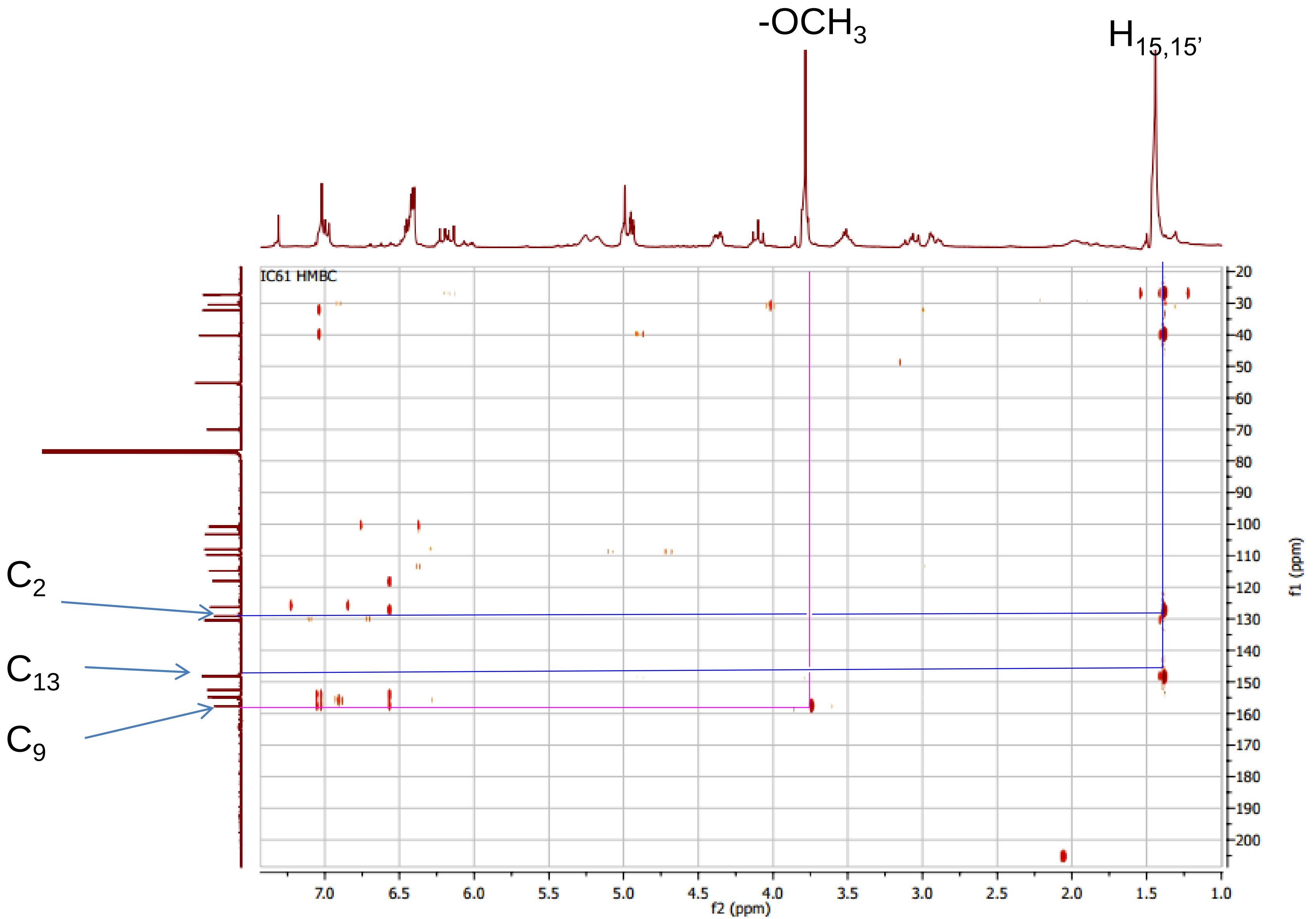

Important HMBC correlations of compound 2

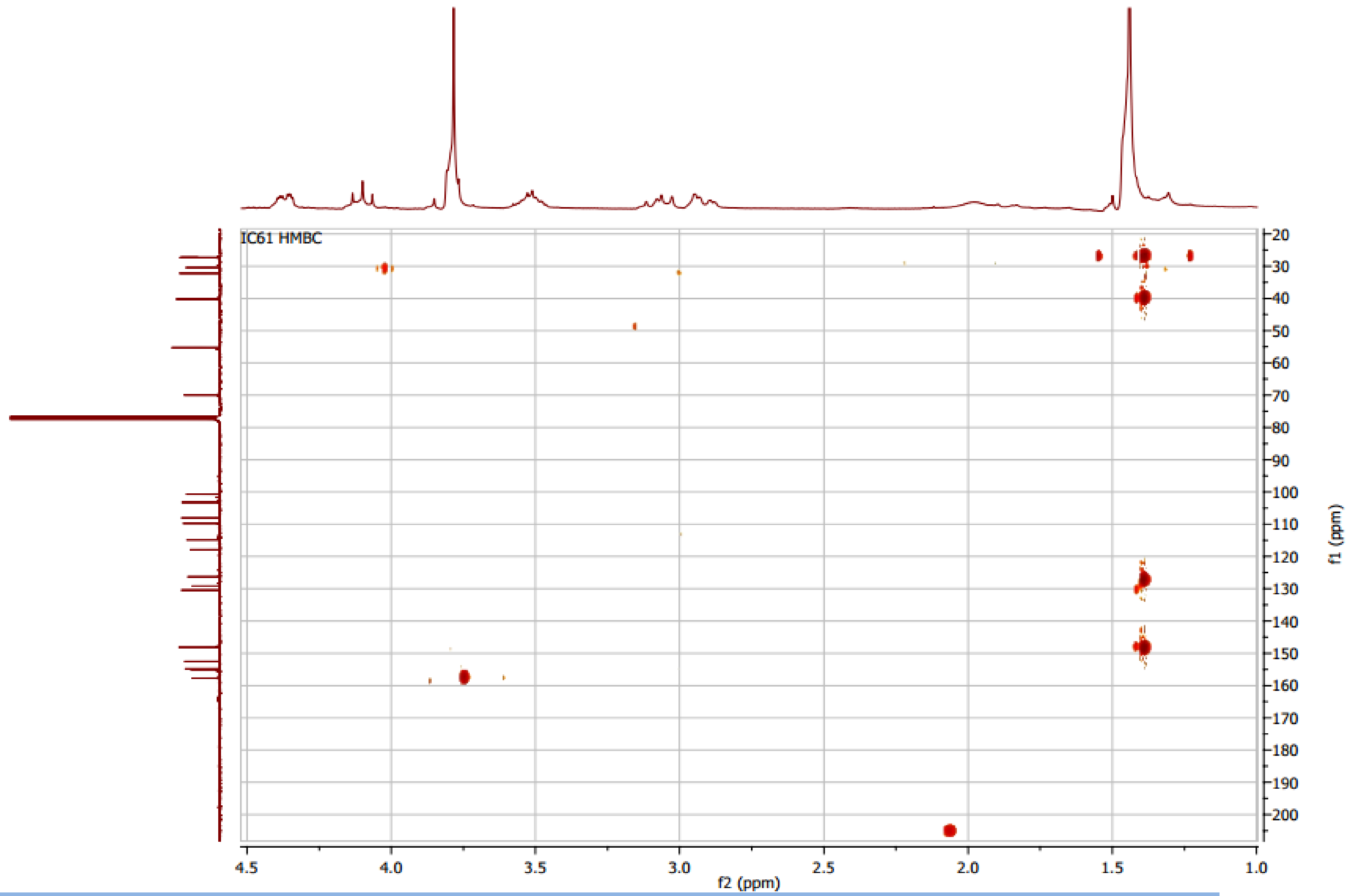

HMBC spectrum of compound 2 (methoxy and methyl groups)

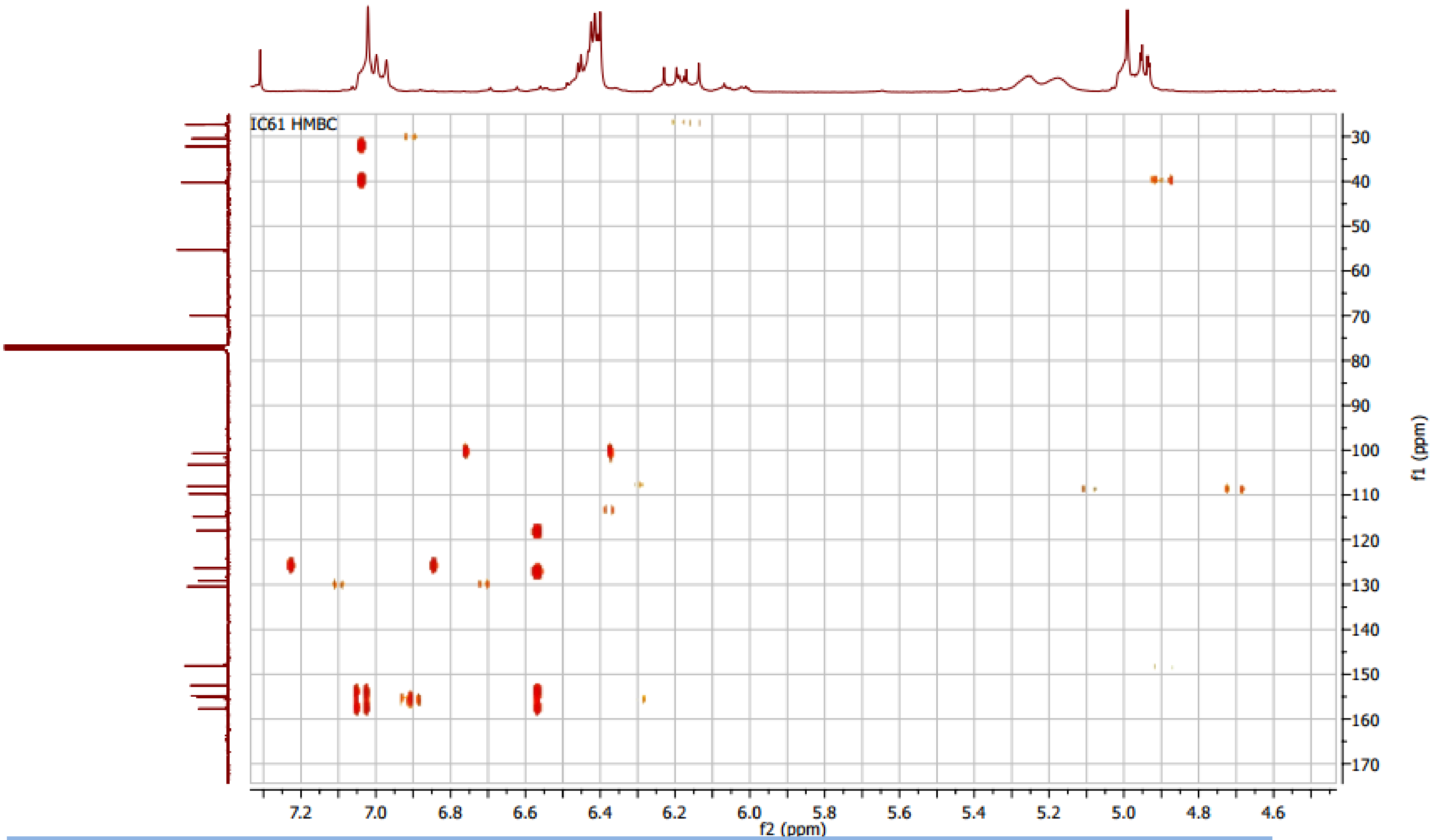

HMBC spectrum of compound 2 (aromatic region)
