# Supplementary material for: Two new pterocarpans and a new pyrone derivative with cytotoxic activities from Ptycholobium contortum (N.E.Br.) Brummitt (Leguminosae): revised NMR assignment of mundulea lactone
Source: Chem Cent J. 2016 Oct 5;10:58. doi: 10.1186/s13065-016-0204-x (PMC5050614; doi:10.1186/s13065-016-0204-x)
Supplement: Supplementary file 3 — 10.1186/s13065-016-0204-x 1H and 13C NMR spectra of pythylopyrone A 3 showing the signals of the additional γ,γ-dimethylallyle group in position 4 of the molecule. [file 13065_2016_204_MOESM3_ESM.pdf]

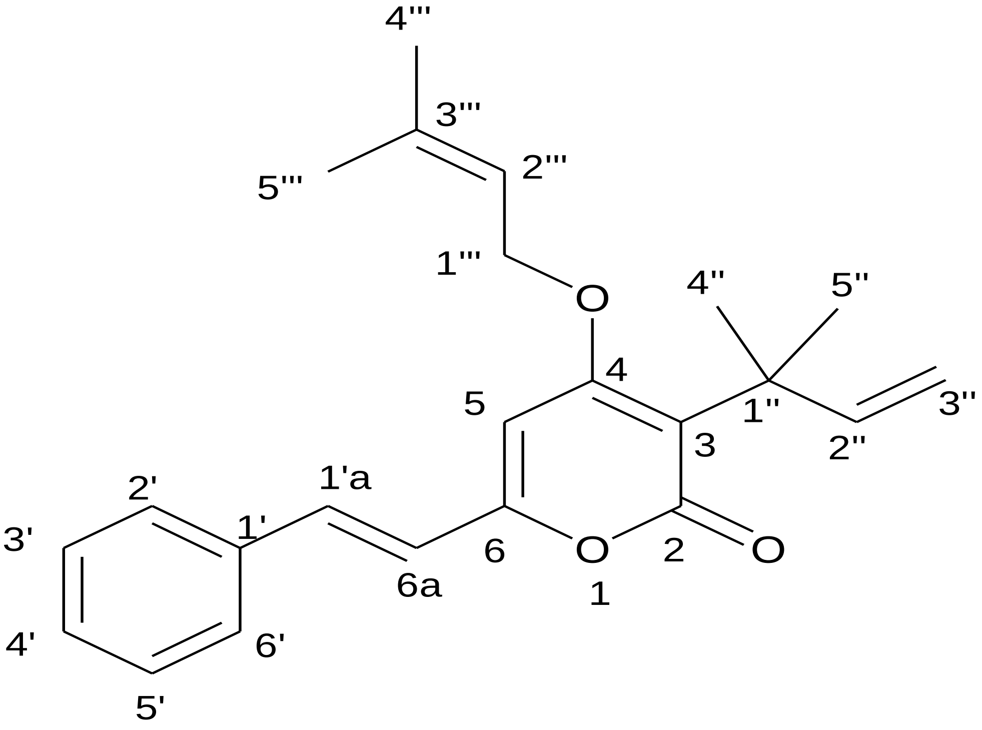


**3**

**Ptycholopyrone A**

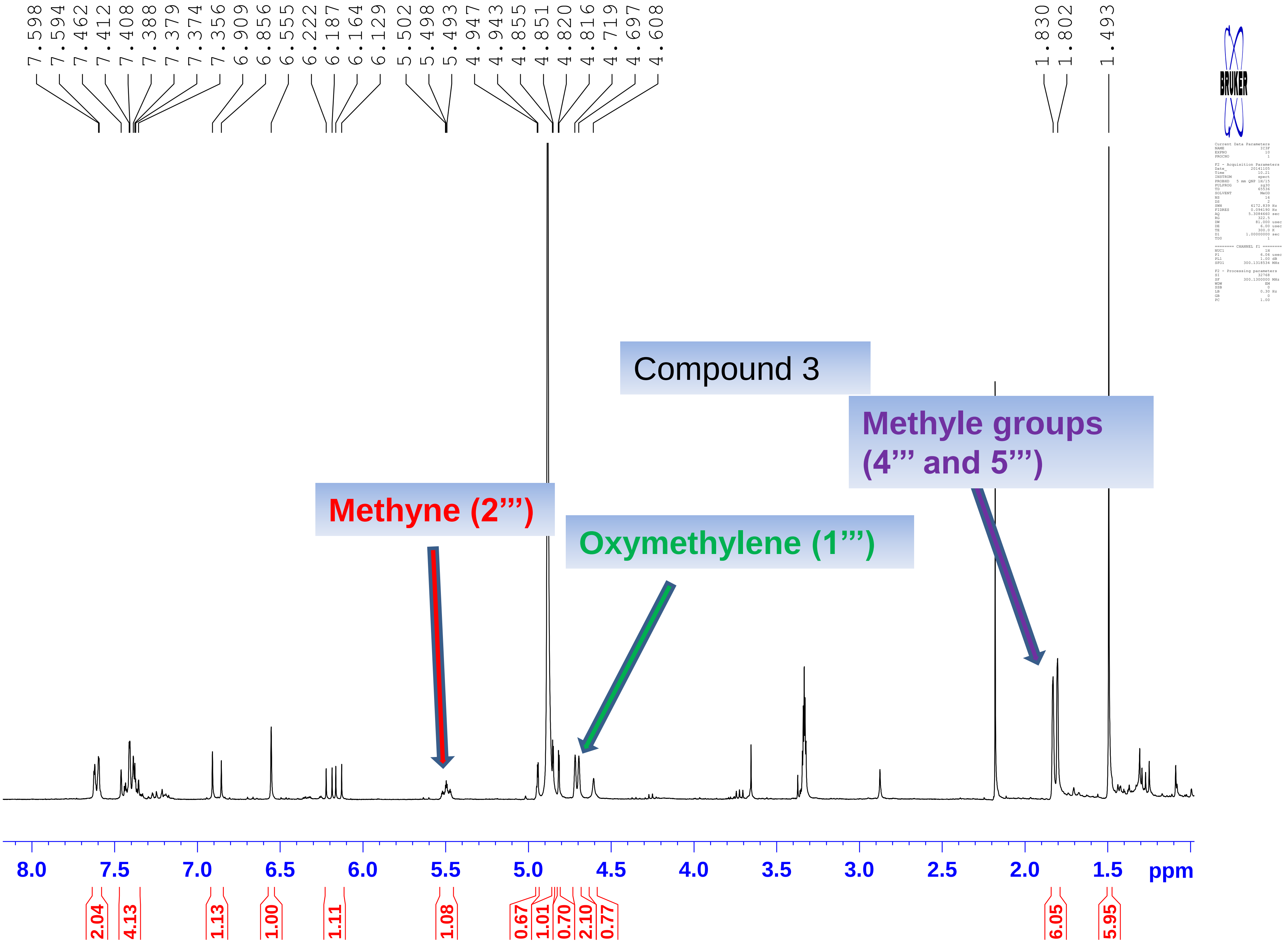

7.598
7.594
7.462
7.412
7.408
7.388
7.379
7.374
7.356
6.909
6.856
6.555
6.222
6.187
6.164
6.129
5.502
5.498
5.493
4.947
4.943
4.855
4.851
4.820
4.816
4.719
4.697
4.608
1.830
1.802
1.493
BRUKER
Compound 3
Methyle groups (4''' and 5''')
Methyne (2''')
Oxymethylene (1''')
8.0
7.5
7.0
6.5
6.0
5.5
5.0
4.5
4.0
3.5
3.0
2.5
2.0
1.5
ppm
2.04
4.13
1.13
1.00
1.11
1.08
0.67
1.01
0.70
2.10
0.77
6.05
5.95

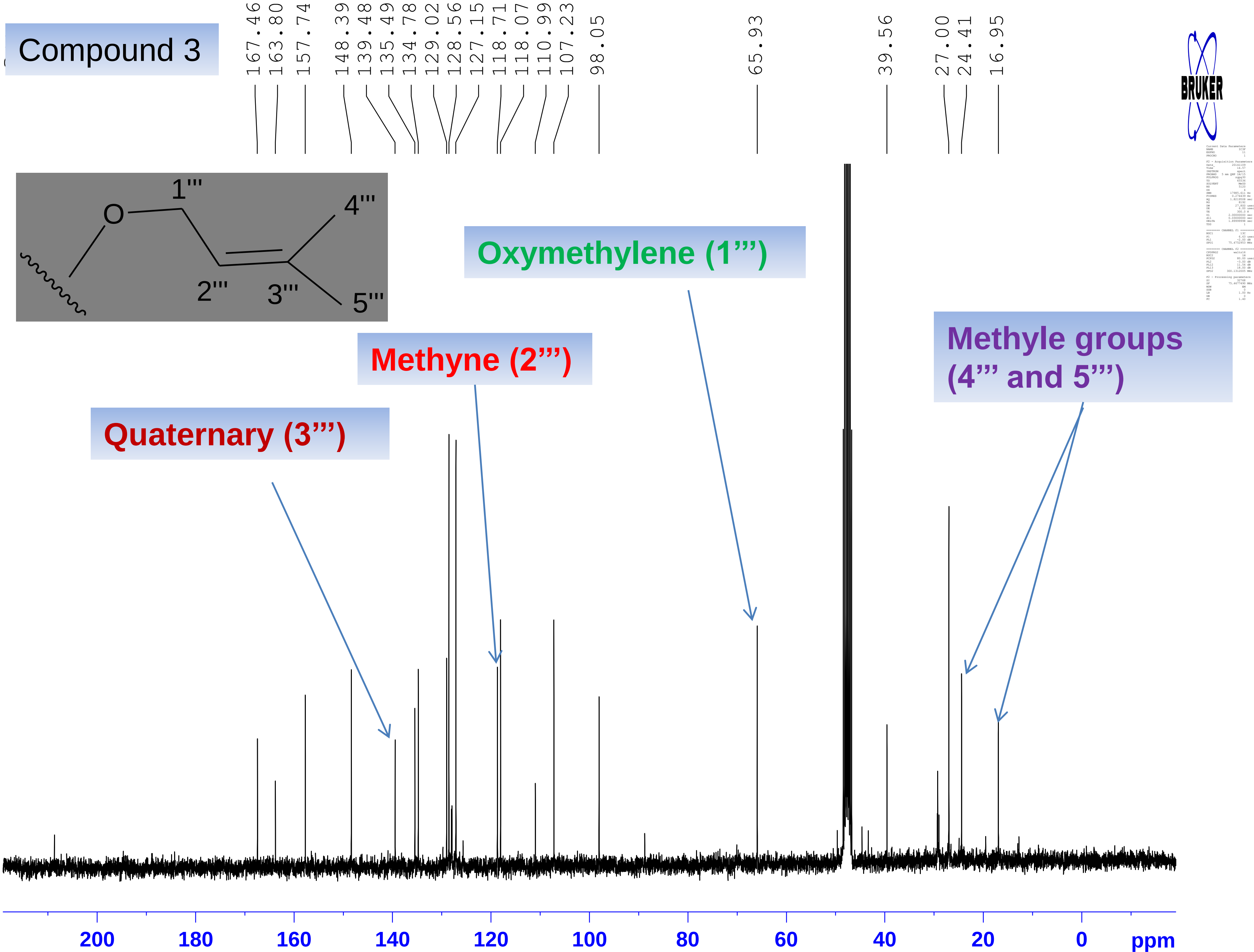
Compound 3
167.46
163.80
157.74
148.39
139.48
135.49
134.78
129.02
128.56
127.15
118.71
118.07
110.99
107.23
98.05
65.93
39.56
27.00
24.41
16.95
BRUKER
O
1'''
2'''
3'''
4'''
5'''
Oxymethylene (1''')
Methyne (2''')
Quaternary (3''')
Methyle groups (4''' and 5''')
200
180
160
140
120
100
80
60
40
20
0
ppm

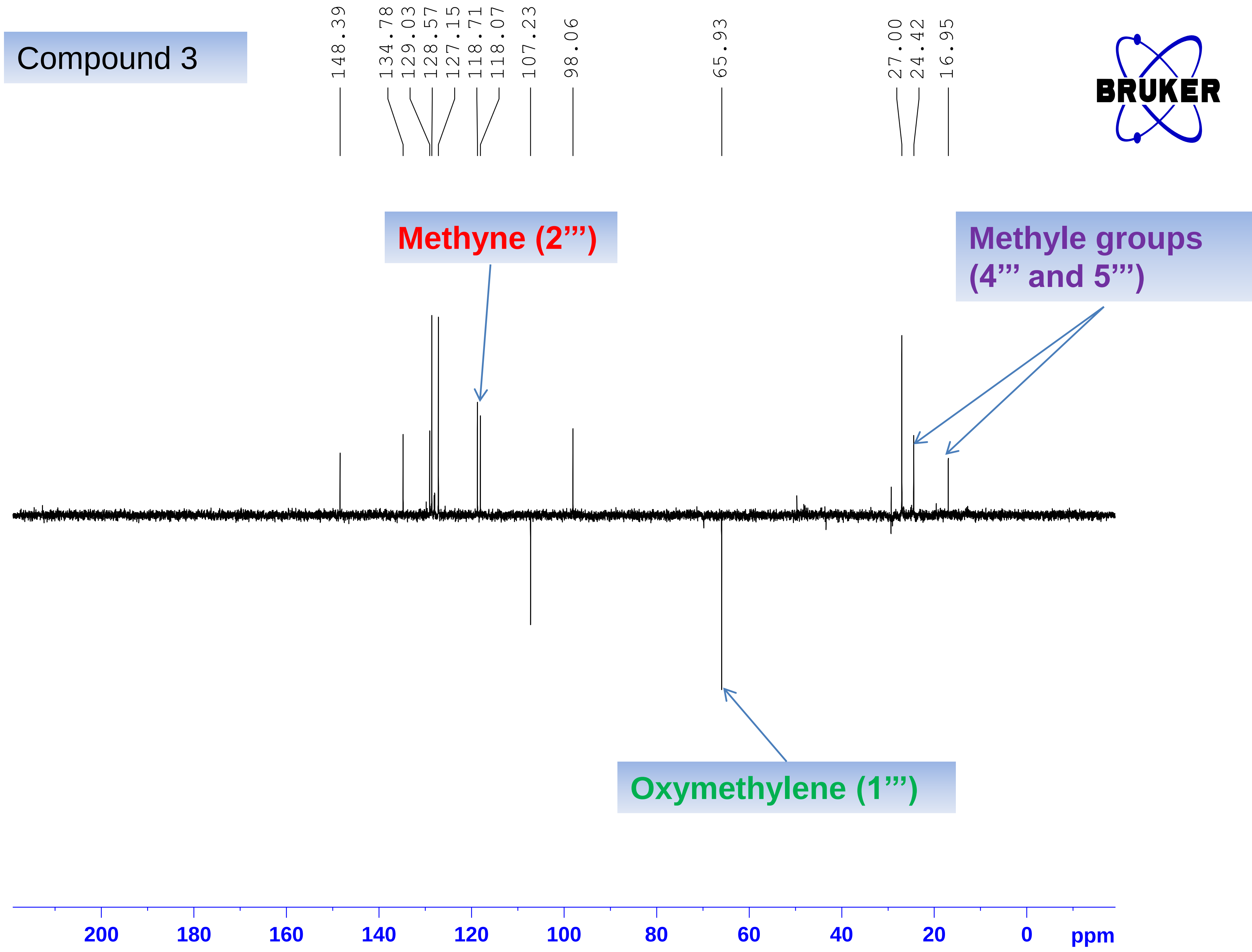
Compound 3
148.39
134.78
129.03
128.57
127.15
118.71
118.07
107.23
98.06
65.93
27.00
24.42
16.95
Methyne (2''')
Methyle groups (4''' and 5''')
Oxymethylene (1''')
200
180
160
140
120
100
80
60
40
20
0
ppm

Compound 3

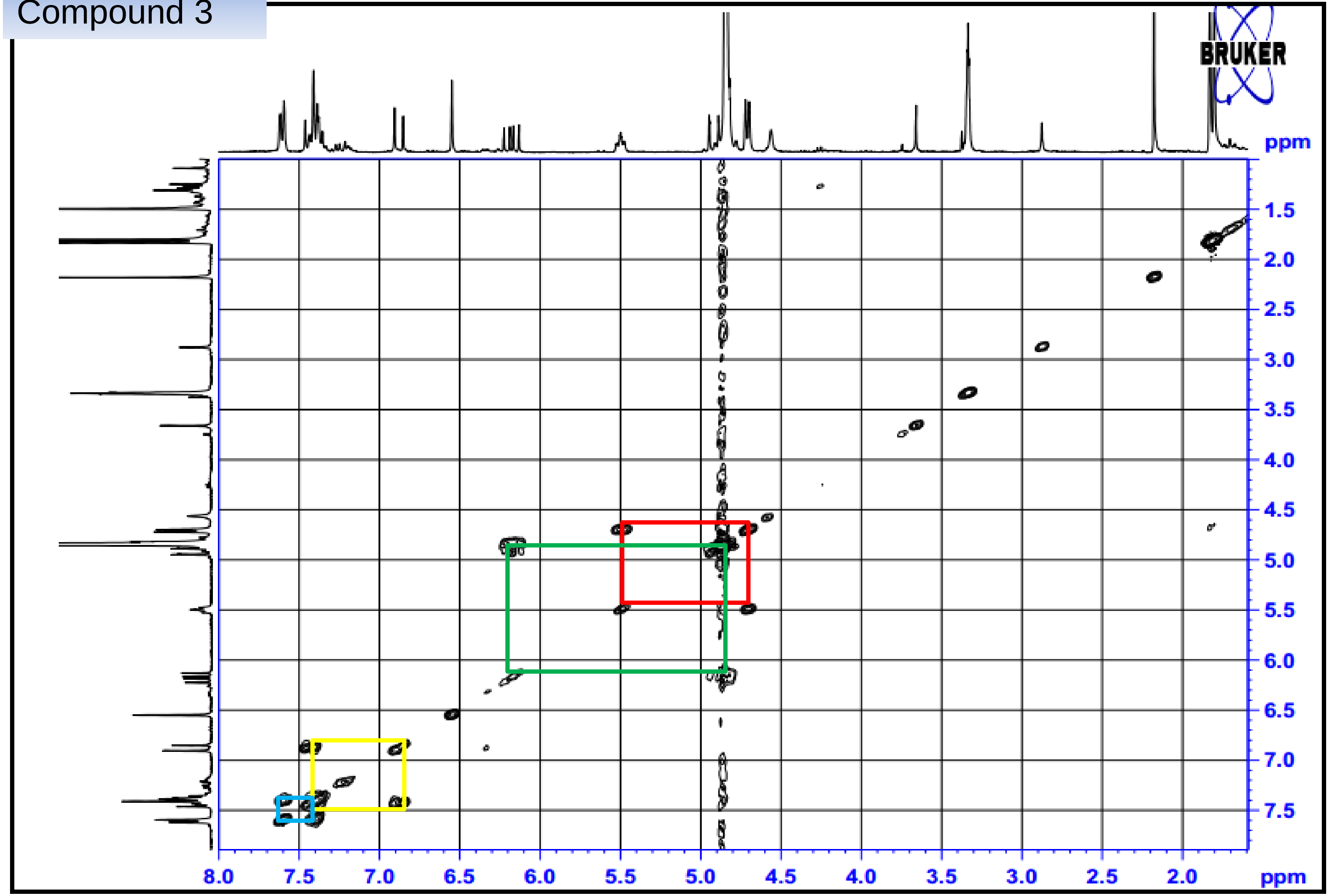


**COSY spectrum of compound 3**

Compound 3

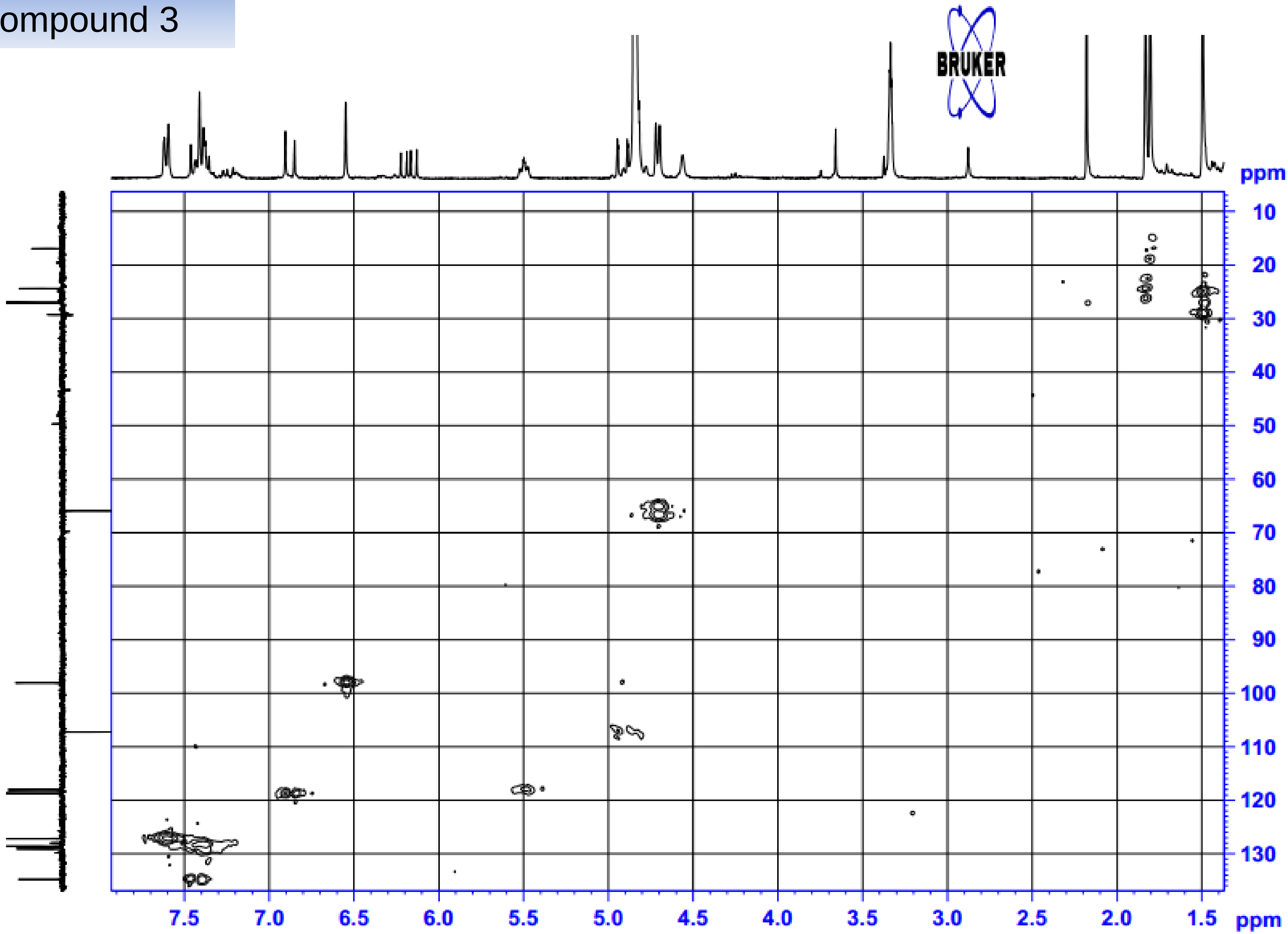


**HMQC spectrum of compound 3**

Compound 3

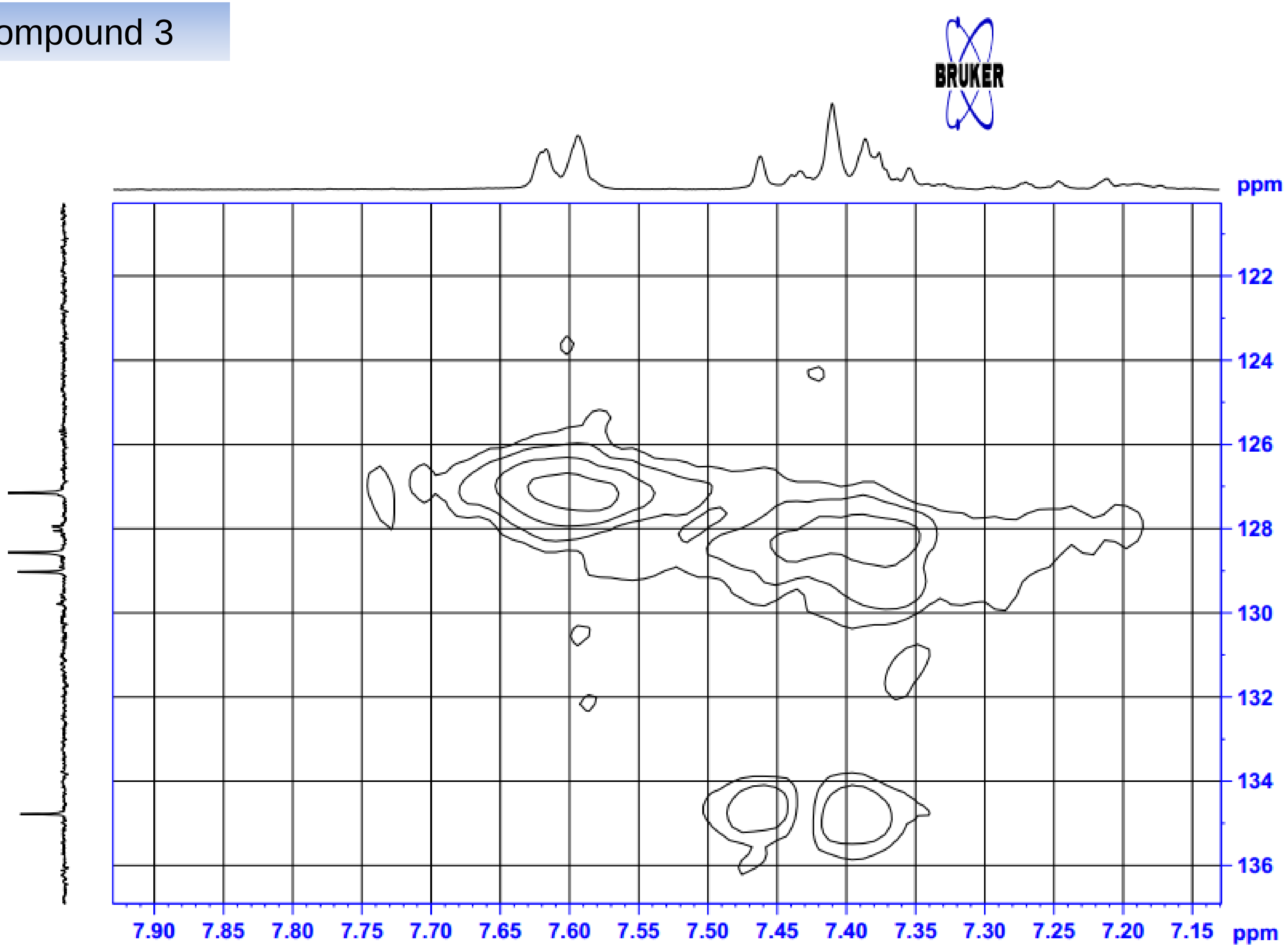


**HMQC spectrum of compound 3 continued**

Compound 3

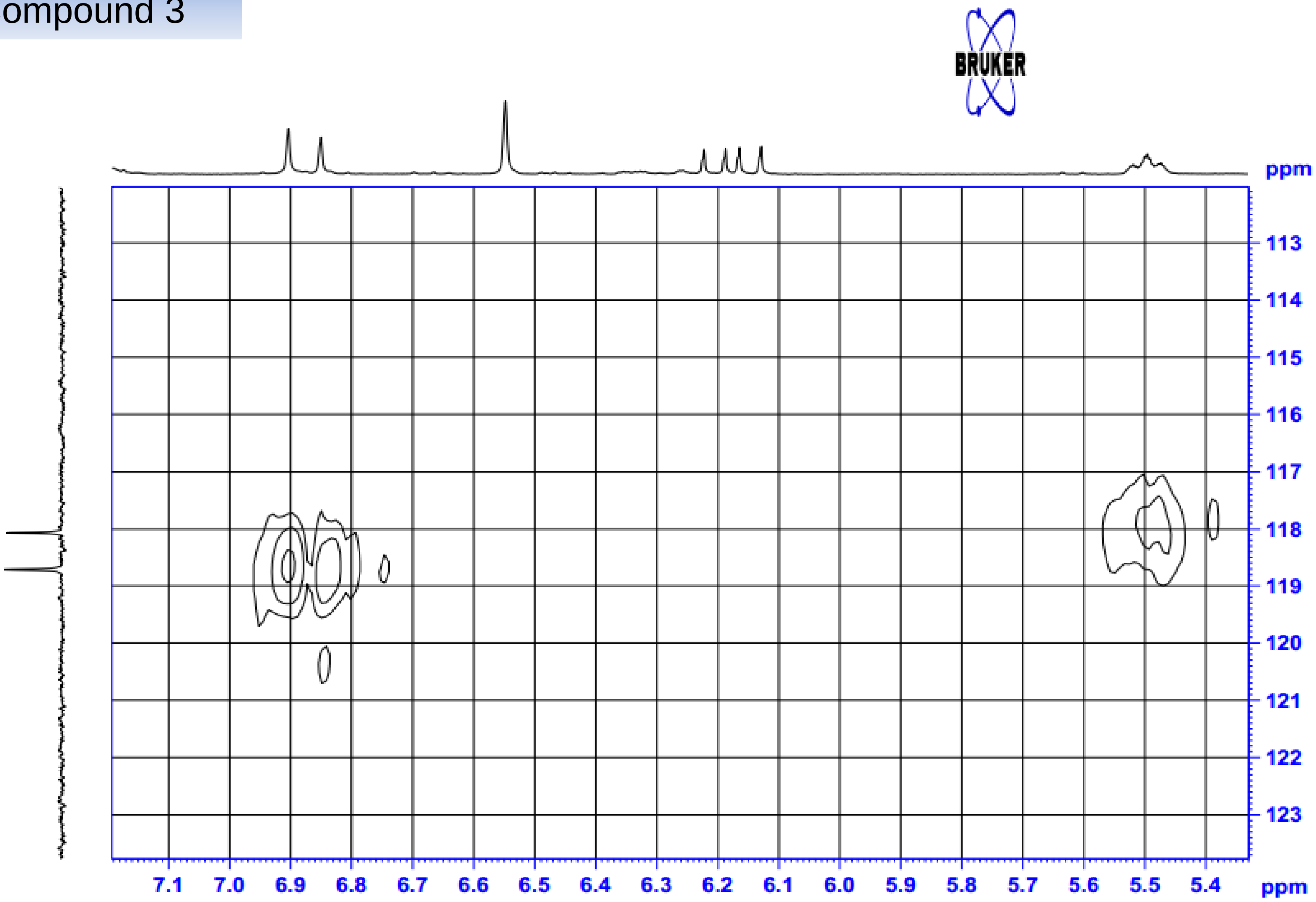


**HMQC spectrum of compound 3 continued**

# Full HMBC spectrum of compound 3

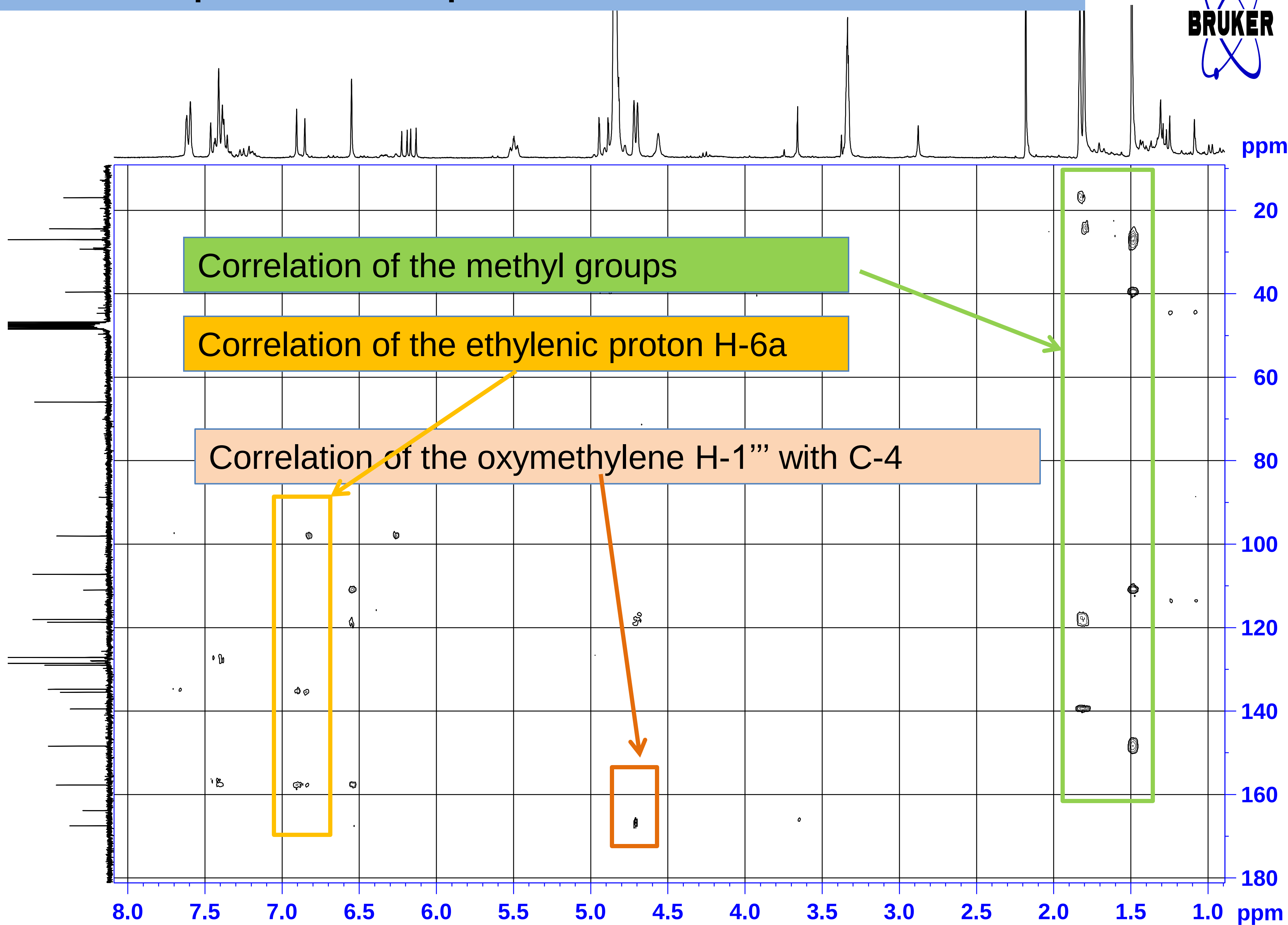

Compound 3

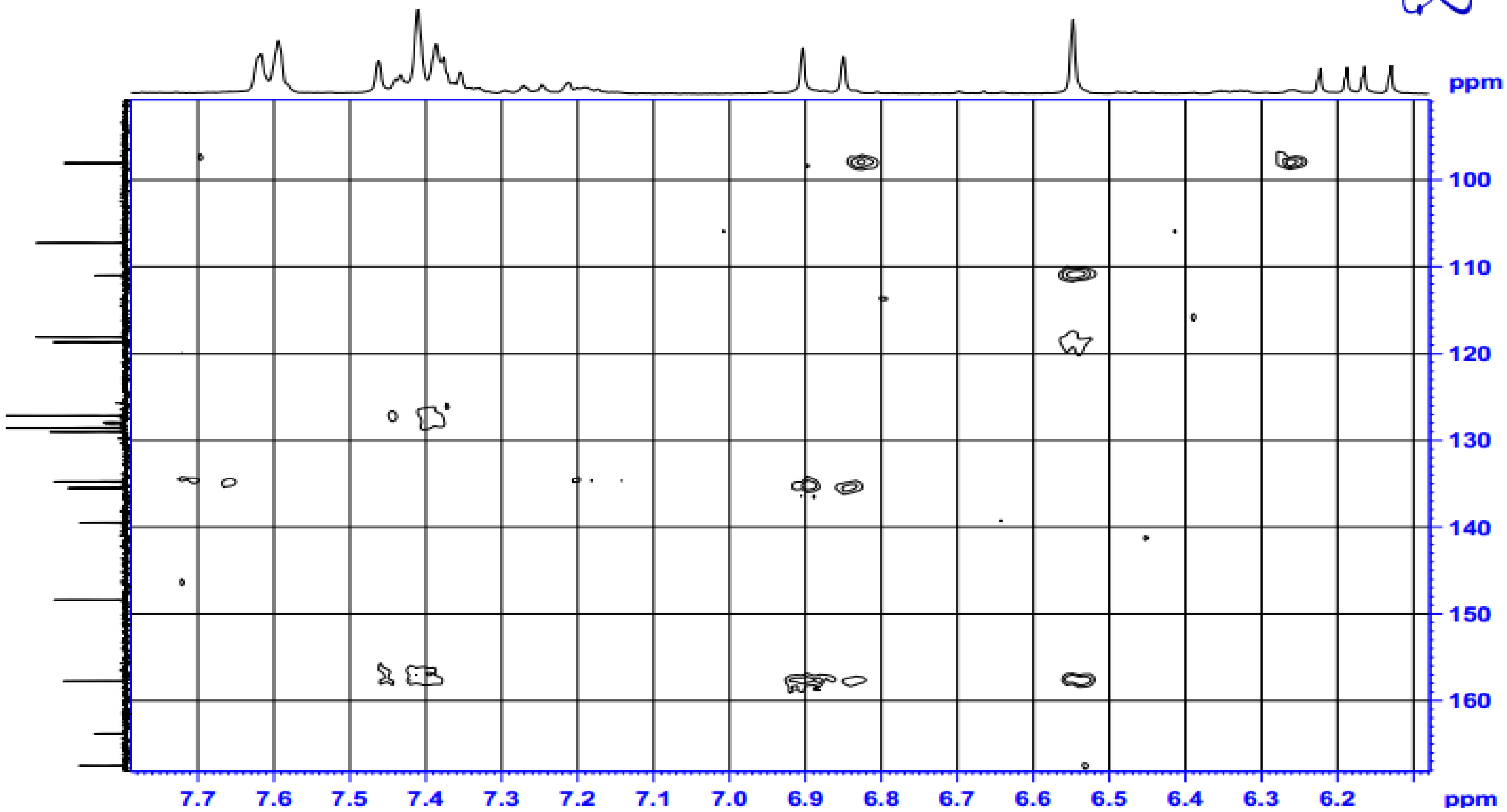


**HMBC spectrum of compound 3 (aromatic region)**

Compound 3

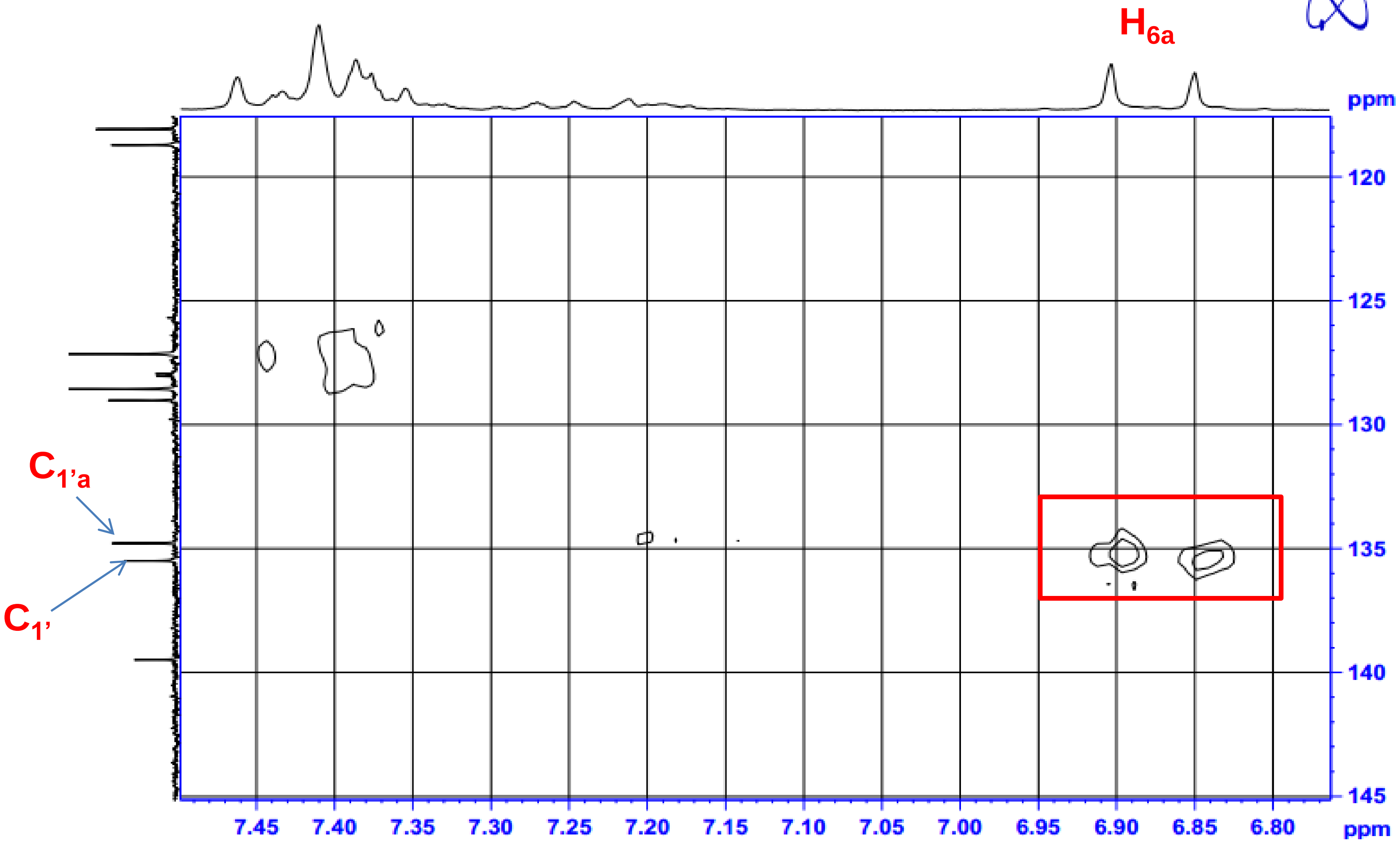


**HMBC correlations of the ethylenic proton H6a with C-1' and C-1'a**

Compound 3

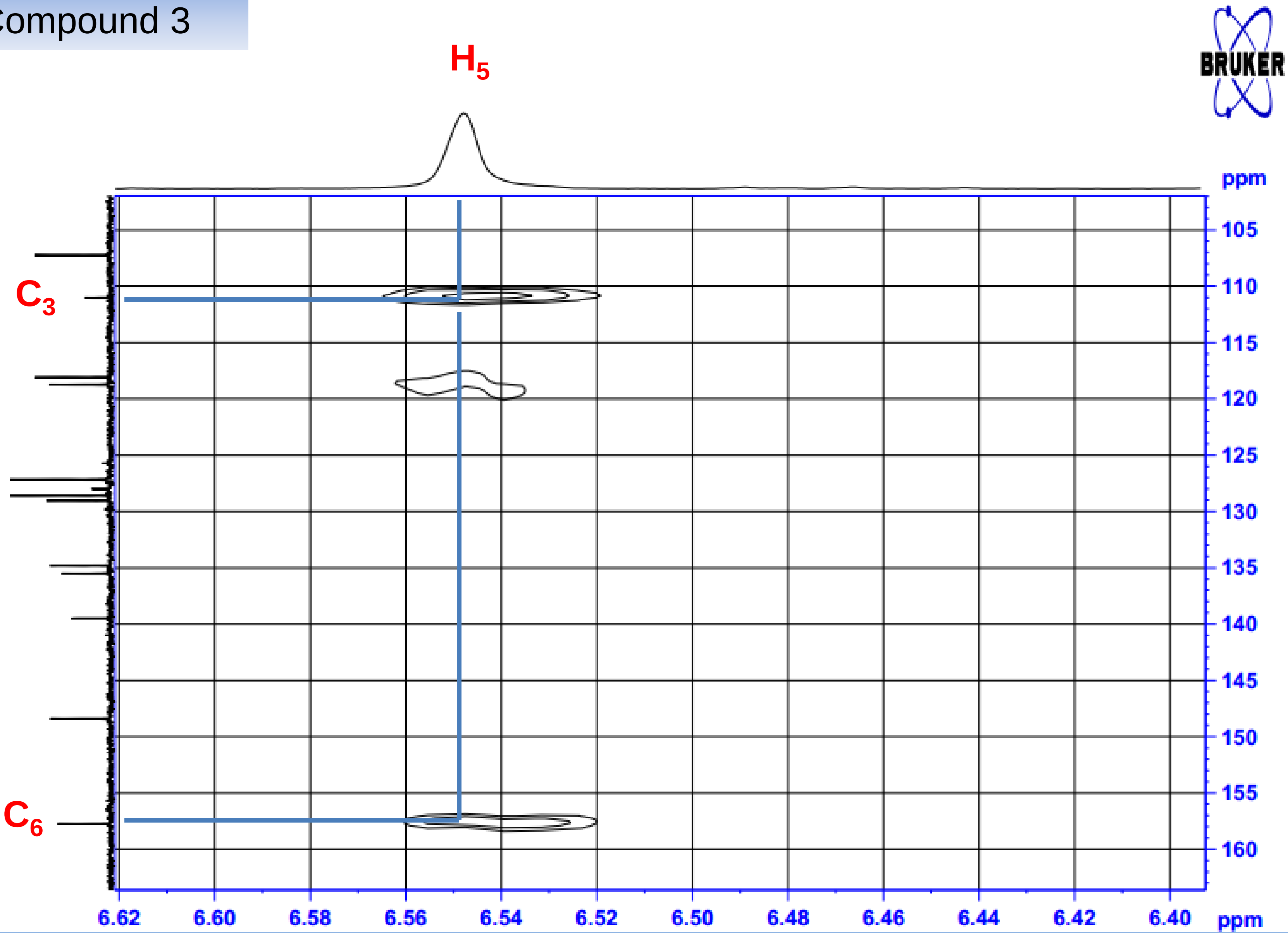


**HMBC correlations of the proton H6 of the penta-substituted aromatic ring**

Compound 3

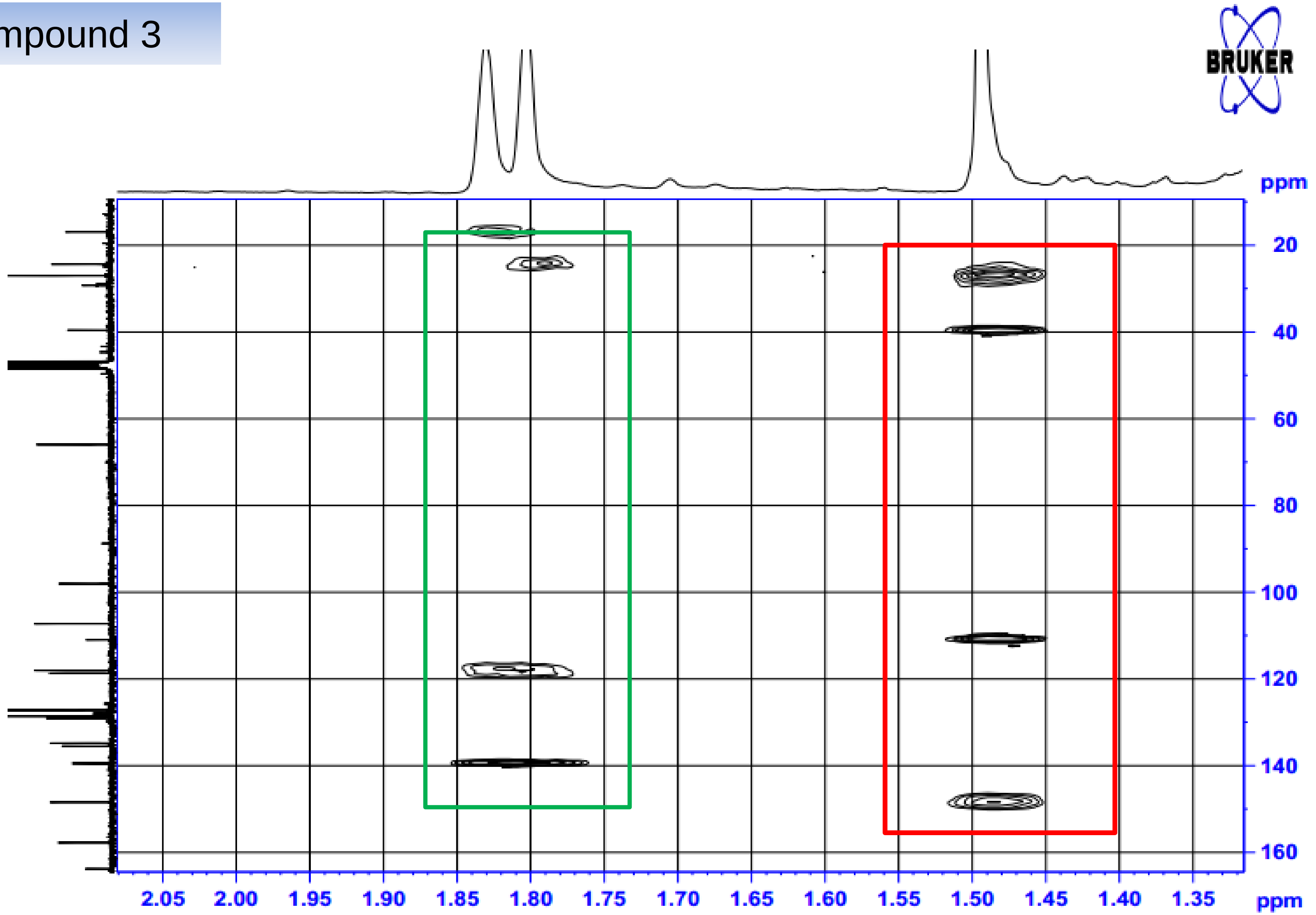


HMBC correlations of the methyles of the γ,γ-dimethylallyl group

HMBC correlations of the methyles of the α,α-dimethylallyl group
