# Supplementary material for: Two new pterocarpans and a new pyrone derivative with cytotoxic activities from Ptycholobium contortum (N.E.Br.) Brummitt (Leguminosae): revised NMR assignment of mundulea lactone
Source: Chem Cent J. 2016 Oct 5;10:58. doi: 10.1186/s13065-016-0204-x (PMC5050614; doi:10.1186/s13065-016-0204-x)

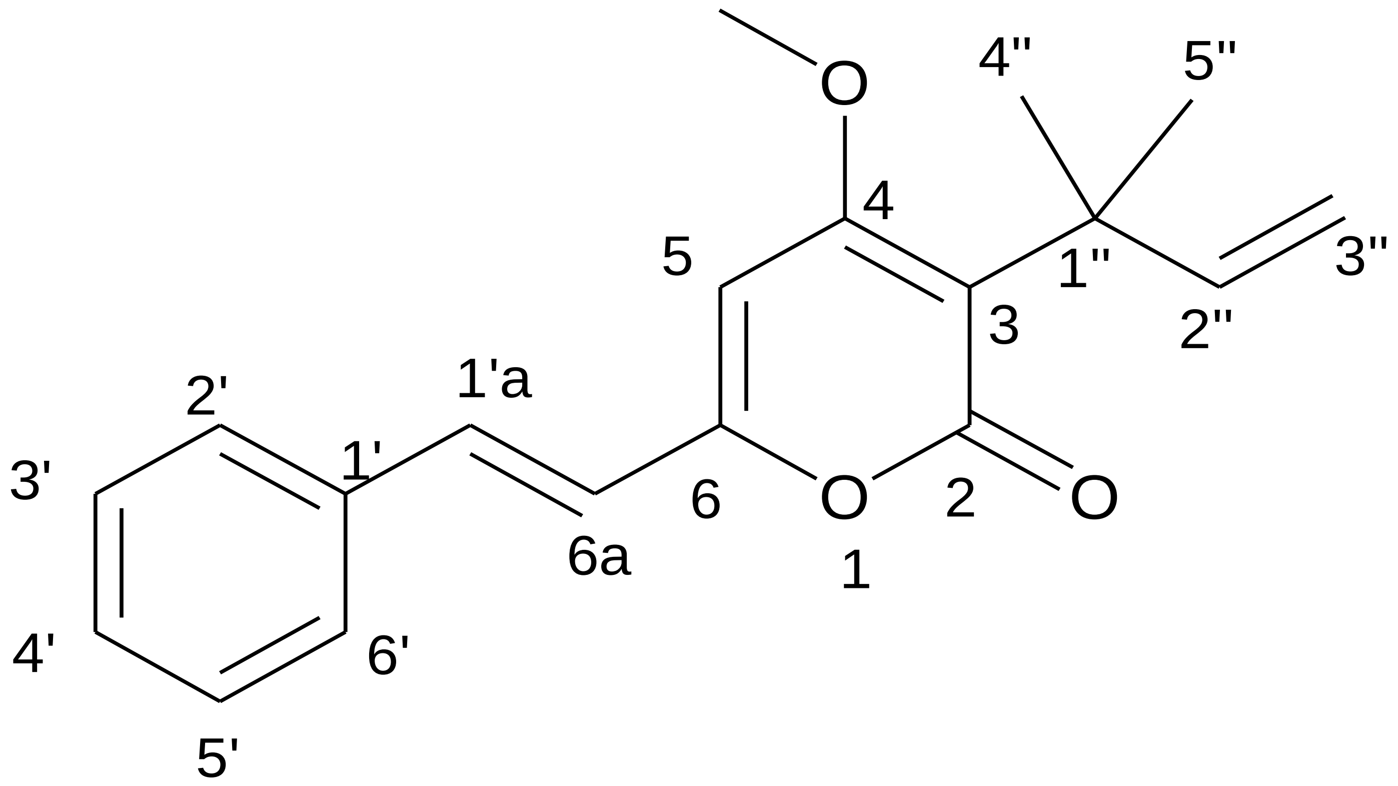

4

Mundulea lactone

IC4
PROTON CDCl3

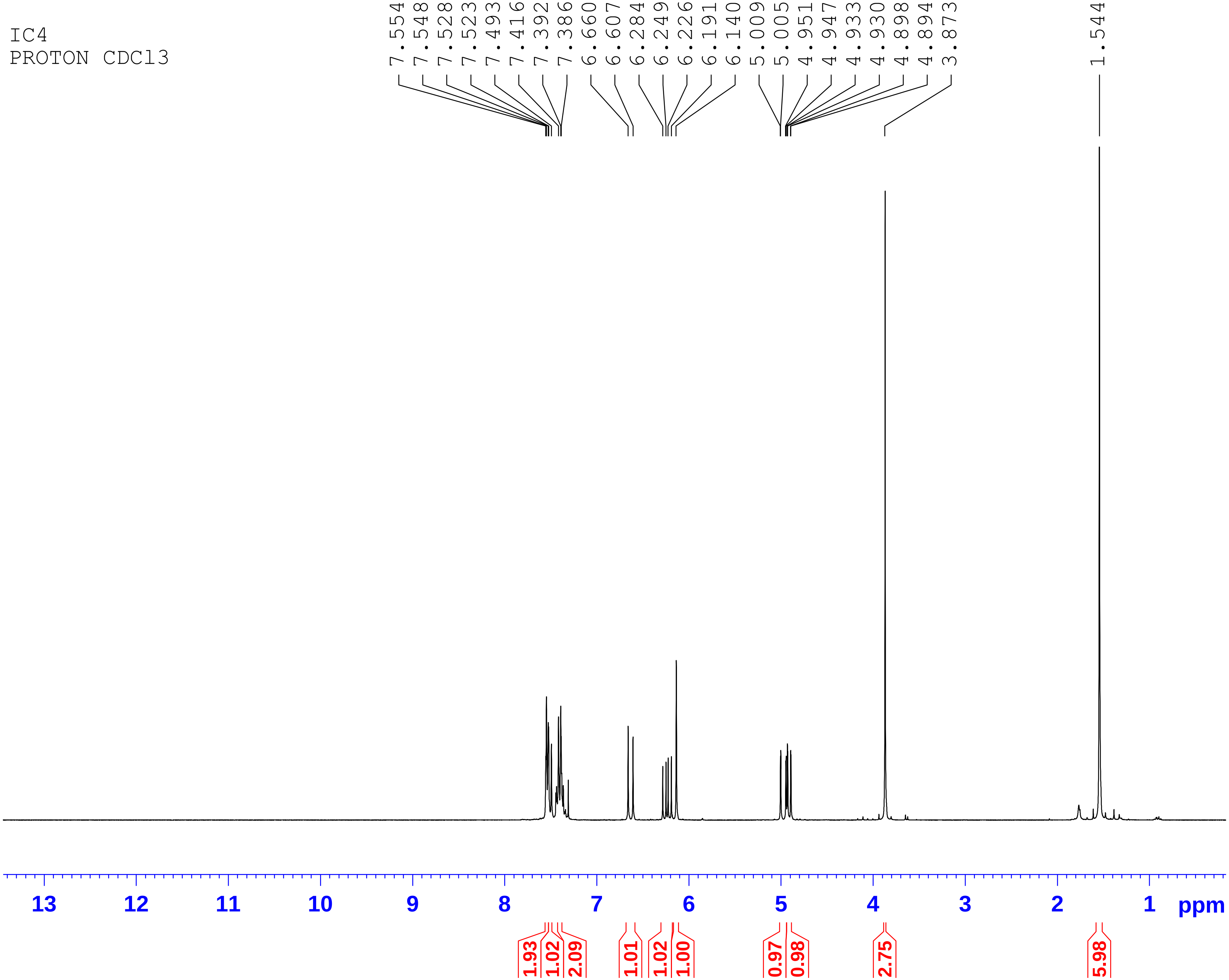

Current Data Parameters
NAME IC4
EXPNO 10
PROCNO 1

F2 - Acquisition Parameters
Date_ 20141027
Time 11.24
INSTRUM spect
PROBHD 5 mm QNP 1H/15
PULPROG zg30
TD 65536
SOLVENT CDCl3
NS 16
DS 2
SWH 6172.839 Hz
FIDRES 0.094190 Hz
AQ 5.3084660 sec
RG 228.1
DW 81.000 usec
DE 6.00 usec
TE 300.0 K
D1 1.00000000 sec
TD0 1

======== CHANNEL f1 ========
NUC1 1H
P1 6.06 usec
PL1 1.00 dB
SFO1 300.1318534 MHz

F2 - Processing parameters
SI 32768
SF 300.1300000 MHz
WDW EM
SSB 0
LB 0.30 Hz
GB 0
PC 1.00

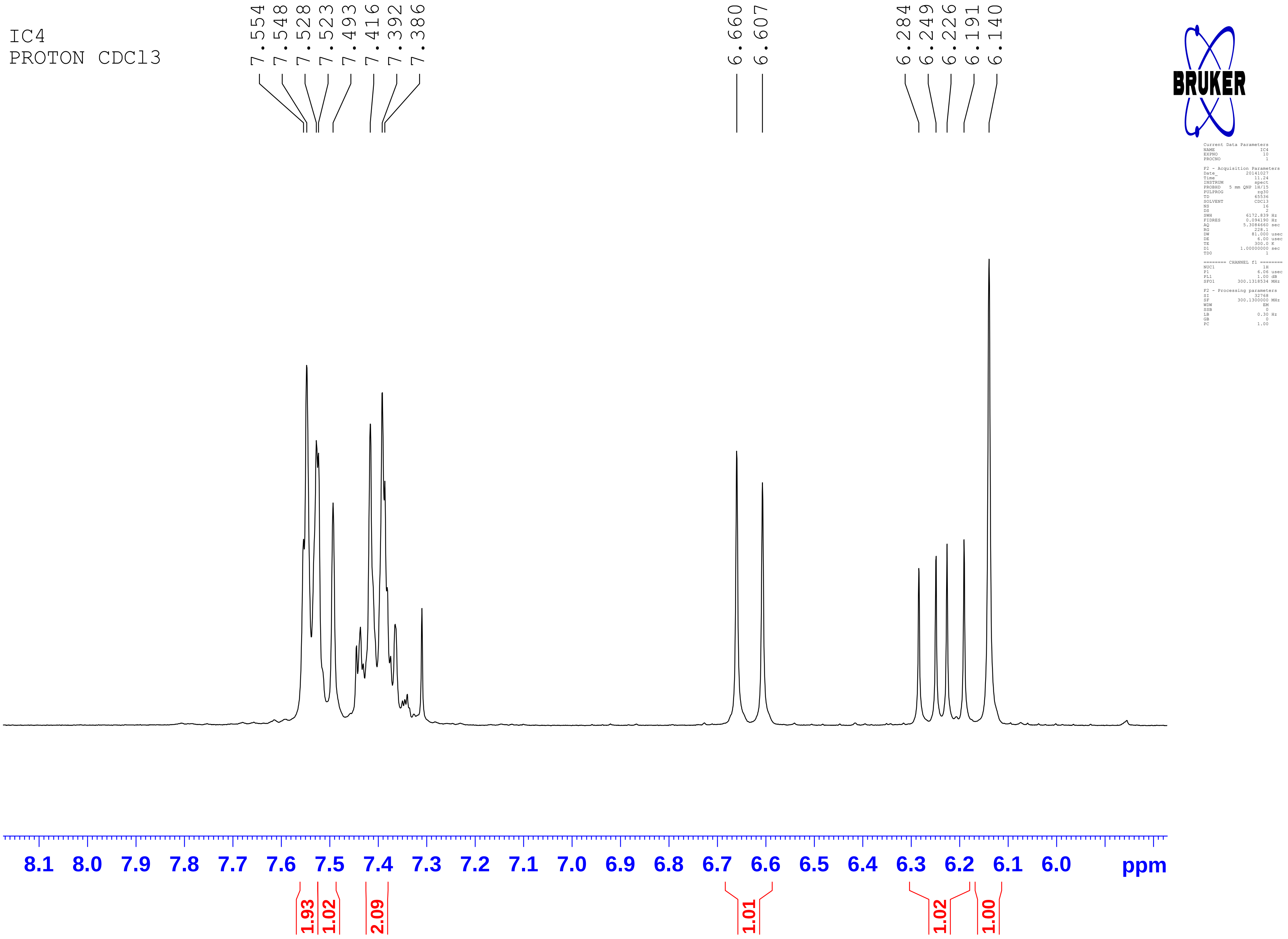
IC4
PROTON CDCl3
7.554
7.548
7.528
7.523
7.493
7.416
7.392
7.386
6.660
6.607
6.284
6.249
6.226
6.191
6.140
BRUKER
Current Data Parameters
NAME IC4
EXPNO 10
PROCNO 1
F2 - Acquisition Parameters
Date_ 20141027
Time 11.24
INSTRUM spect
PROBHD 5 mm QNP 1H/15
PULPROG zg30
TD 65536
SOLVENT CDCl3
NS 16
DS 2
SWH 6172.839 Hz
FIDRES 0.094190 Hz
AQ 5.3084660 sec
RG 228.1
DW 81.000 usec
DE 6.00 usec
TE 300.0 K
D1 1.00000000 sec
TD0 1
======== CHANNEL f1 ========
NUC1 1H
P1 6.06 usec
PL1 1.00 dB
SFO1 300.1318534 MHz
F2 - Processing parameters
SI 32768
SF 300.1300000 MHz
WDW EM
SSB 0
LB 0.30 Hz
GB 0
PC 1.00
8.1
8.0
7.9
7.8
7.7
7.6
7.5
7.4
7.3
7.2
7.1
7.0
6.9
6.8
6.7
6.6
6.5
6.4
6.3
6.2
6.1
6.0
ppm
1.93
1.02
2.09
1.01
1.02
1.00

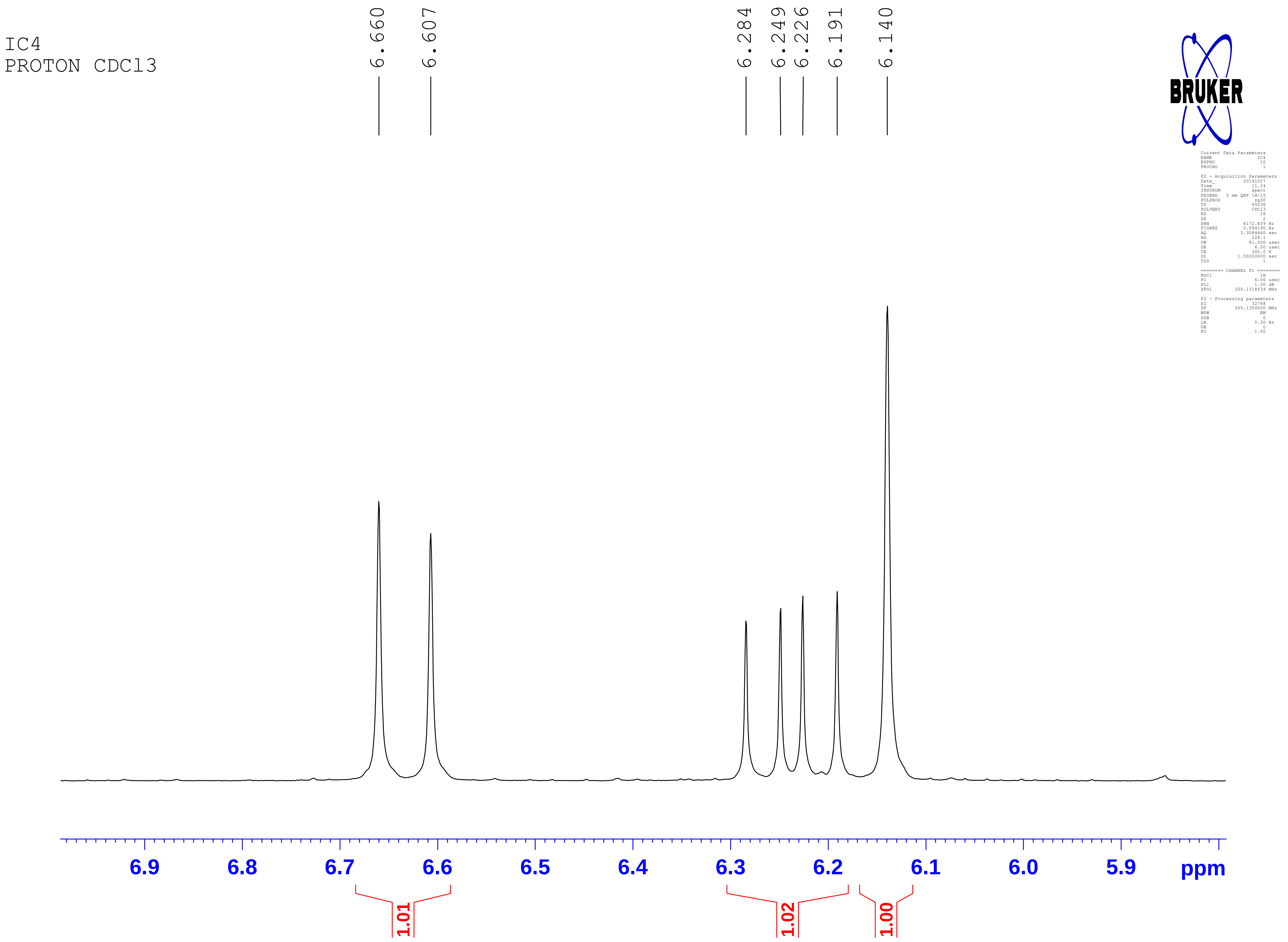
IC4
PROTON CDCl3
6.660
6.607
6.284
6.249
6.226
6.191
6.140
Current Data Parameters
NAME IC4
EXPNO 10
PROCNO 1
F2 - Acquisition Parameters
Date_ 20141027
Time 11.24
INSTRUM spect
PROBHD 5 mm QNP 1H/15
PULPROG zg30
TD 65536
SOLVENT CDCl3
NS 16
DS 2
SWH 6172.839 Hz
FIDRES 0.094190 Hz
AQ 5.3084660 sec
RG 228.1
DW 81.000 usec
DE 6.00 usec
TE 300.0 K
D1 1.00000000 sec
TD0 1
======== CHANNEL f1 ========
NUC1 1H
P1 6.06 usec
PL1 1.00 dB
SFO1 300.1318534 MHz
F2 - Processing parameters
SI 32768
SF 300.1300000 MHz
WDW EM
SSB 0
LB 0.30 Hz
GB 0
PC 1.00
6.9
6.8
6.7
6.6
6.5
6.4
6.3
6.2
6.1
6.0
5.9
ppm
1.01
1.02
1.00

IC4
PROTON CDCl3

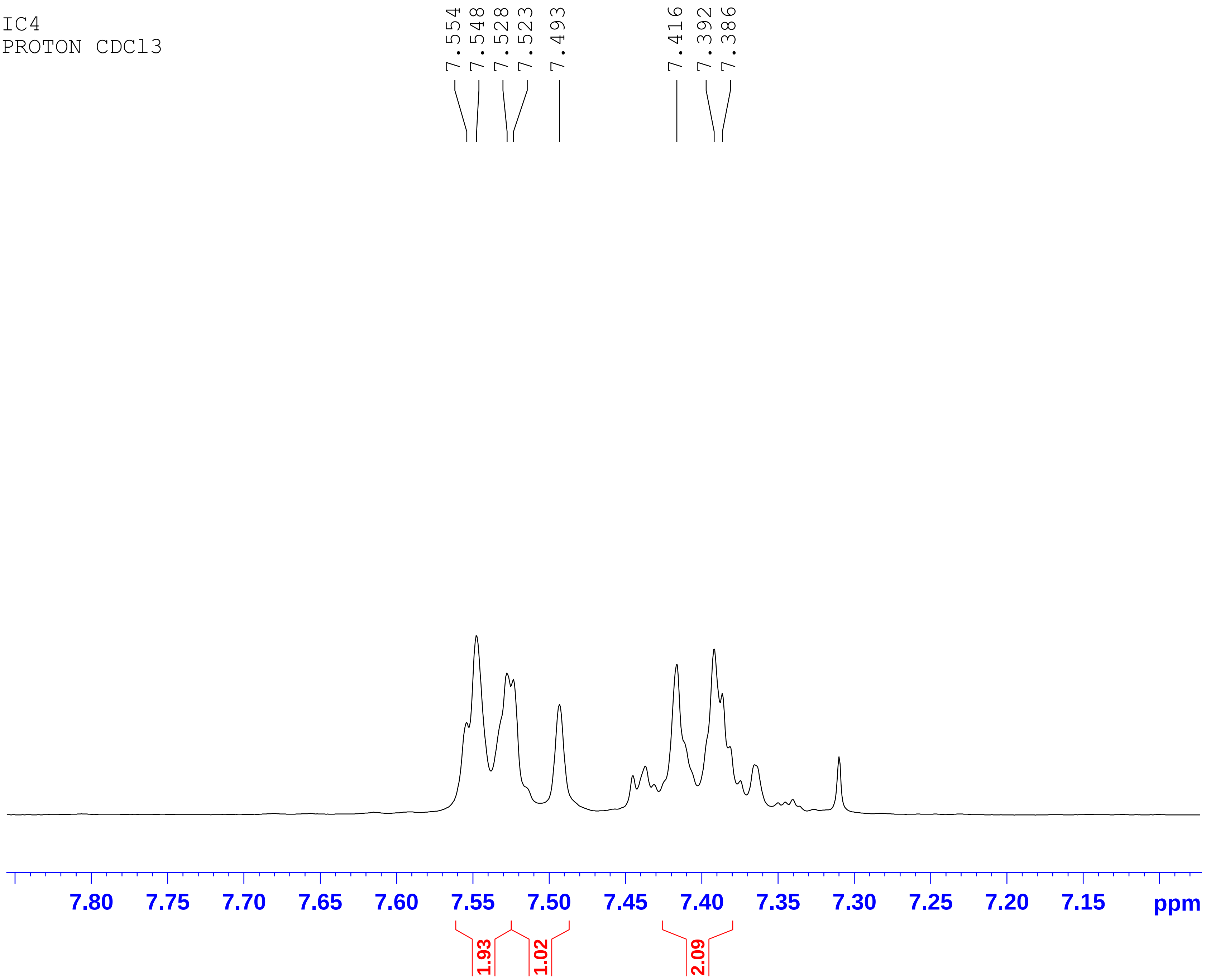

BRUKER

Current Data Parameters	
NAME	IC4
EXPNO	10
PROCNO	1

F2 - Acquisition Parameters	
Date_	20141027
Time	11.24
INSTRUM	spect
PROBHD	5 mm QNP 1H/15
PULPROG	zg30
TD	65536
SOLVENT	CDCl3
NS	16
DS	2
SWH	6172.839 Hz
FIDRES	0.094190 Hz
AQ	5.3084660 sec
RG	228.1
DW	81.000 usec
DE	6.00 usec
TE	300.0 K
D1	1.00000000 sec
TD0	1

======== CHANNEL f1 ========	
NUC1	1H
P1	6.06 usec
PL1	1.00 dB
SFO1	300.1318534 MHz

F2 - Processing parameters	
SI	32768
SF	300.1300000 MHz
WDW	EM
SSB	0
LB	0.30 Hz
GB	0
PC	1.00

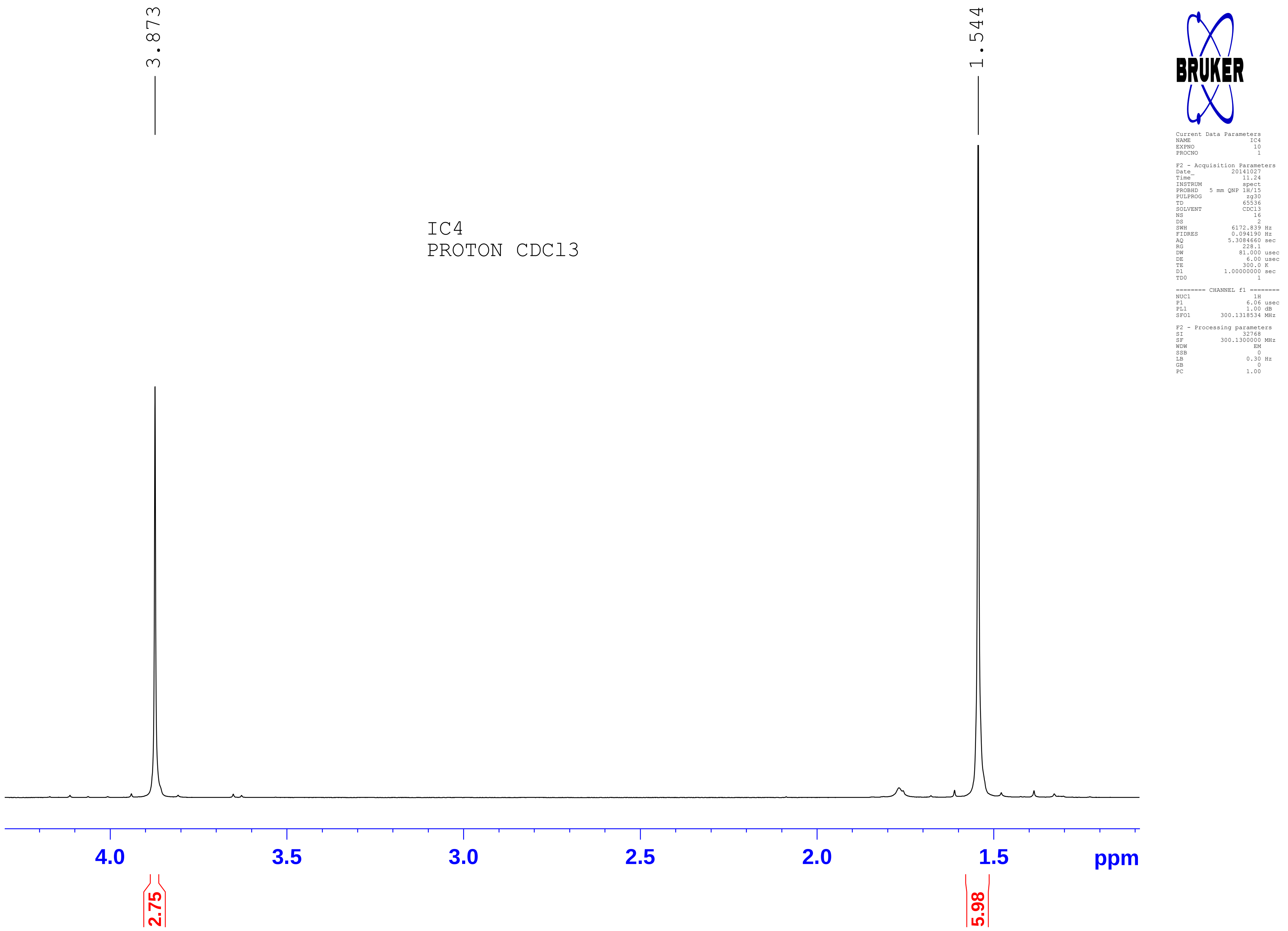
3.873
1.544
IC4
PROTON CDCl3
4.0
3.5
3.0
2.5
2.0
1.5
ppm
2.75
5.98
BRUKER
Current Data Parameters
NAME IC4
EXPNO 10
PROCNO 1
F2 - Acquisition Parameters
Date_ 20141027
Time 11.24
INSTRUM spect
PROBHD 5 mm QNP 1H/15
PULPROG zg30
TD 65536
SOLVENT CDCl3
NS 16
DS 2
SWH 6172.839 Hz
FIDRES 0.094190 Hz
AQ 5.3084660 sec
RG 228.1
DW 81.000 usec
DE 6.00 usec
TE 300.0 K
D1 1.00000000 sec
TD0 1
======== CHANNEL f1 ========
NUC1 1H
P1 6.06 usec
PL1 1.00 dB
SFO1 300.1318534 MHz
F2 - Processing parameters
SI 32768
SF 300.1300000 MHz
WDW EM
SSB 0
LB 0.30 Hz
GB 0
PC 1.00

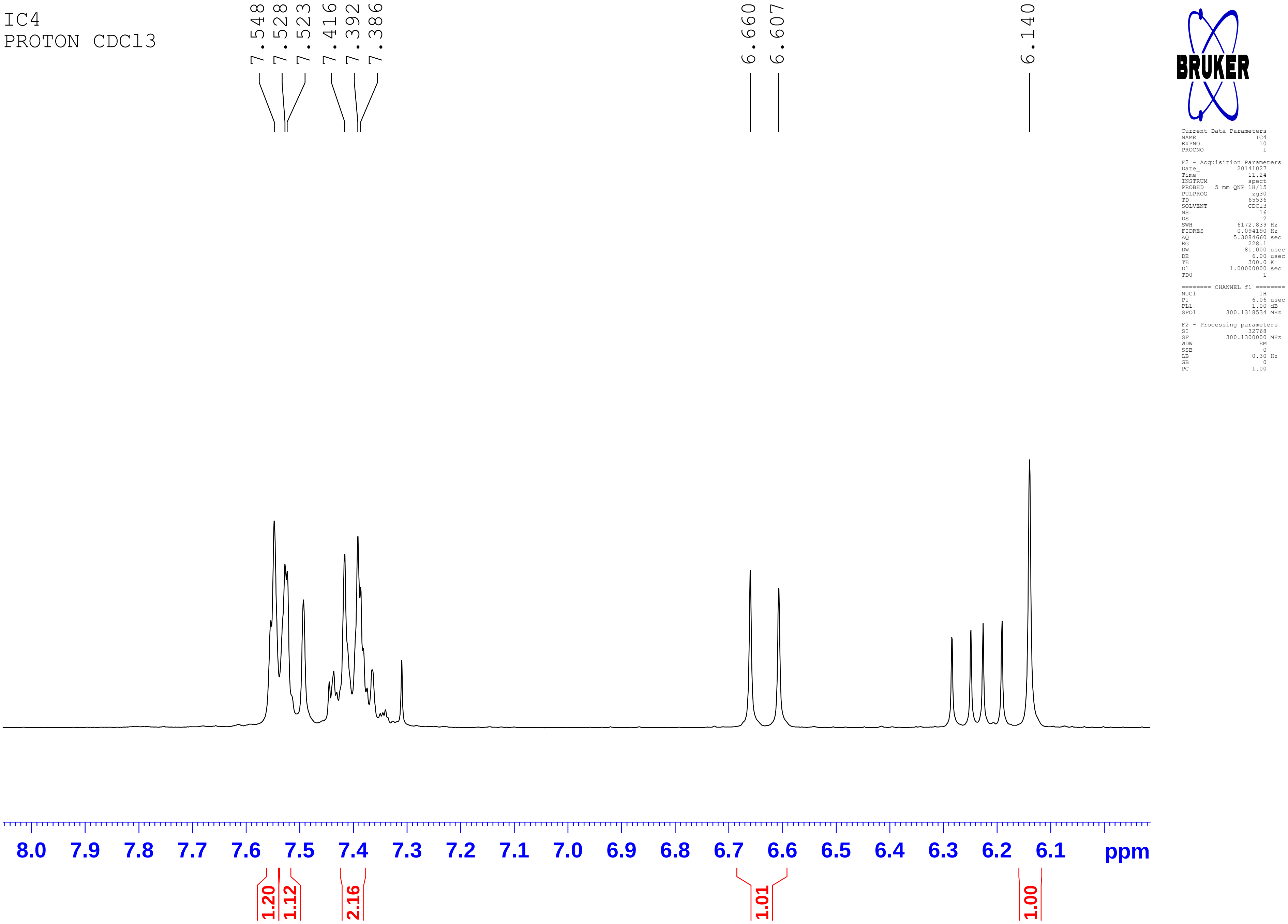
IC4
PROTON CDCl3
7.548
7.528
7.523
7.416
7.392
7.386
6.660
6.607
6.140
Current Data Parameters
NAME IC4
EXPNO 10
PROCNO 1
F2 - Acquisition Parameters
Date_ 20141027
Time 11.24
INSTRUM spect
PROBHD 5 mm QNP 1H/15
PULPROG zg30
TD 65536
SOLVENT CDCl3
NS 16
DS 2
SWH 6172.839 Hz
FIDRES 0.094190 Hz
AQ 5.3084660 sec
RG 228.1
DW 81.000 usec
DE 6.00 usec
TE 300.0 K
D1 1.00000000 sec
TD0 1
======== CHANNEL f1 ========
NUC1 1H
P1 6.06 usec
PL1 1.00 dB
SFO1 300.1318534 MHz
F2 - Processing parameters
SI 32768
SF 300.1300000 MHz
WDW EM
SSB 0
LB 0.30 Hz
GB 0
PC 1.00
8.0
7.9
7.8
7.7
7.6
7.5
7.4
7.3
7.2
7.1
7.0
6.9
6.8
6.7
6.6
6.5
6.4
6.3
6.2
6.1
ppm
1.20
1.12
2.16
1.01
1.00

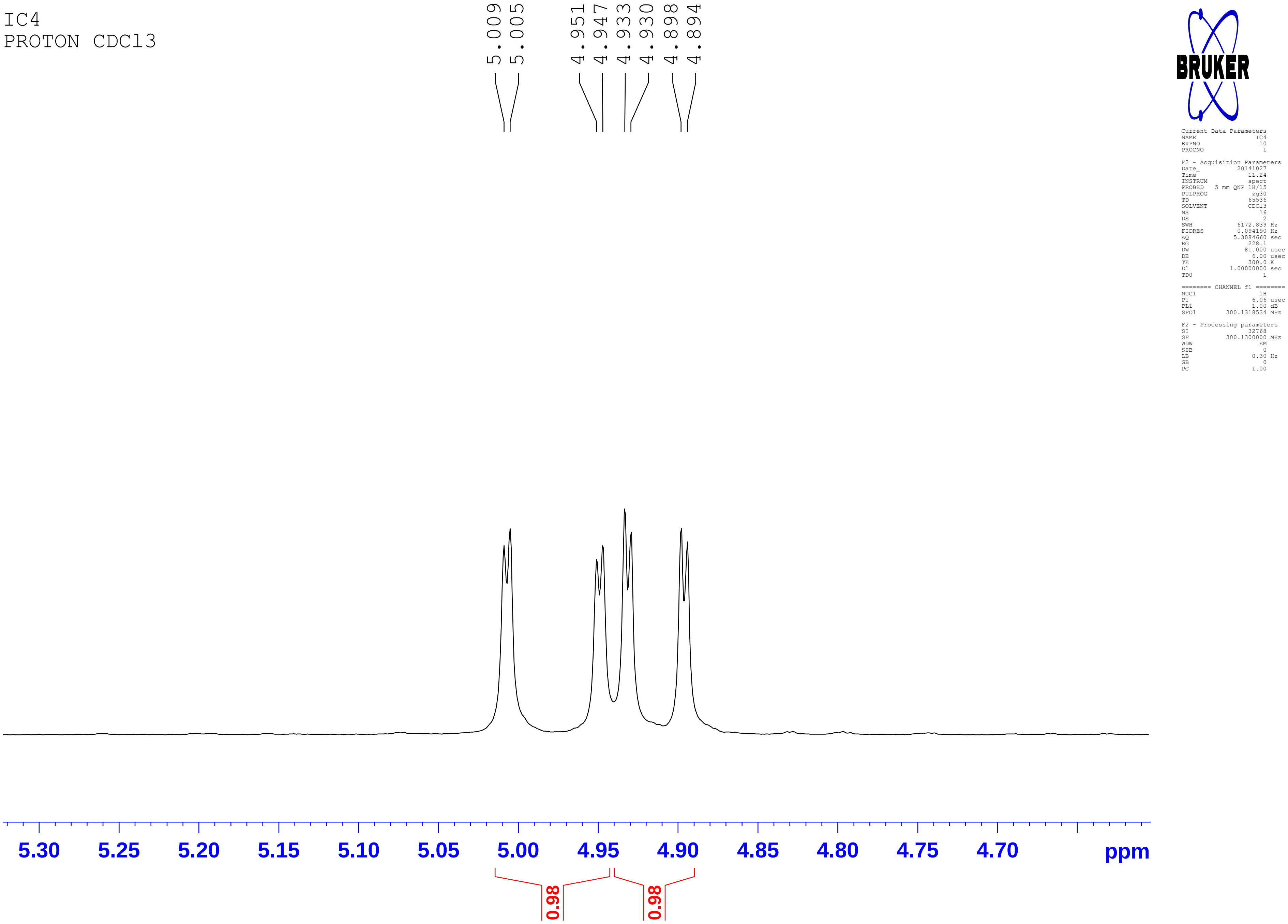

IC4
PROTON CDCl3
5.009
5.005
4.951
4.947
4.933
4.930
4.898
4.894
Current Data Parameters
NAME IC4
EXPNO 10
PROCNO 1
F2 - Acquisition Parameters
Date_ 20141027
Time 11.24
INSTRUM spect
PROBHD 5 mm QNP 1H/15
PULPROG zg30
TD 65536
SOLVENT CDCl3
NS 16
DS 2
SWH 6172.839 Hz
FIDRES 0.094190 Hz
AQ 5.3084660 sec
RG 228.1
DW 81.000 usec
DE 6.00 usec
TE 300.0 K
D1 1.00000000 sec
TD0 1
======== CHANNEL f1 ========
NUC1 1H
P1 6.06 usec
PL1 1.00 dB
SFO1 300.1318534 MHz
F2 - Processing parameters
SI 32768
SF 300.1300000 MHz
WDW EM
SSB 0
LB 0.30 Hz
GB 0
PC 1.00
5.30
5.25
5.20
5.15
5.10
5.05
5.00
4.95
4.90
4.85
4.80
4.75
4.70
ppm
0.98
0.98

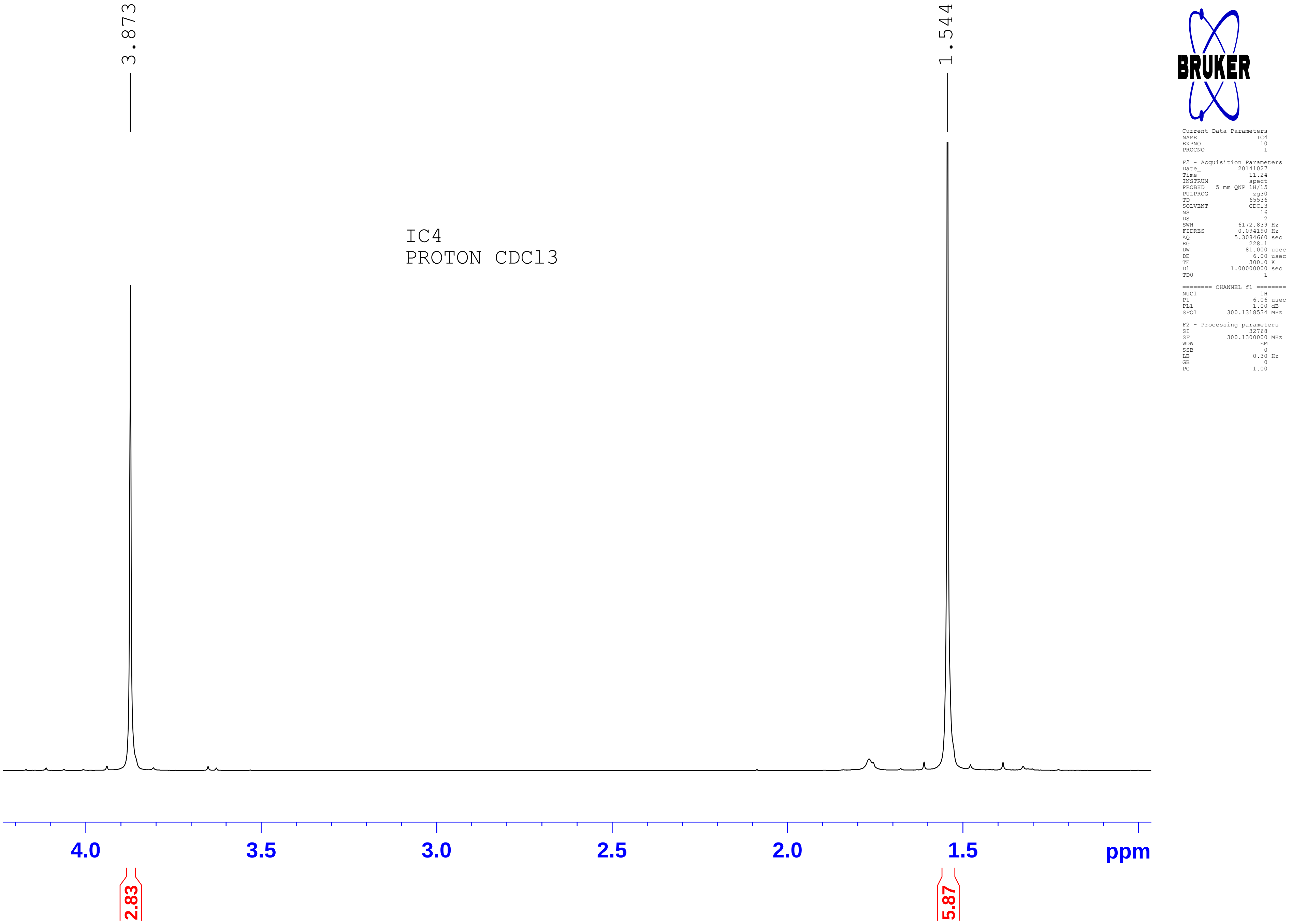

3.873
1.544
IC4
PROTON CDCl3
4.0
3.5
3.0
2.5
2.0
1.5
ppm
2.83
5.87
Current Data Parameters
NAME IC4
EXPNO 10
PROCNO 1
F2 - Acquisition Parameters
Date_ 20141027
Time 11.24
INSTRUM spect
PROBHD 5 mm QNP 1H/15
PULPROG zg30
TD 65536
SOLVENT CDCl3
NS 16
DS 2
SWH 6172.839 Hz
FIDRES 0.094190 Hz
AQ 5.3084660 sec
RG 228.1
DW 81.000 usec
DE 6.00 usec
TE 300.0 K
D1 1.00000000 sec
TD0 1
======== CHANNEL f1 ========
NUC1 1H
P1 6.06 usec
PL1 1.00 dB
SFO1 300.1318534 MHz
F2 - Processing parameters
SI 32768
SF 300.1300000 MHz
WDW EM
SSB 0
LB 0.30 Hz
GB 0
PC 1.00

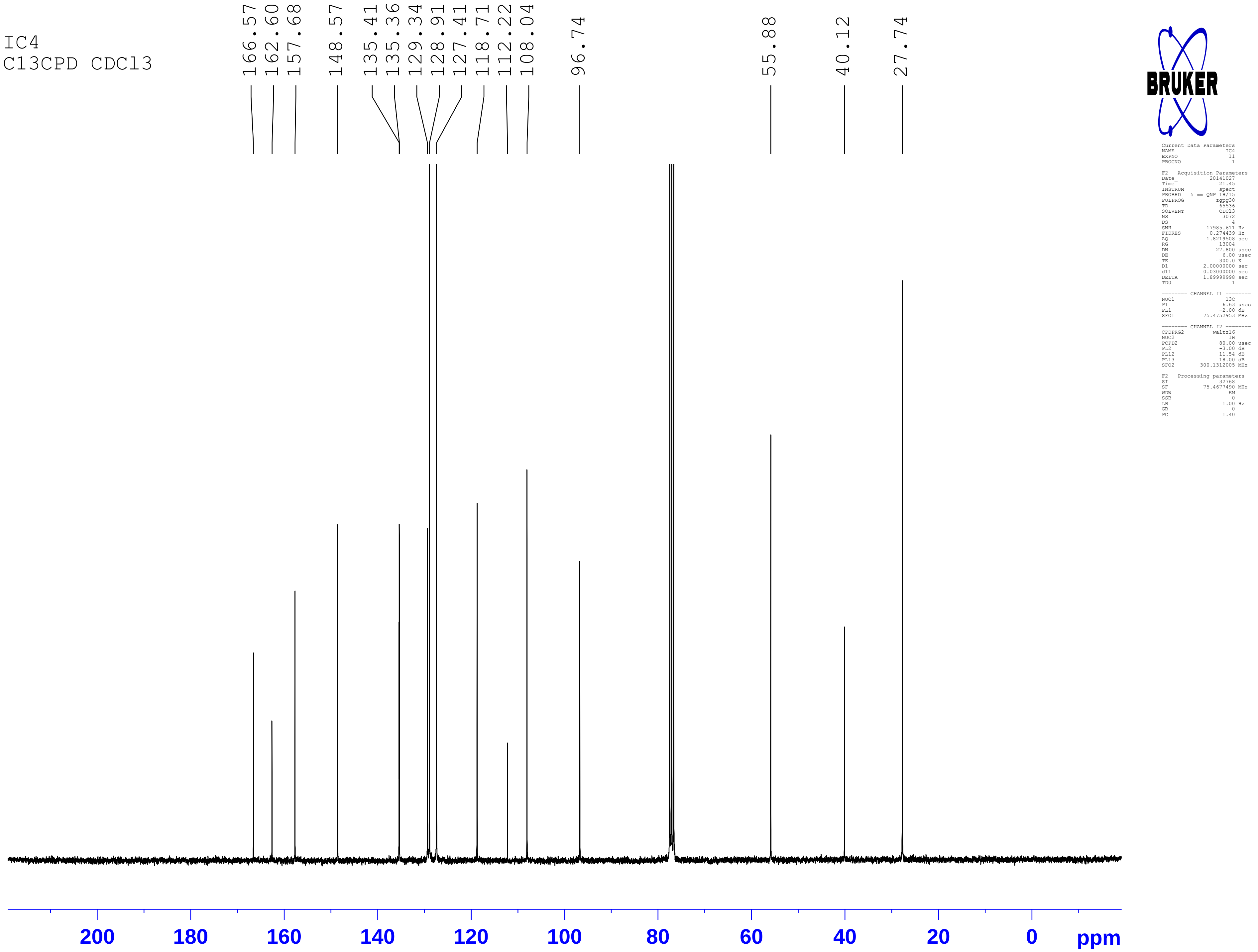

IC4
C13CPD CDCl3
166.57
162.60
157.68
148.57
135.41
135.36
129.34
128.91
127.41
118.71
112.22
108.04
96.74
55.88
40.12
27.74
BRUKER
Current Data Parameters
NAME IC4
EXPNO 11
PROCNO 1
F2 - Acquisition Parameters
Date_ 20141027
Time 21.45
INSTRUM spect
PROBHD 5 mm QNP 1H/15
PULPROG zgpg30
TD 65536
SOLVENT CDCl3
NS 3072
DS 4
SWH 17985.611 Hz
FIDRES 0.274439 Hz
AQ 1.8219508 sec
RG 13004
DW 27.800 usec
DE 6.00 usec
TE 300.0 K
D1 2.00000000 sec
d11 0.03000000 sec
DELTA 1.89999998 sec
TD0 1
======== CHANNEL f1 ========
NUC1 13C
P1 6.63 usec
PL1 -2.00 dB
SFO1 75.4752953 MHz
======== CHANNEL f2 ========
CPDPRG2 waltz16
NUC2 1H
PCPD2 80.00 usec
PL2 -3.00 dB
PL12 11.54 dB
PL13 18.00 dB
SFO2 300.1312005 MHz
F2 - Processing parameters
SI 32768
SF 75.4677490 MHz
WDW EM
SSB 0
LB 1.00 Hz
GB 0
PC 1.40
200
180
160
140
120
100
80
60
40
20
0
ppm

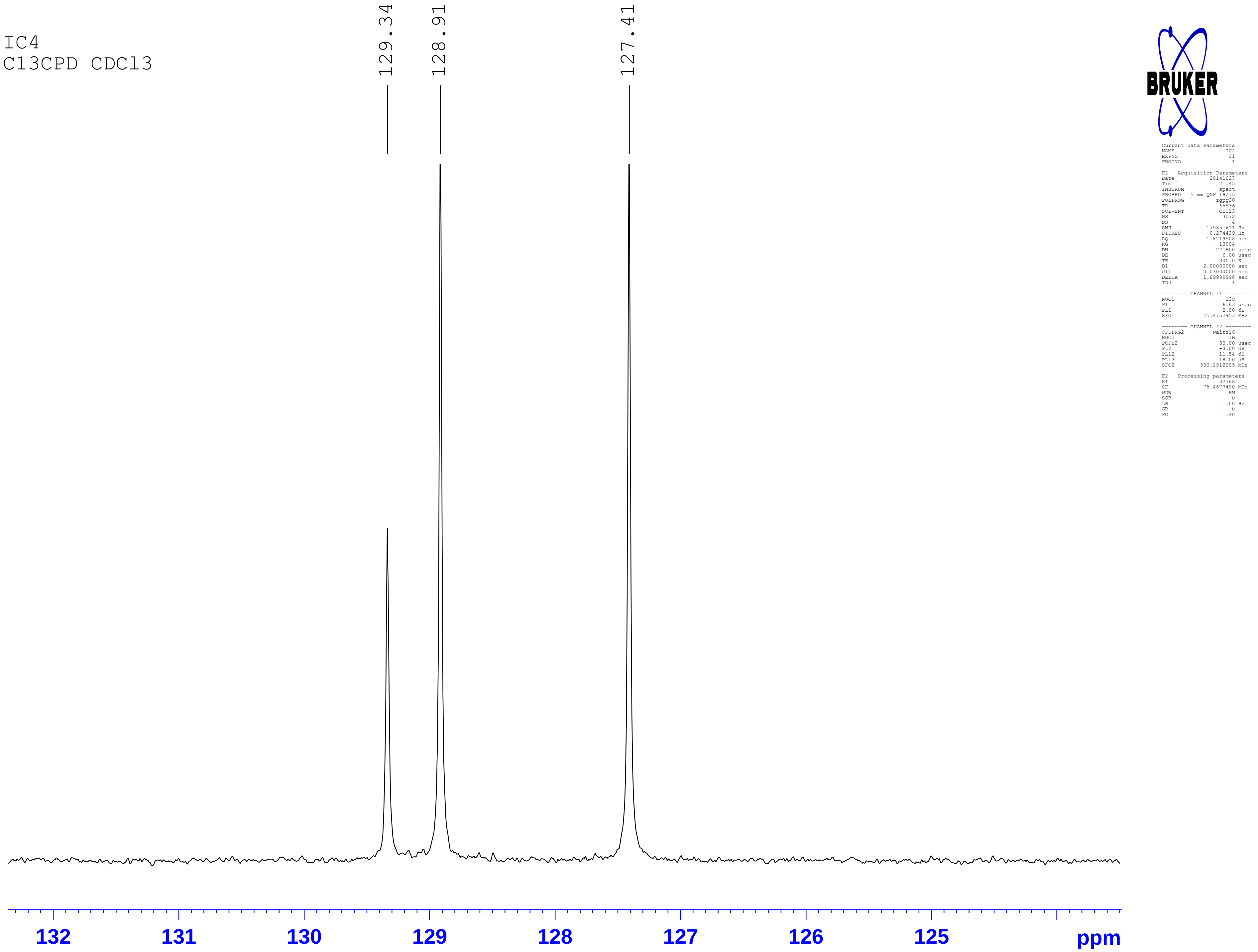

IC4
C13CPD CDCl3
129.34
128.91
127.41
132
131
130
129
128
127
126
125
ppm
BRUKER
Current Data Parameters
NAME IC4
EXPNO 11
PROCNO 1
F2 - Acquisition Parameters
Date_ 20141027
Time 21.45
INSTRUM spect
PROBHD 5 mm QNP 1H/15
PULPROG zgpg30
TD 65536
SOLVENT CDCl3
NS 3072
DS 4
SWH 17985.611 Hz
FIDRES 0.274439 Hz
AQ 1.8219508 sec
RG 13004
DW 27.800 usec
DE 6.00 usec
TE 300.0 K
D1 2.00000000 sec
d11 0.03000000 sec
DELTA 1.89999998 sec
TD0 1
======== CHANNEL f1 ========
NUC1 13C
P1 6.63 usec
PL1 -2.00 dB
SFO1 75.4752953 MHz
======== CHANNEL f2 ========
CPDPRG2 waltz16
NUC2 1H
PCPD2 80.00 usec
PL2 -3.00 dB
PL12 11.54 dB
PL13 18.00 dB
SFO2 300.1312005 MHz
F2 - Processing parameters
SI 32768
SF 75.4677490 MHz
WDW EM
SSB 0
LB 1.00 Hz
GB 0
PC 1.40

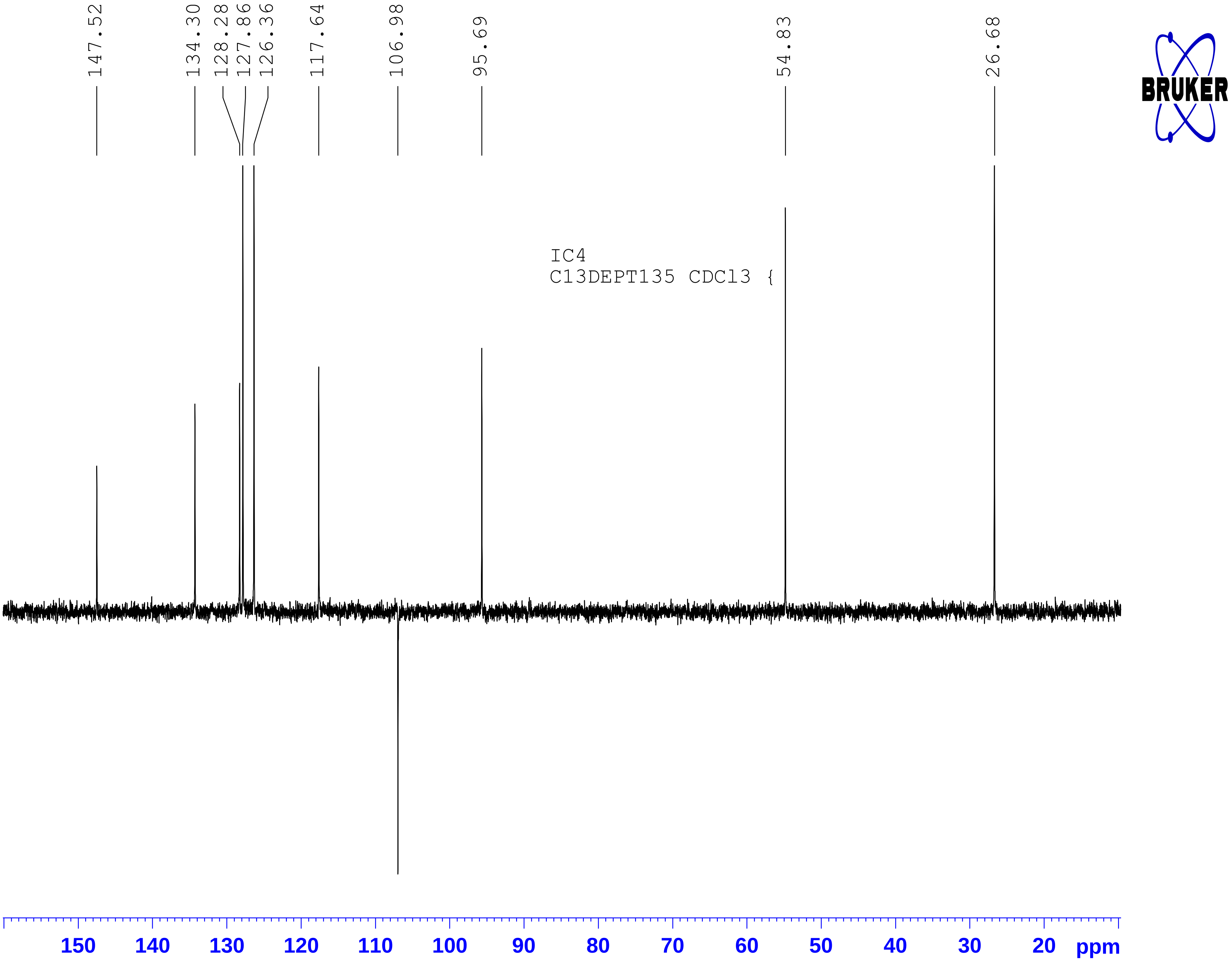
147.52
134.30
128.28
127.86
126.36
117.64
106.98
95.69
54.83
26.68
BRUKER
IC4
C13DEPT135 CDCl3 {
150
140
130
120
110
100
90
80
70
60
50
40
30
20
ppm

IC4
HMQCGP CDCl3 {

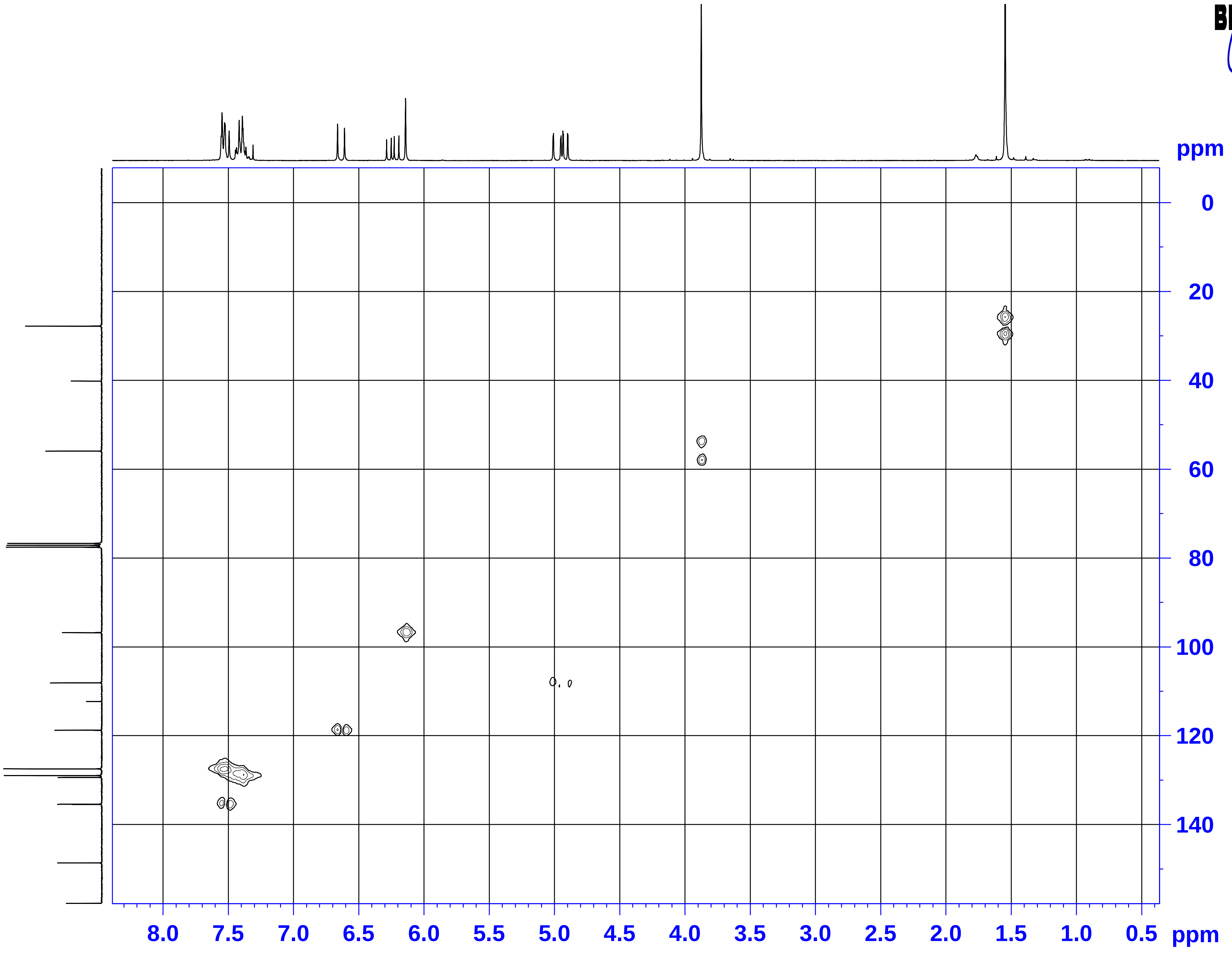

IC4
HMQCGP CDCl3 {

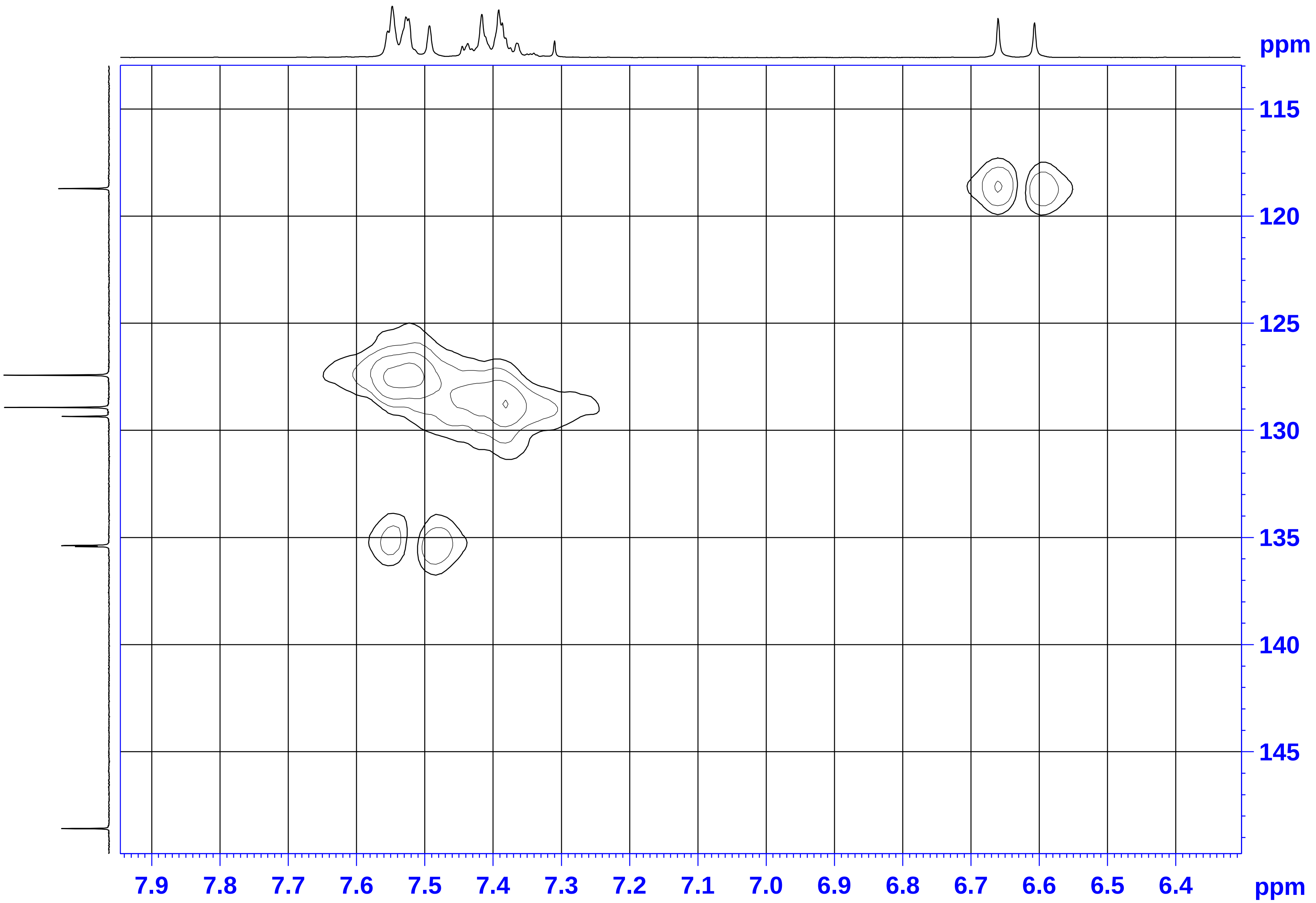

IC4
HMQCGP CDCl3 {

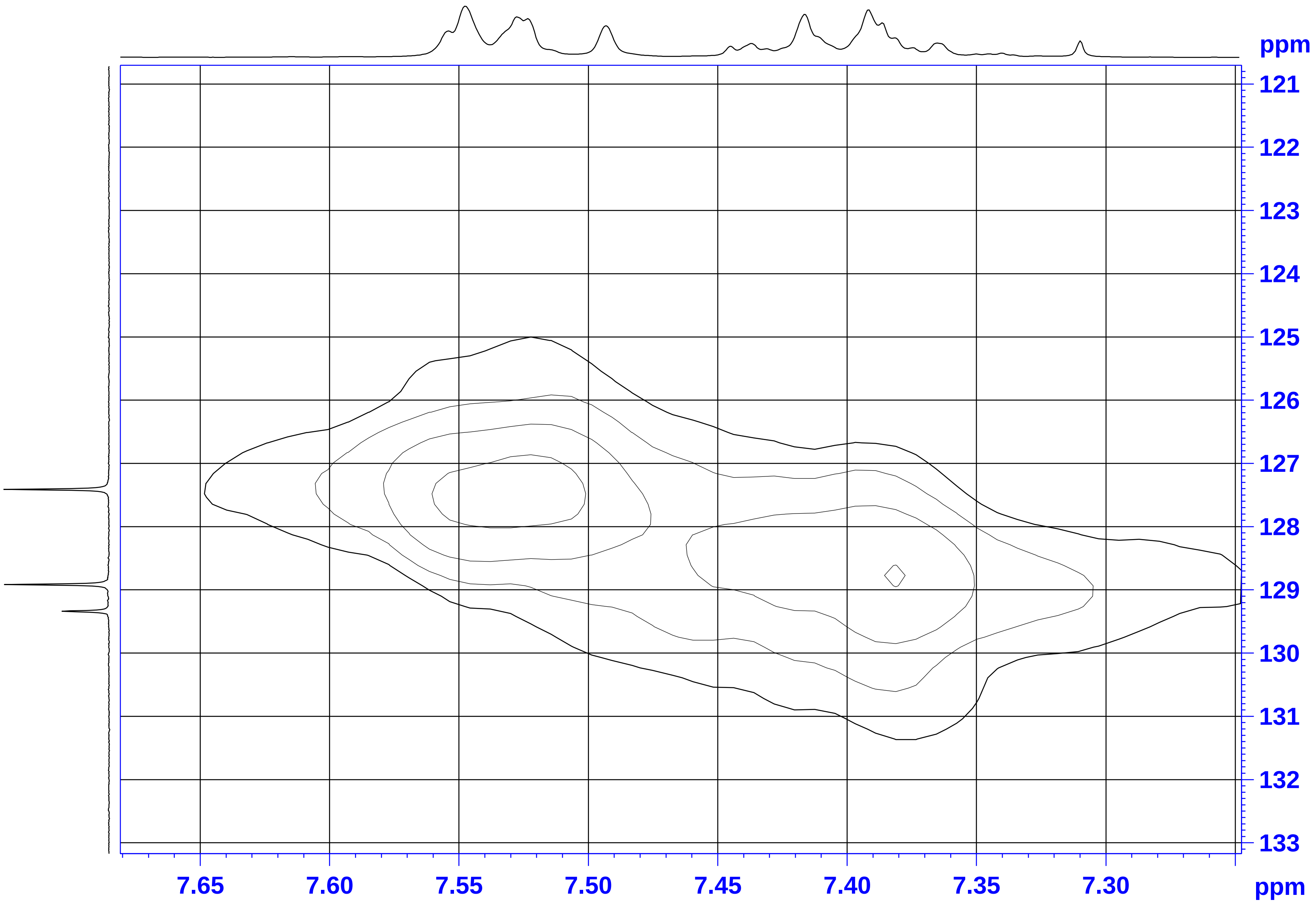

IC4
HMQCGP CDCl3 {

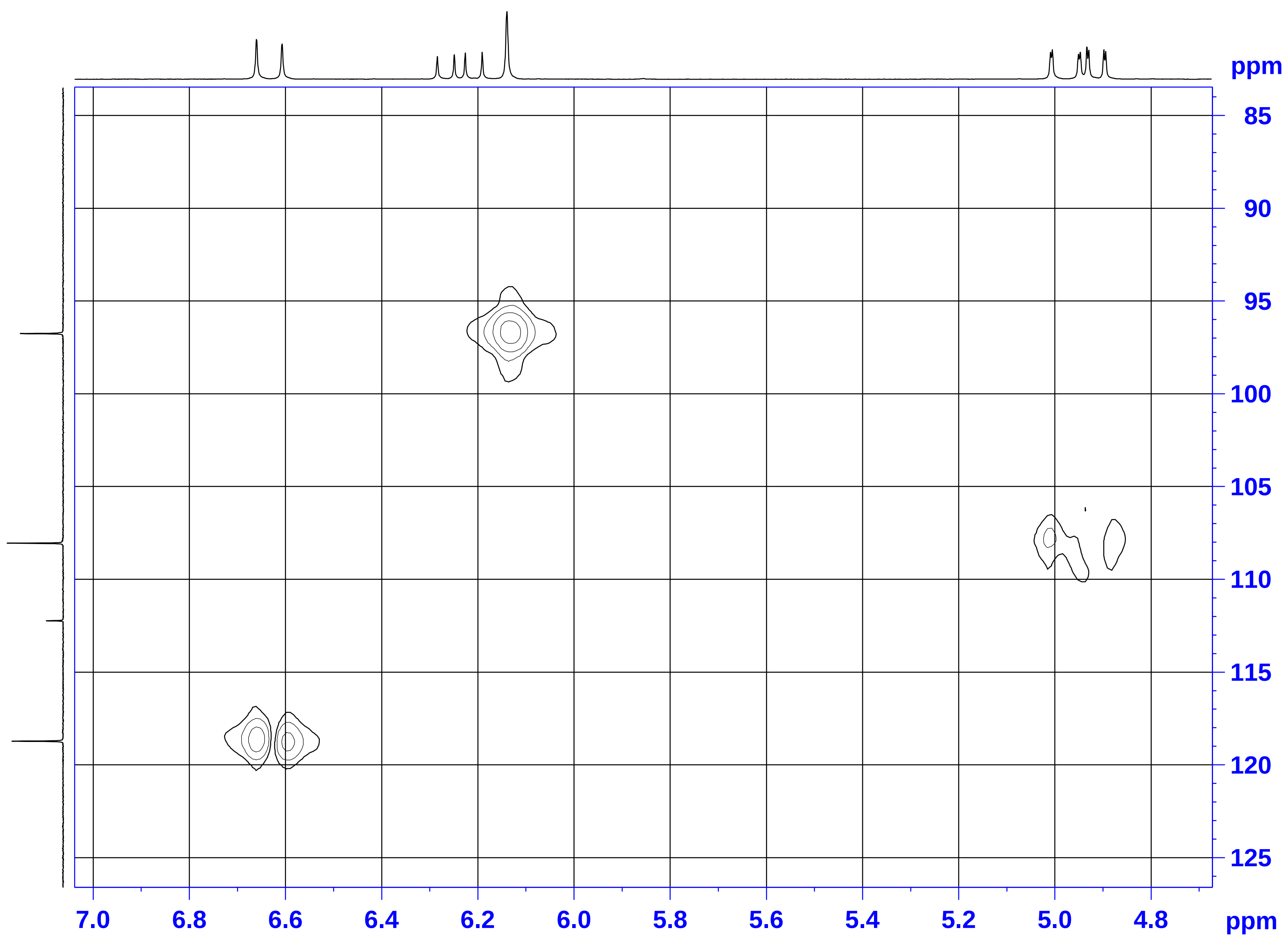

IC4
HMQCGP CDCl3 {

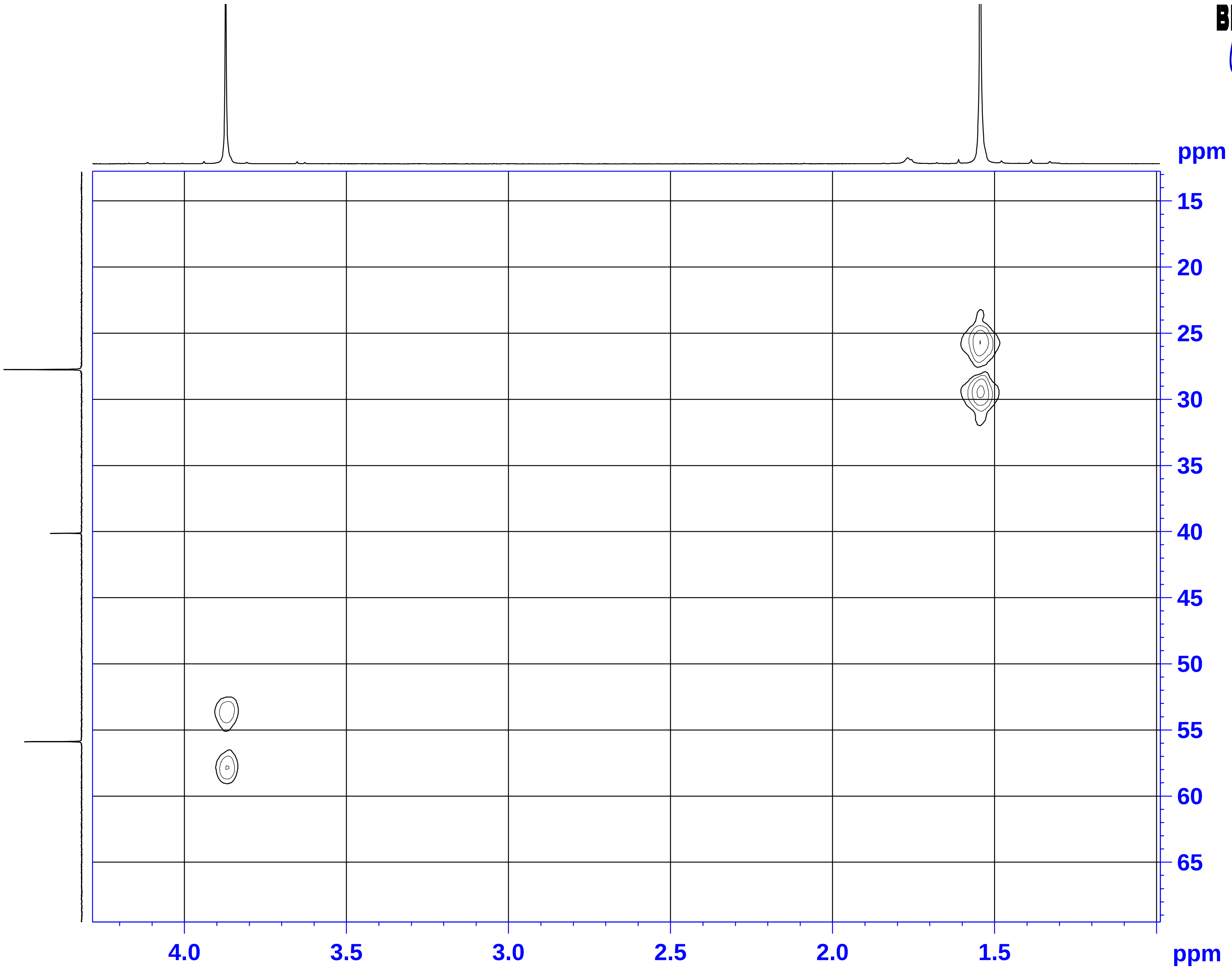

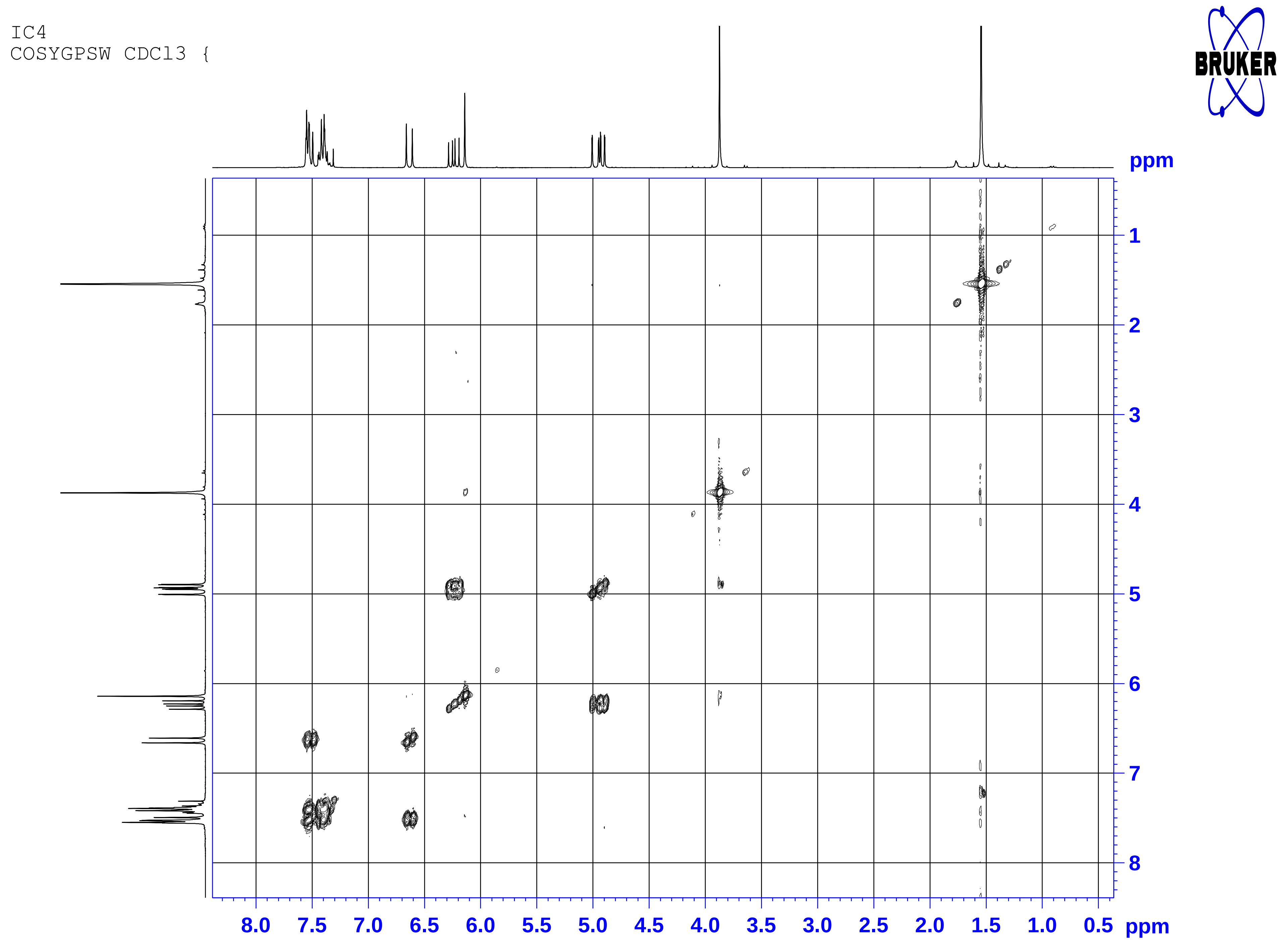

IC4
COSYGPSW CDCl3 {
BRUKER
ppm
1
2
3
4
5
6
7
8
8.0
7.5
7.0
6.5
6.0
5.5
5.0
4.5
4.0
3.5
3.0
2.5
2.0
1.5
1.0
0.5
ppm

IC4
COSYGPSW CDCl3 {

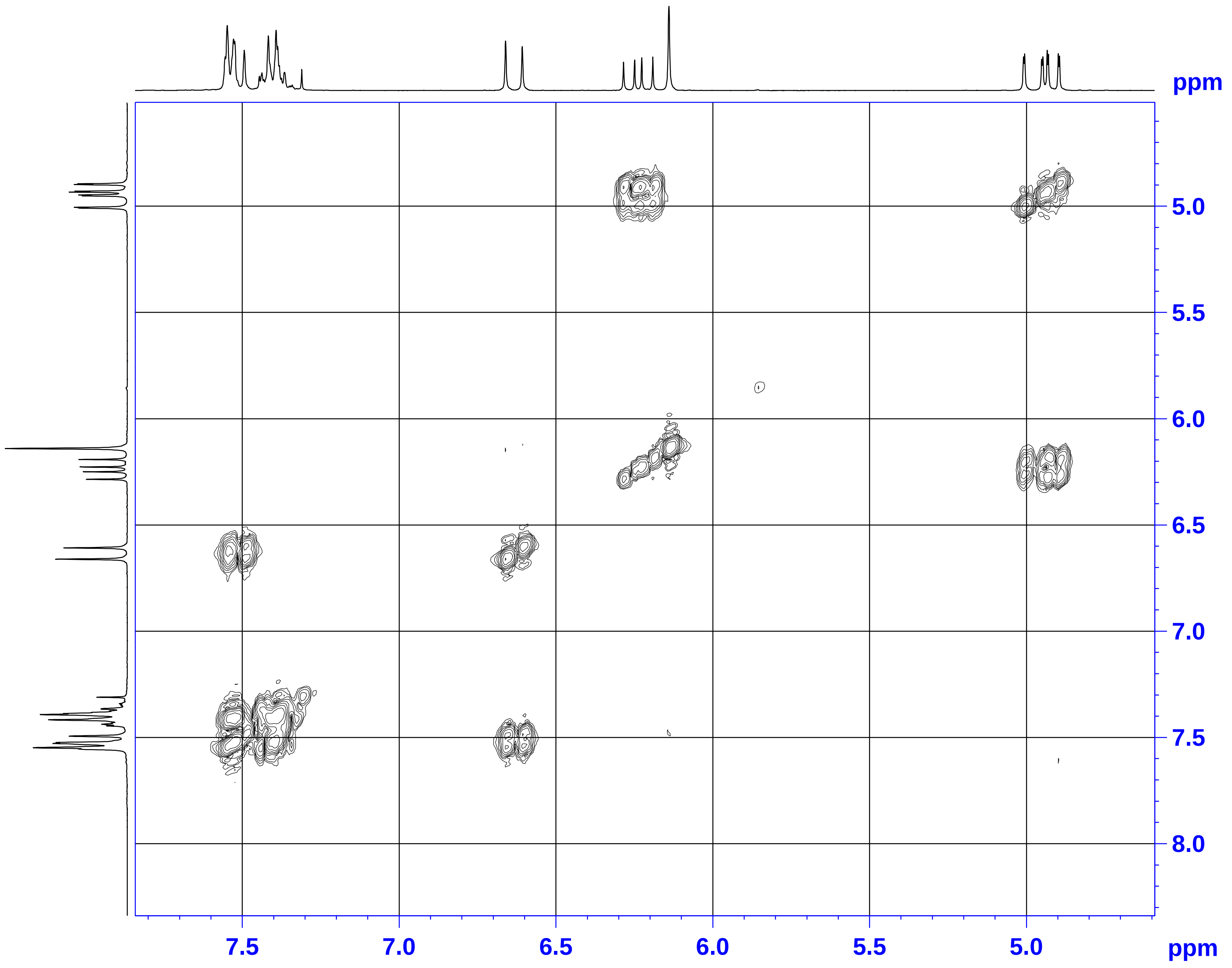

IC4
COSYGPSW CDCl3 {

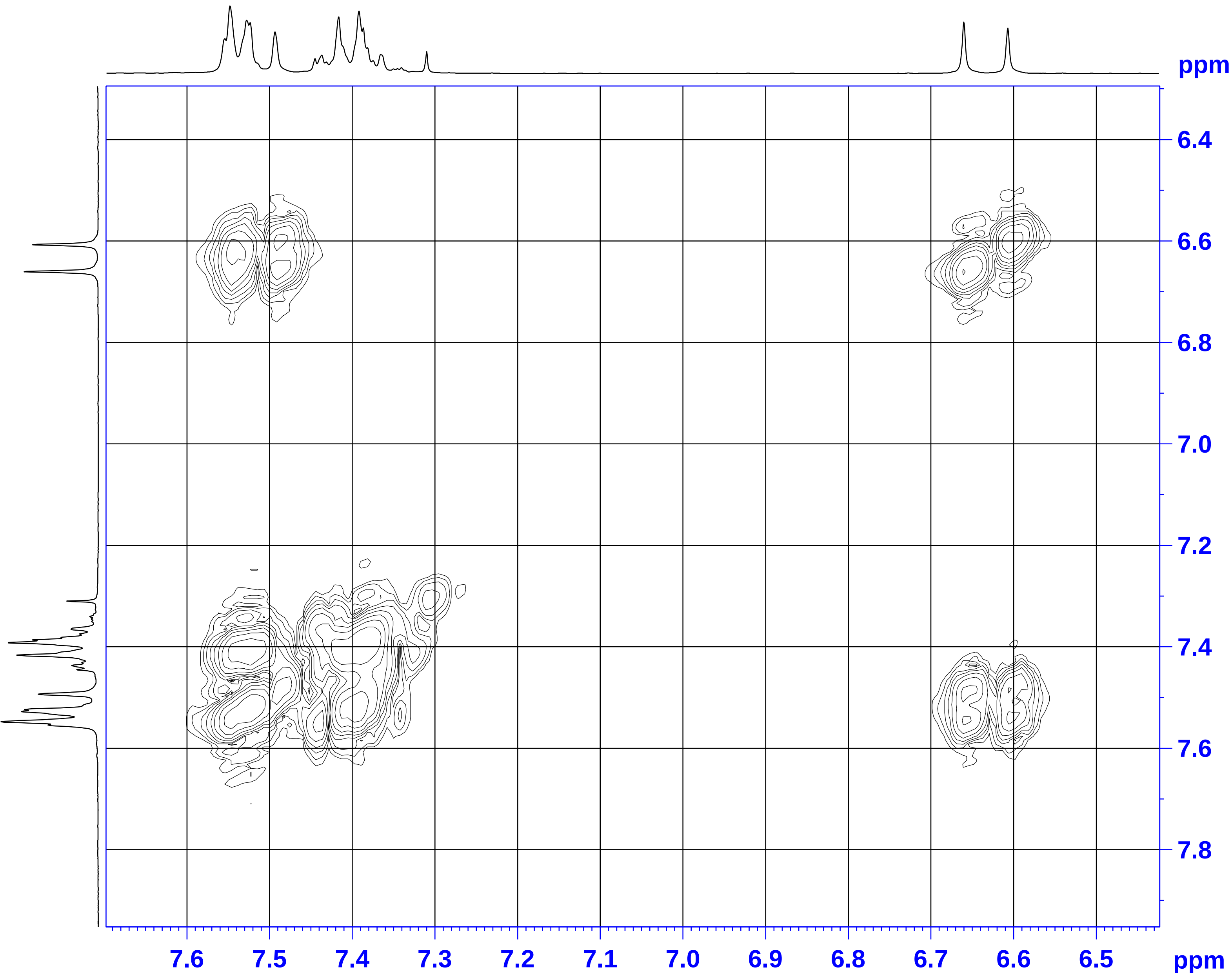

IC4
COSYGPSW CDCl3 {

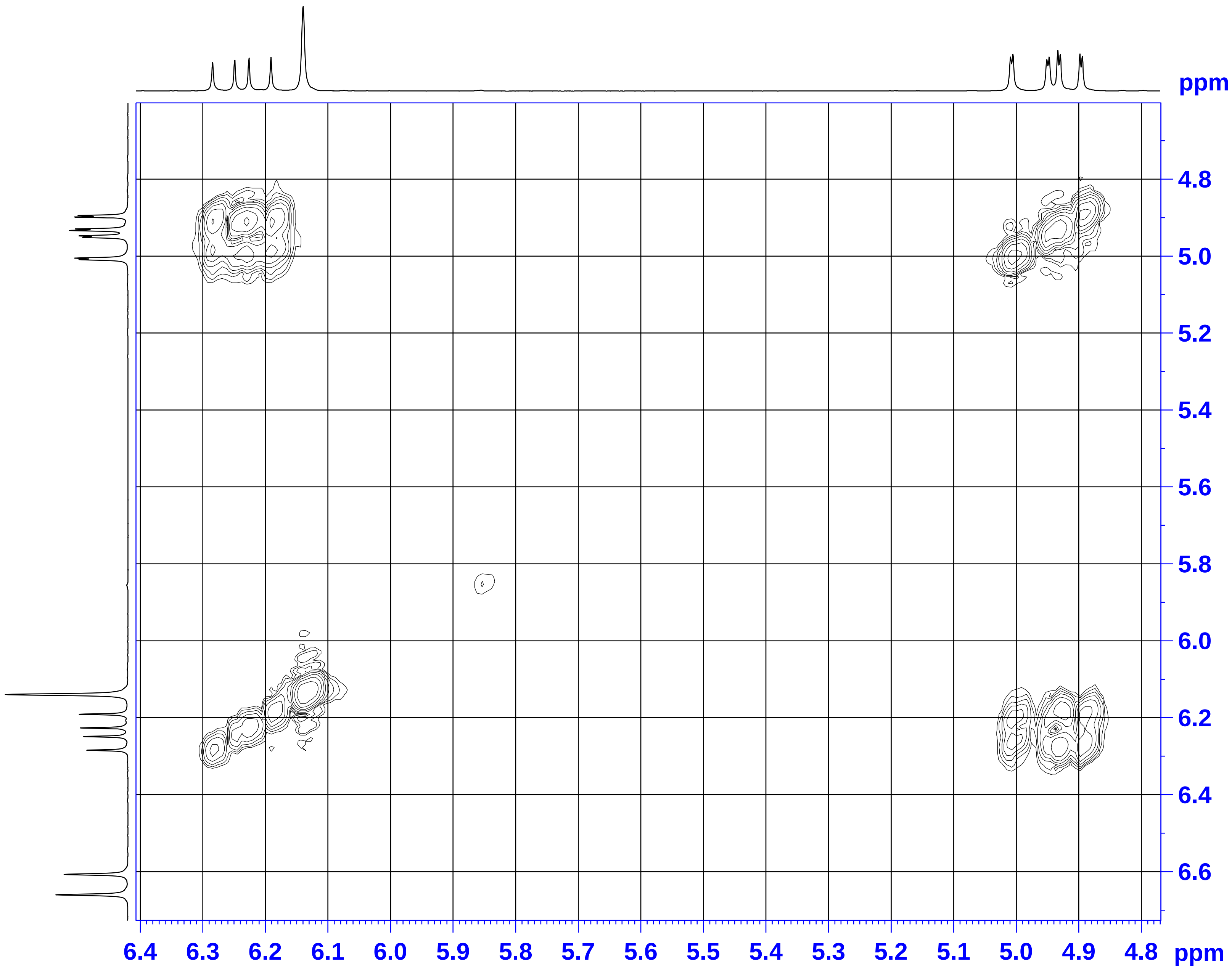

IC4
HMQCGP CDCl3

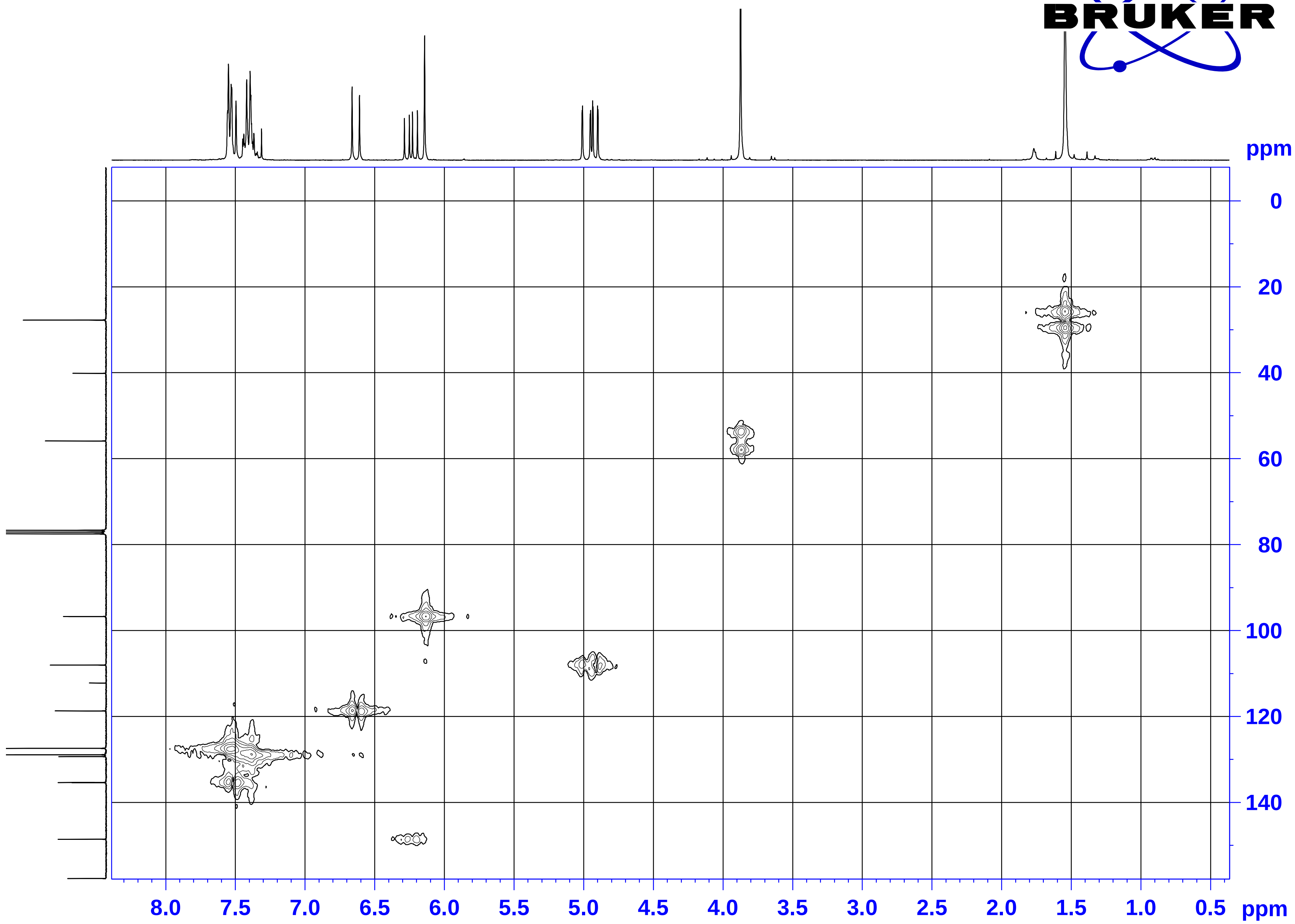

IC4
HMQCGP CDCl3

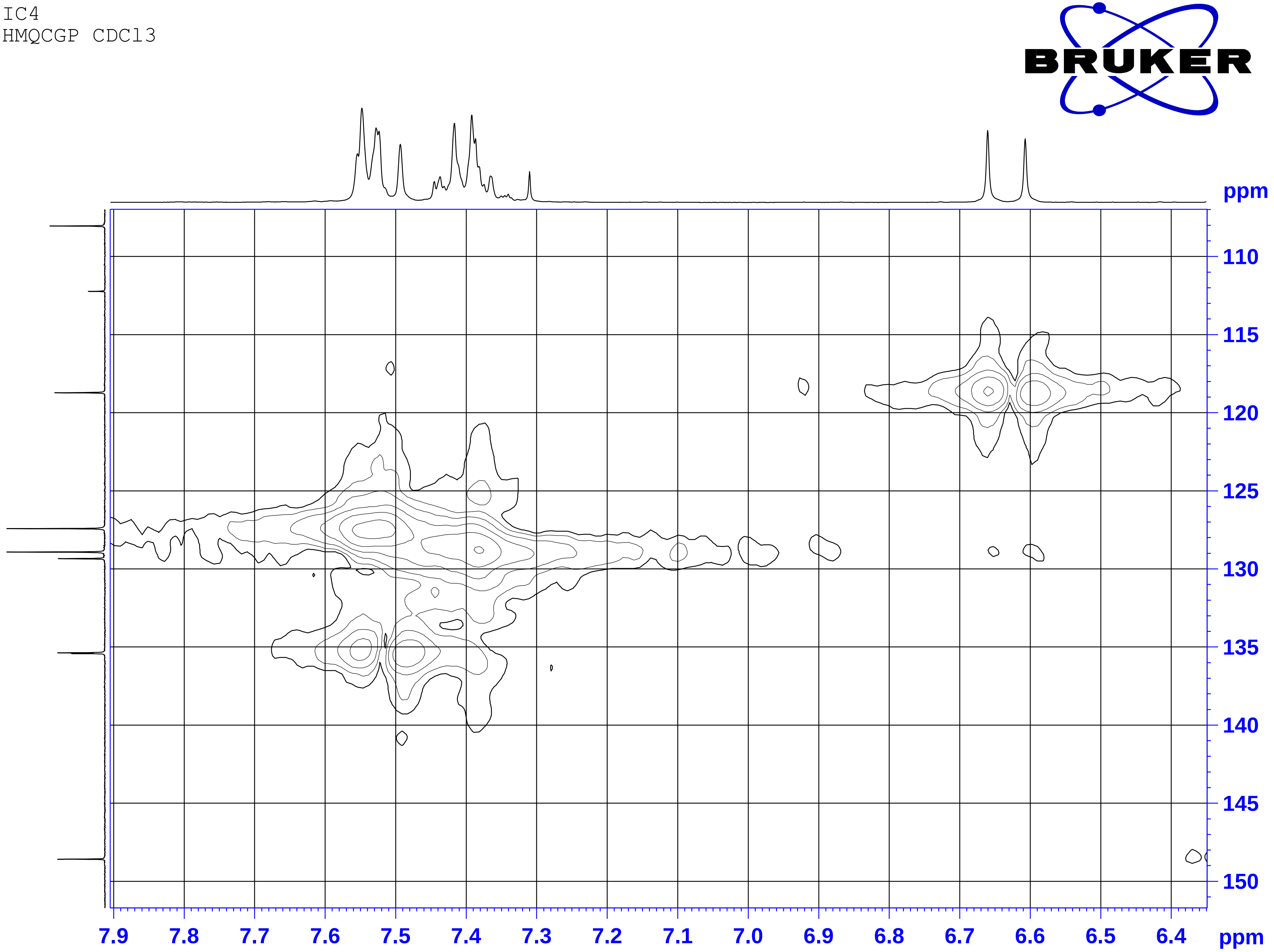

IC4
HMQCGP CDCl3

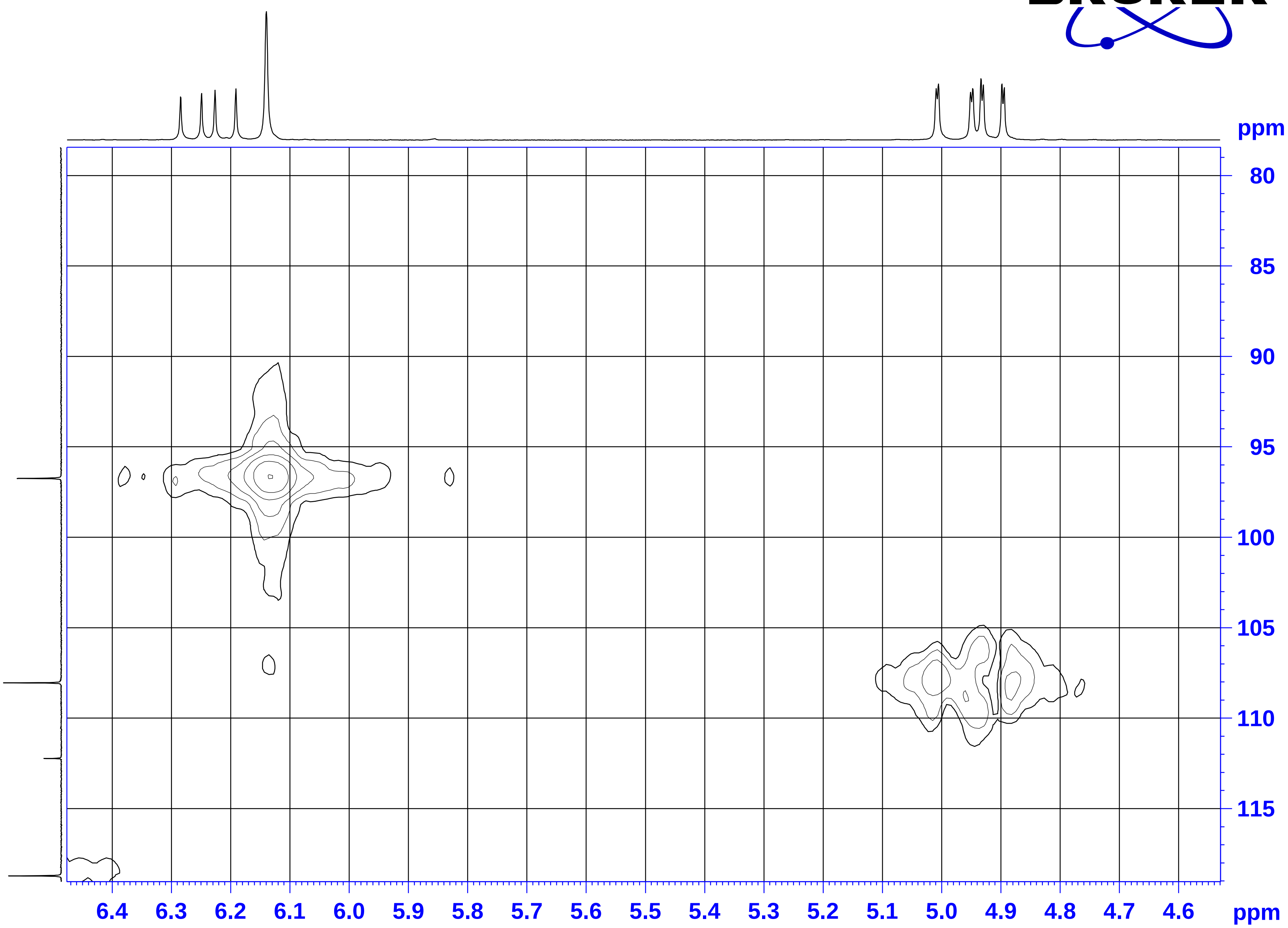

IC4
HMQCGP CDCl3

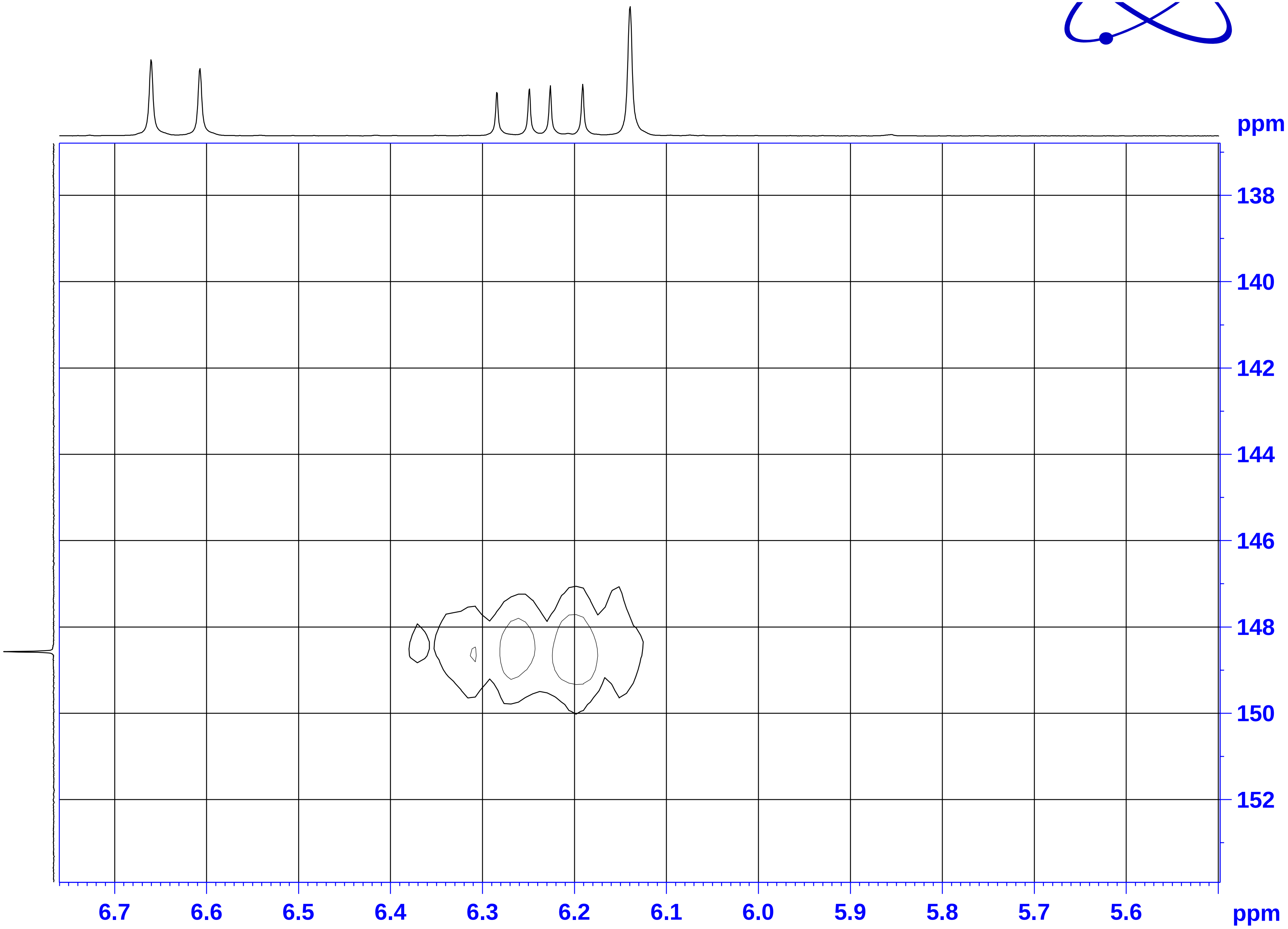

IC4
HMQCGP CDCl3

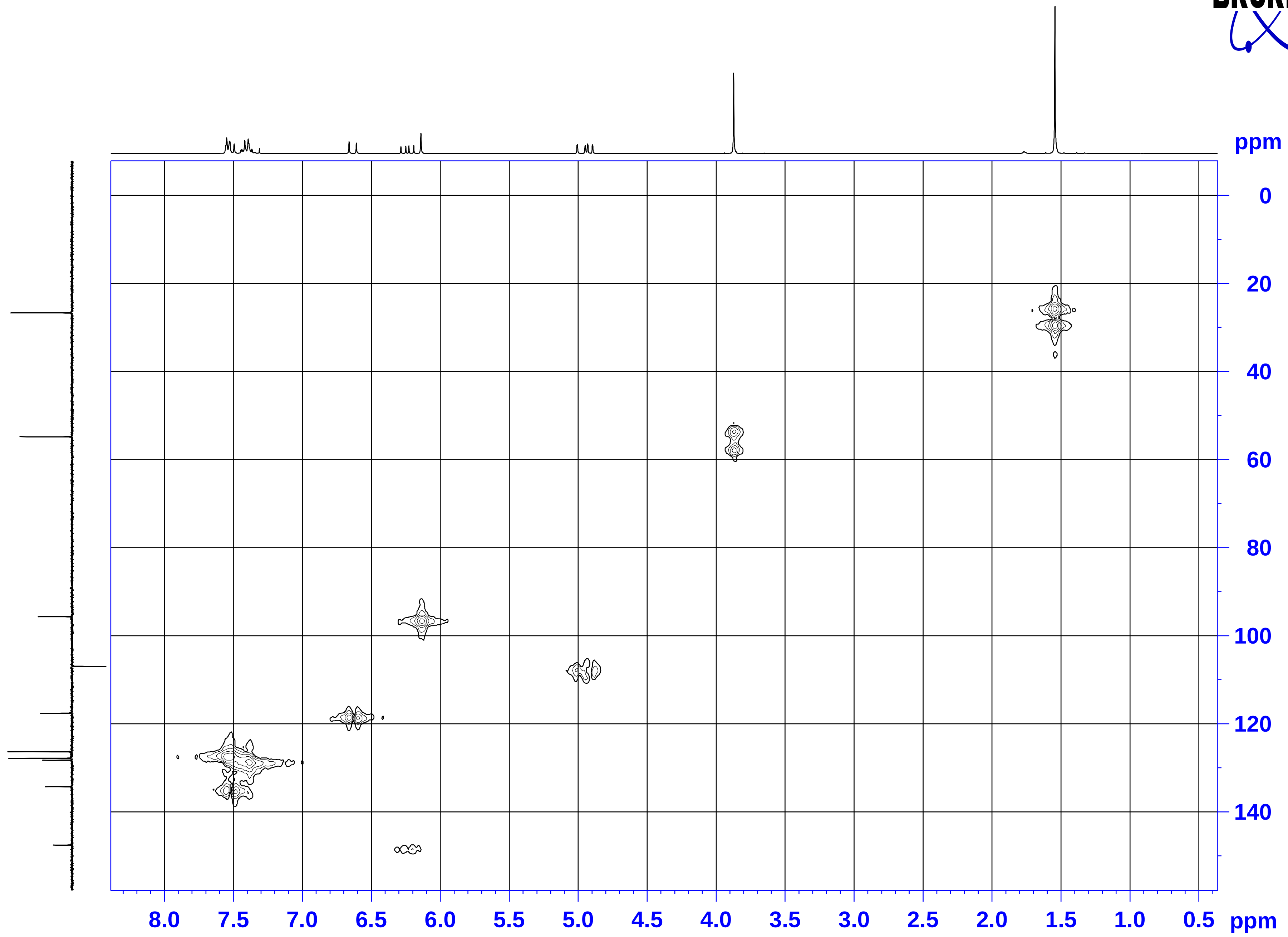

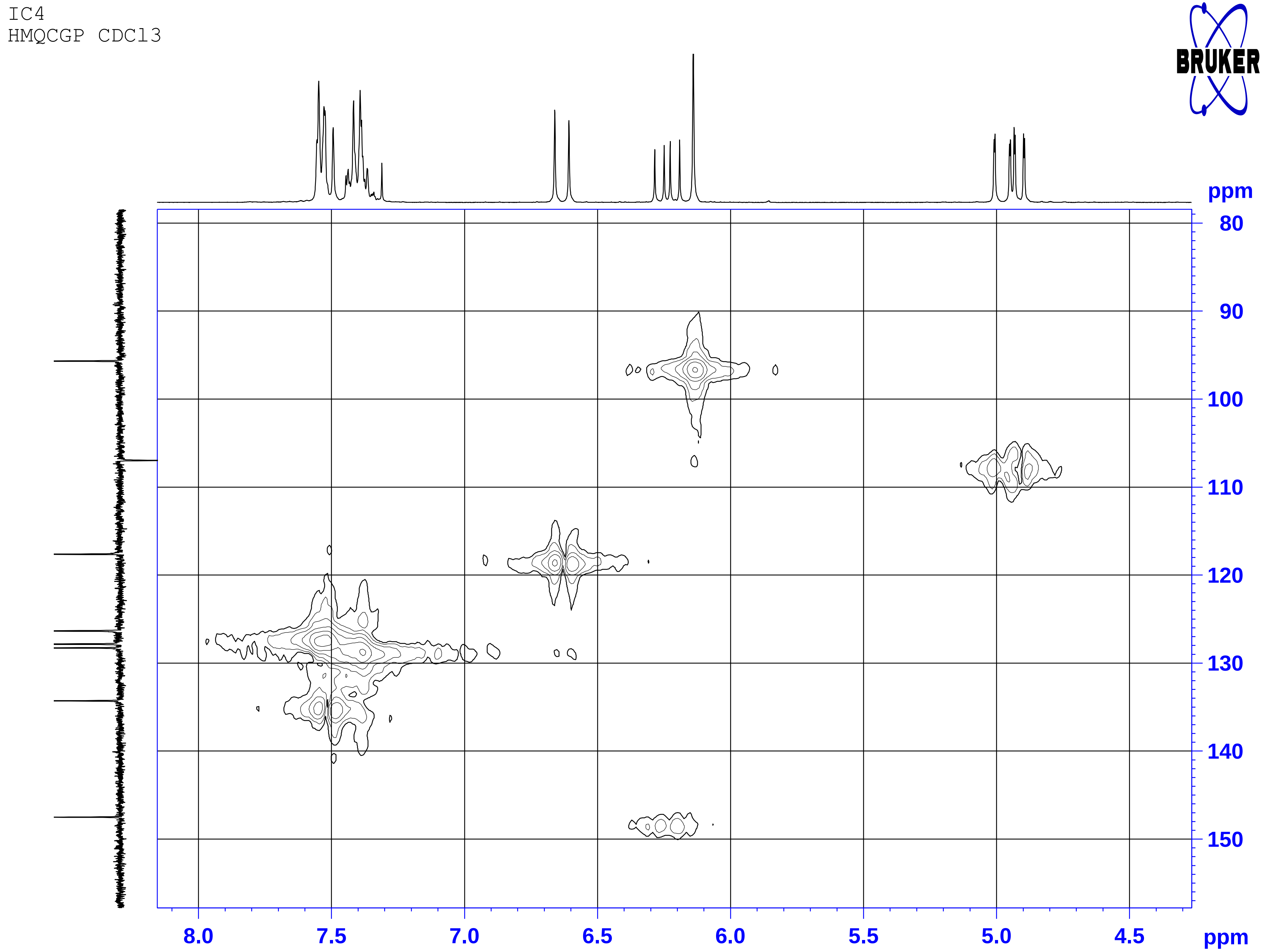
IC4
HMQCGP CDCl3
BRUKER
ppm
80
90
100
110
120
130
140
150
8.0
7.5
7.0
6.5
6.0
5.5
5.0
4.5
ppm

IC4
HMQCGP CDCl3

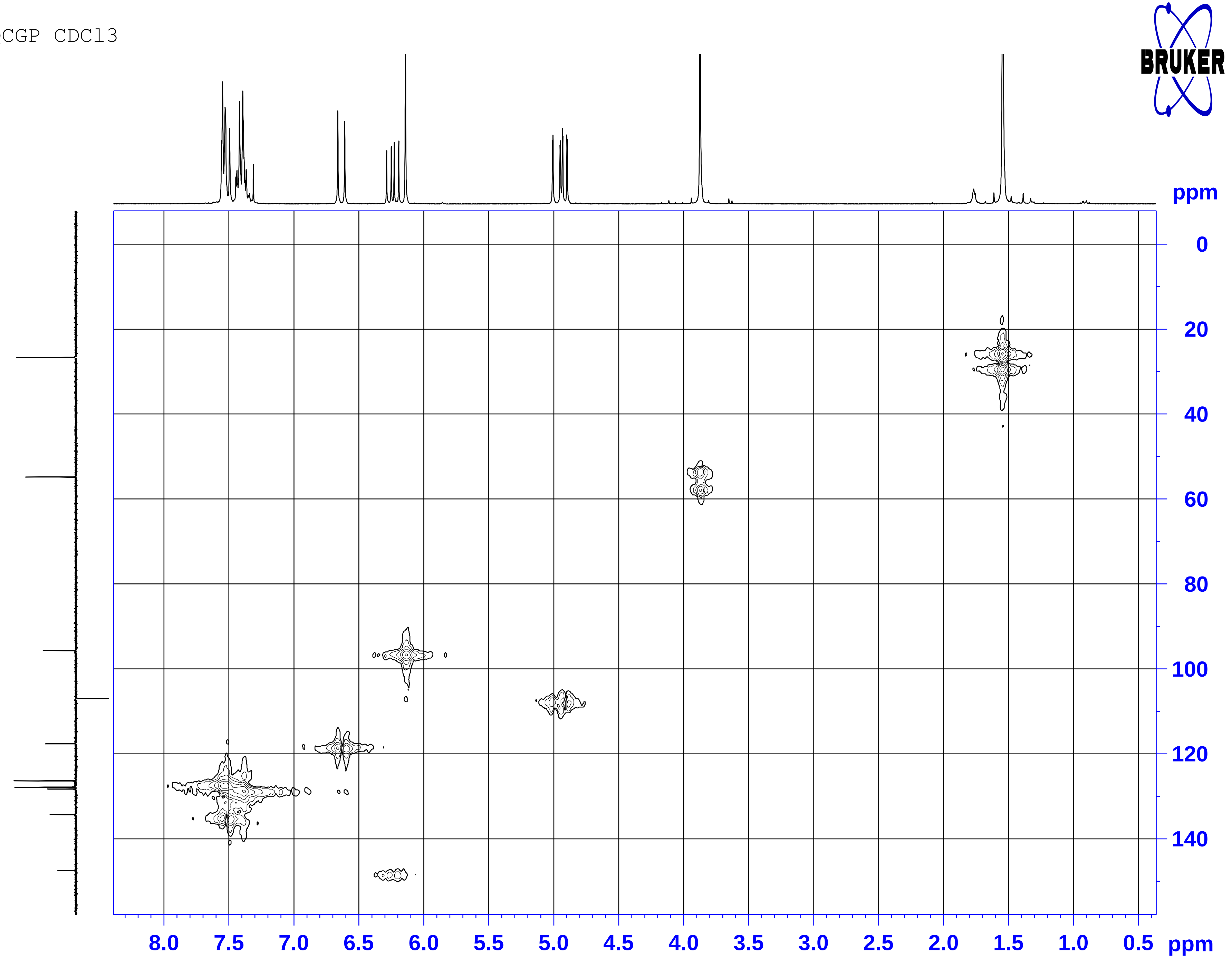

IC4
HMQCGP CDCl3

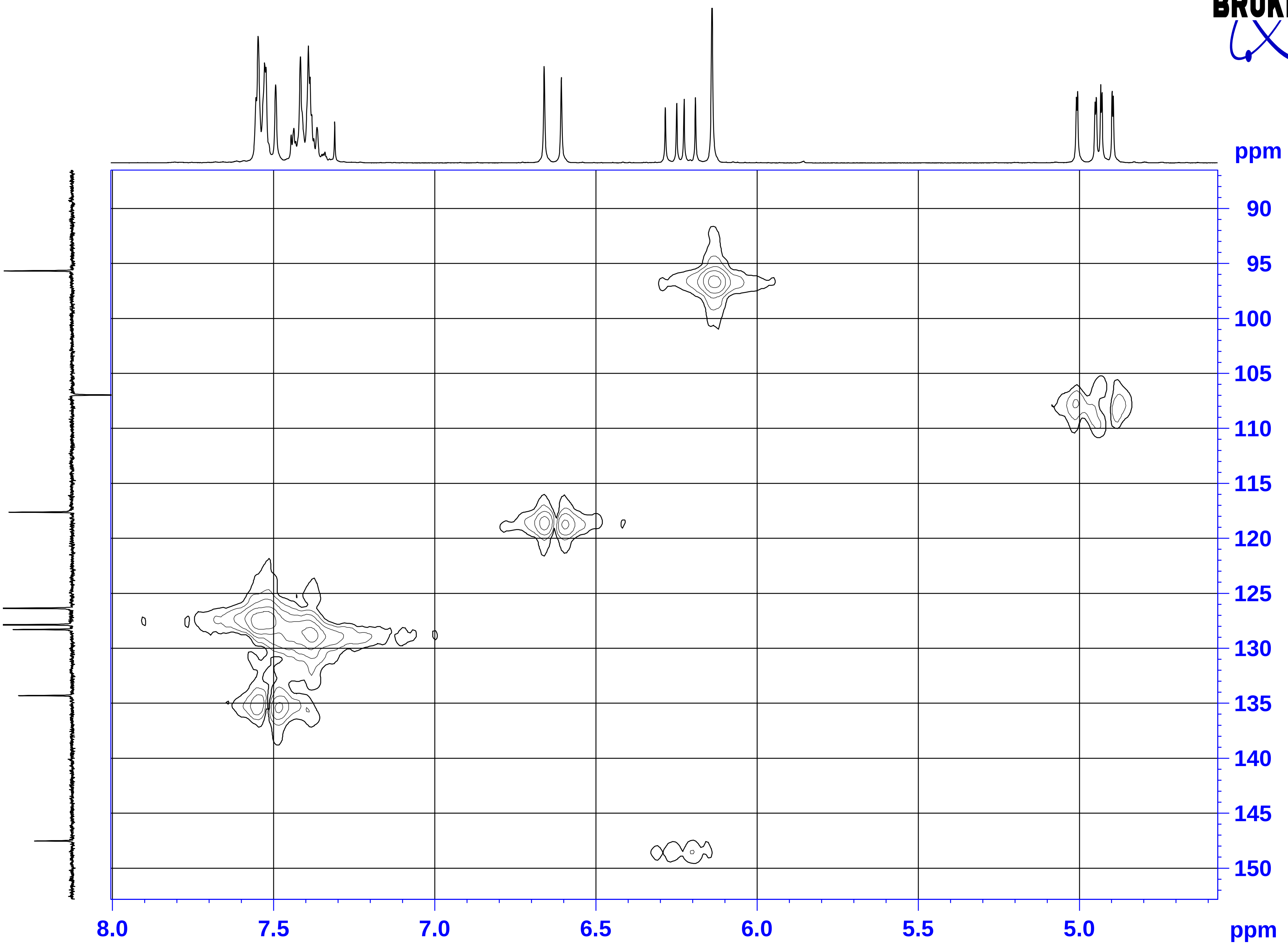

IC4
HMQCGP CDCl3

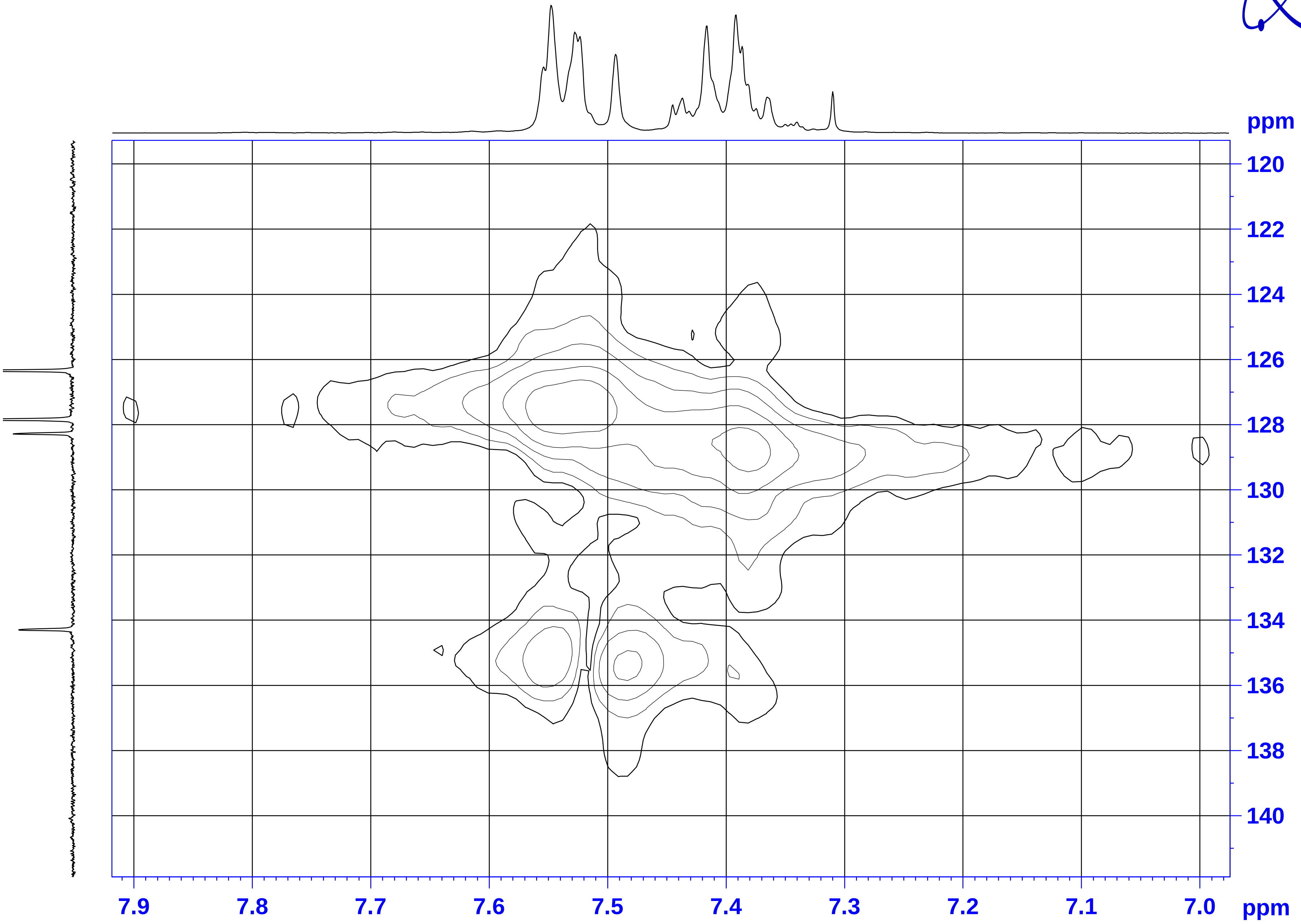

IC4
HMBCGPND CDCl3 {

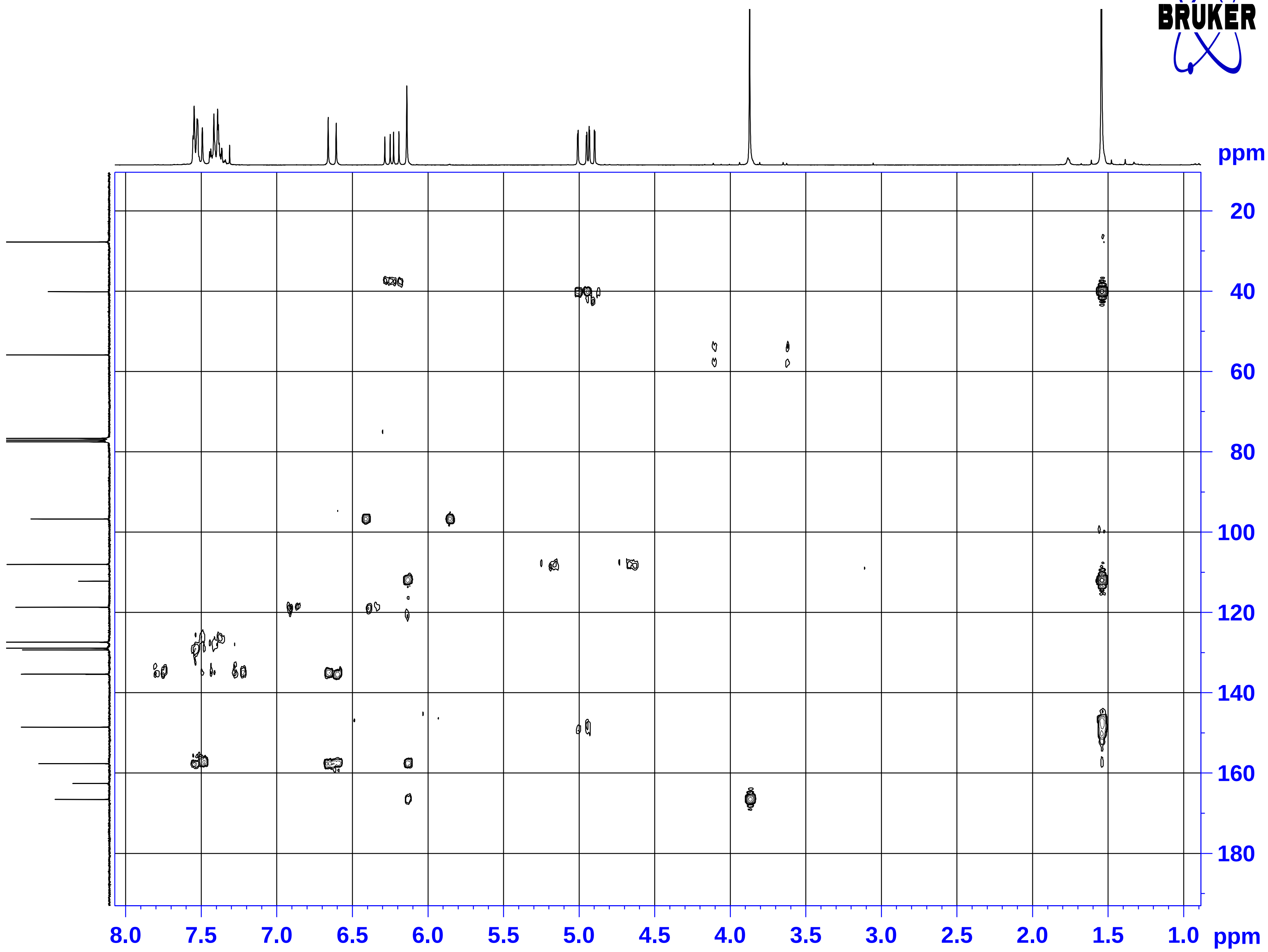

IC4
HMBCGPND CDCl3 {

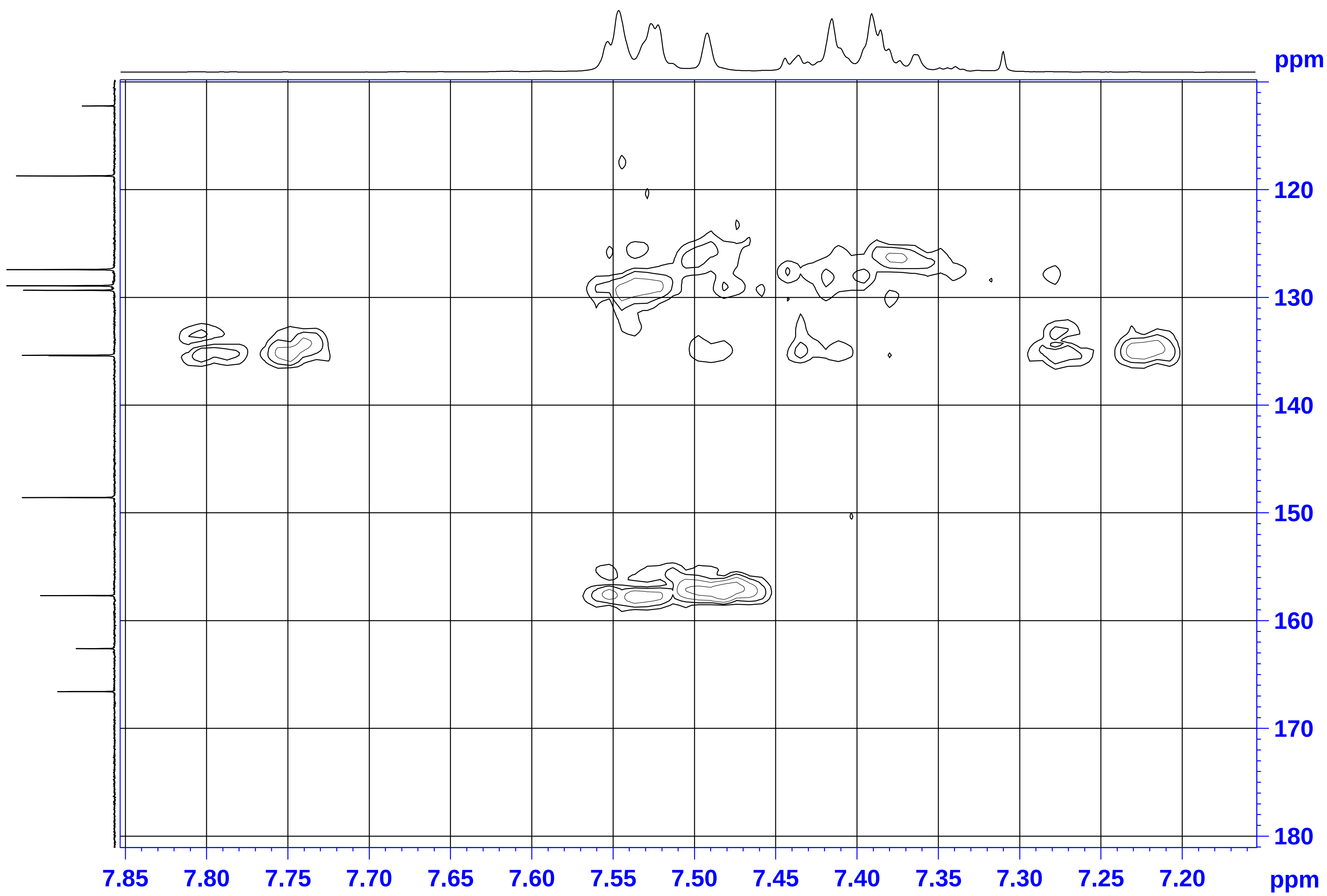

IC4
HMBCGPND CDCl3 {

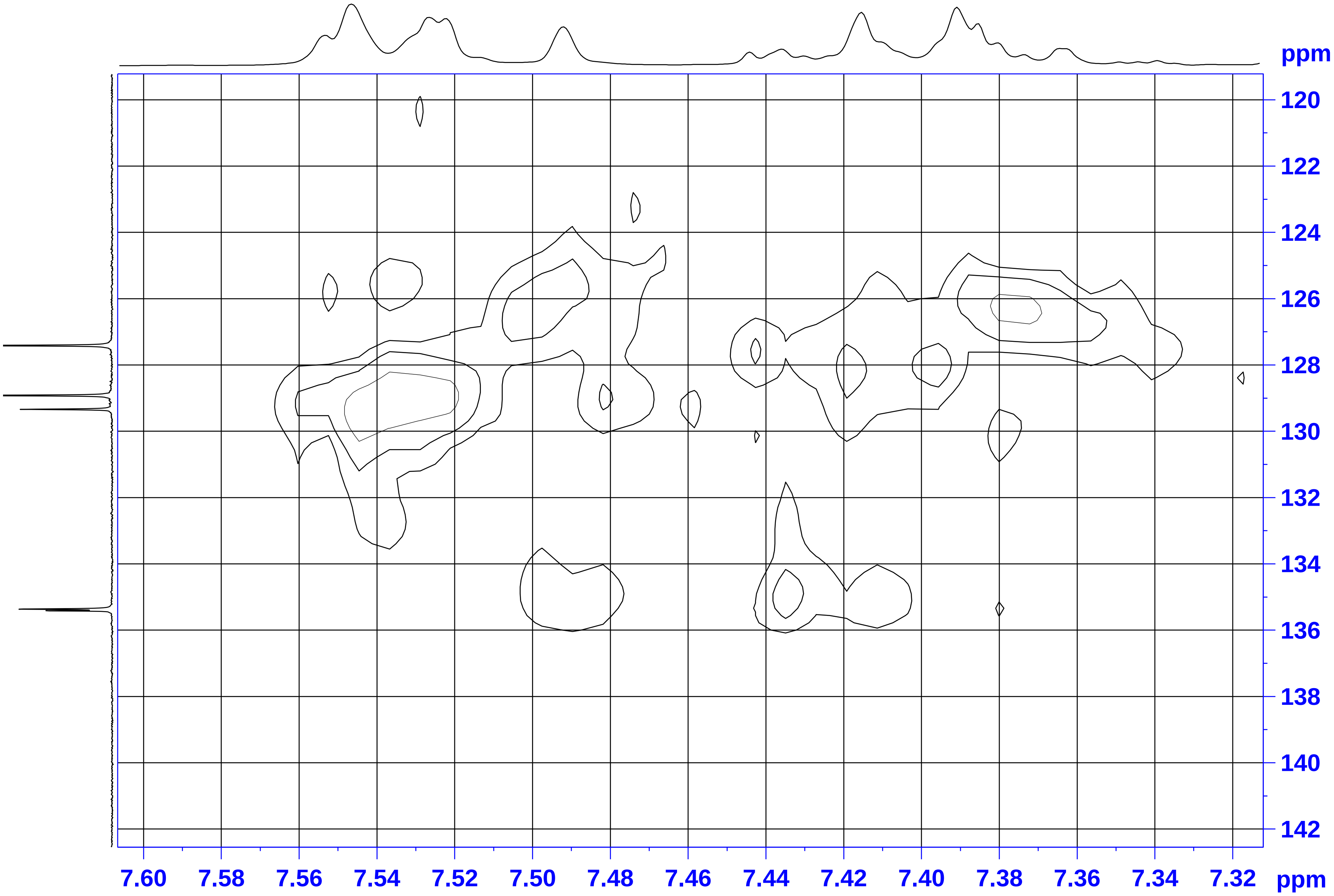

IC4
HMBCGPND CDCl3 {

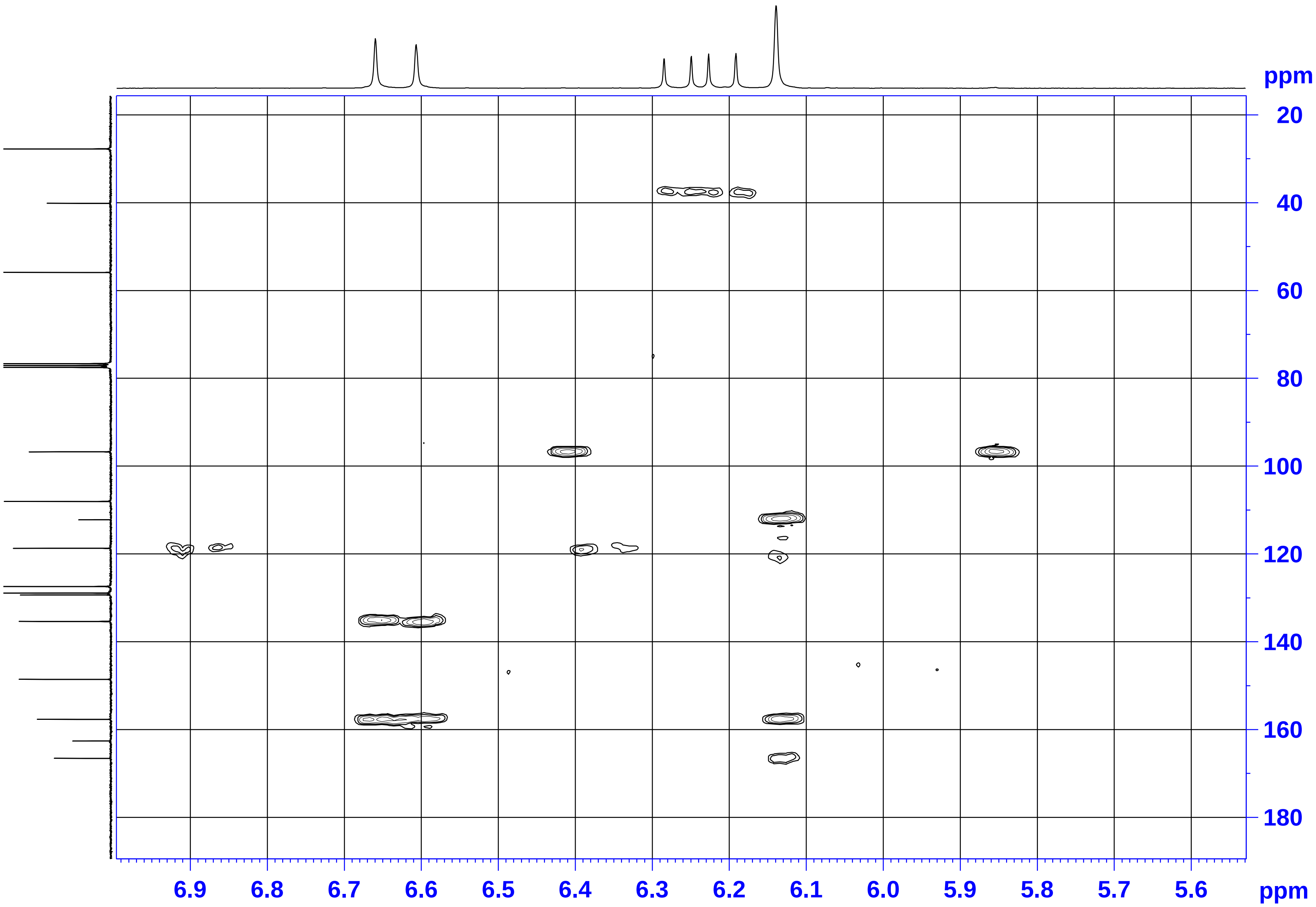

IC4
HMBCGPND CDCl3 {

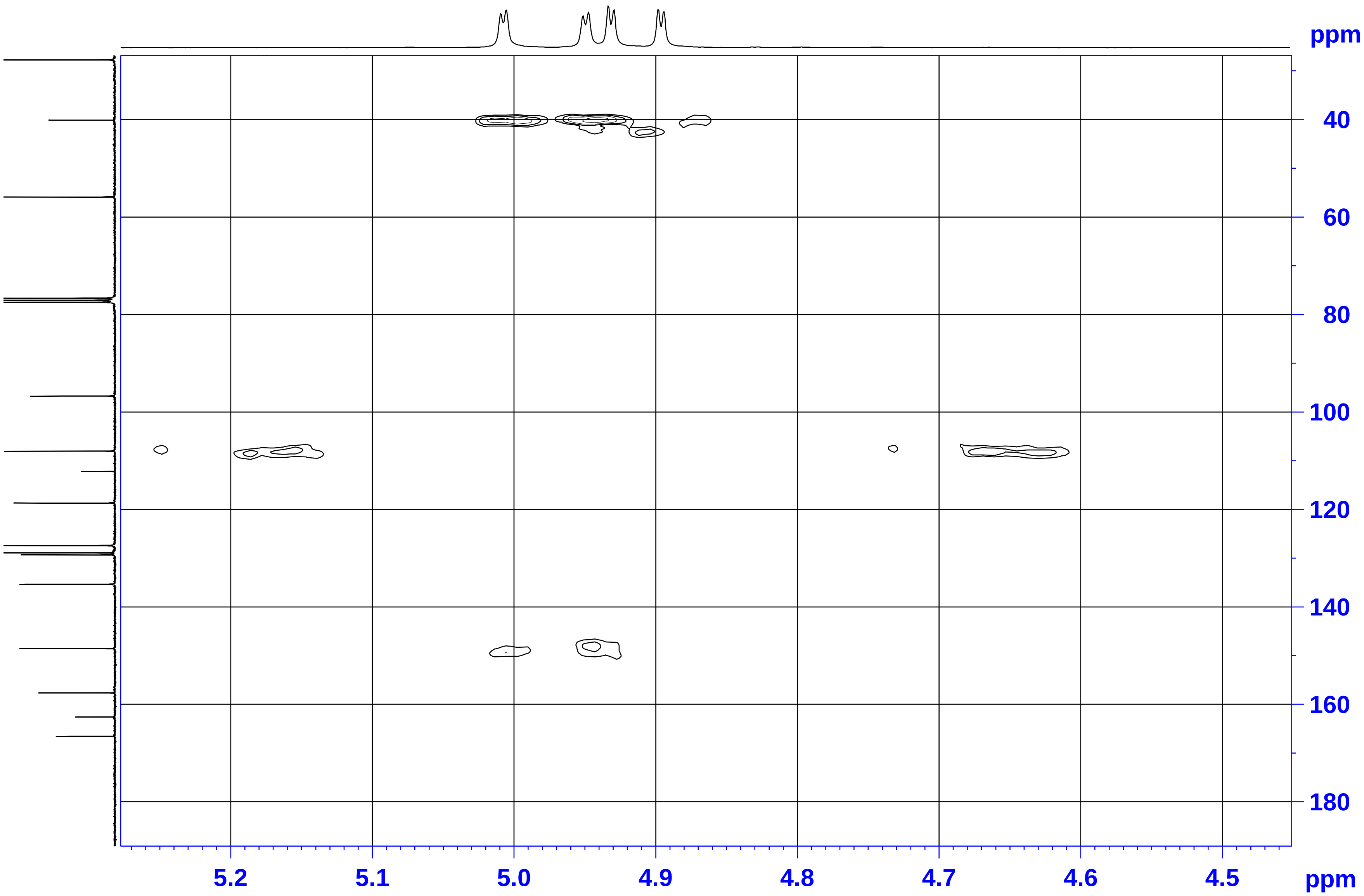

IC4
HMBCGPND CDCl3 {

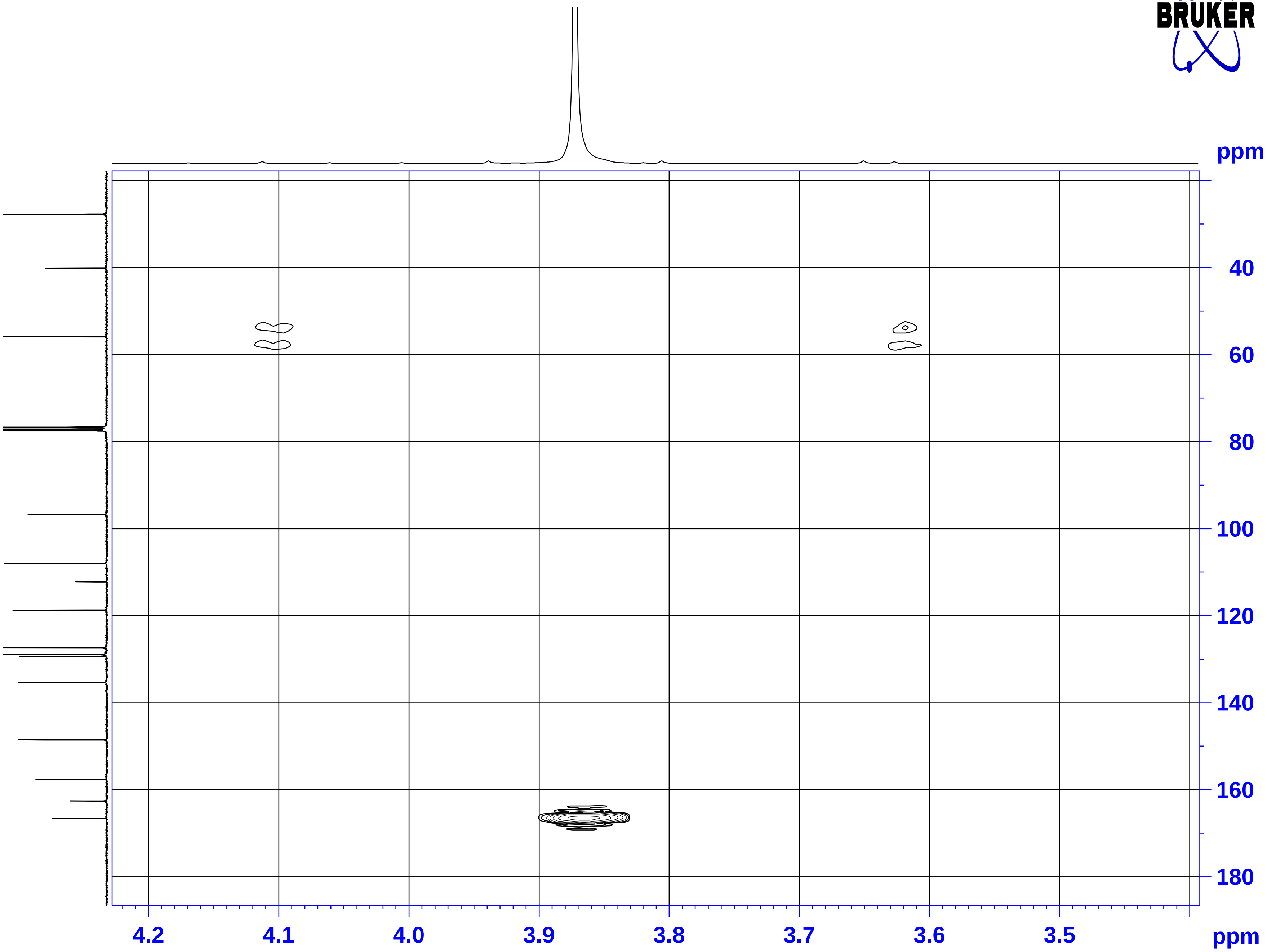

IC4
HMBCGPND CDCl3 {

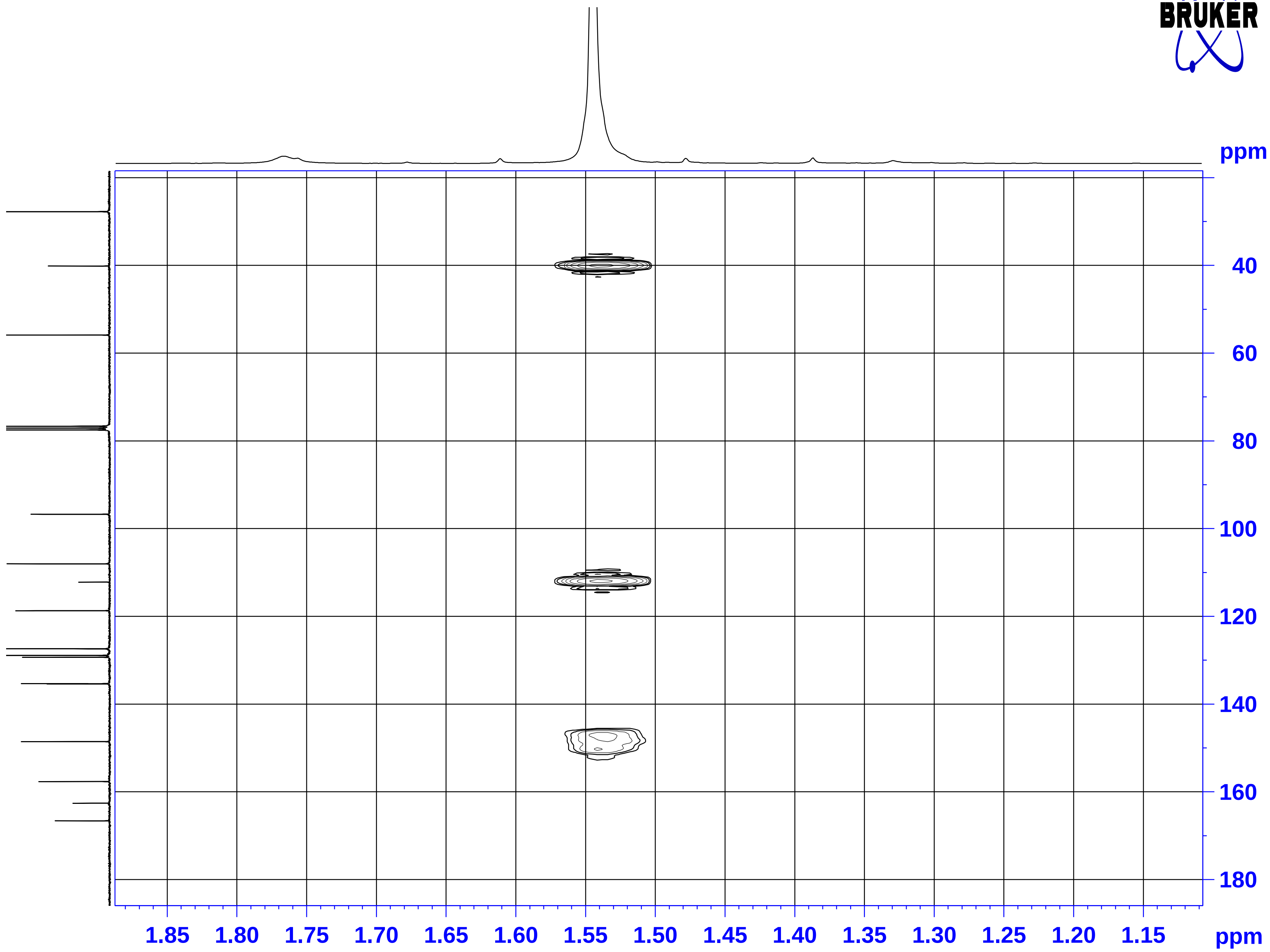

IC4
HMBCGPND CDCl3

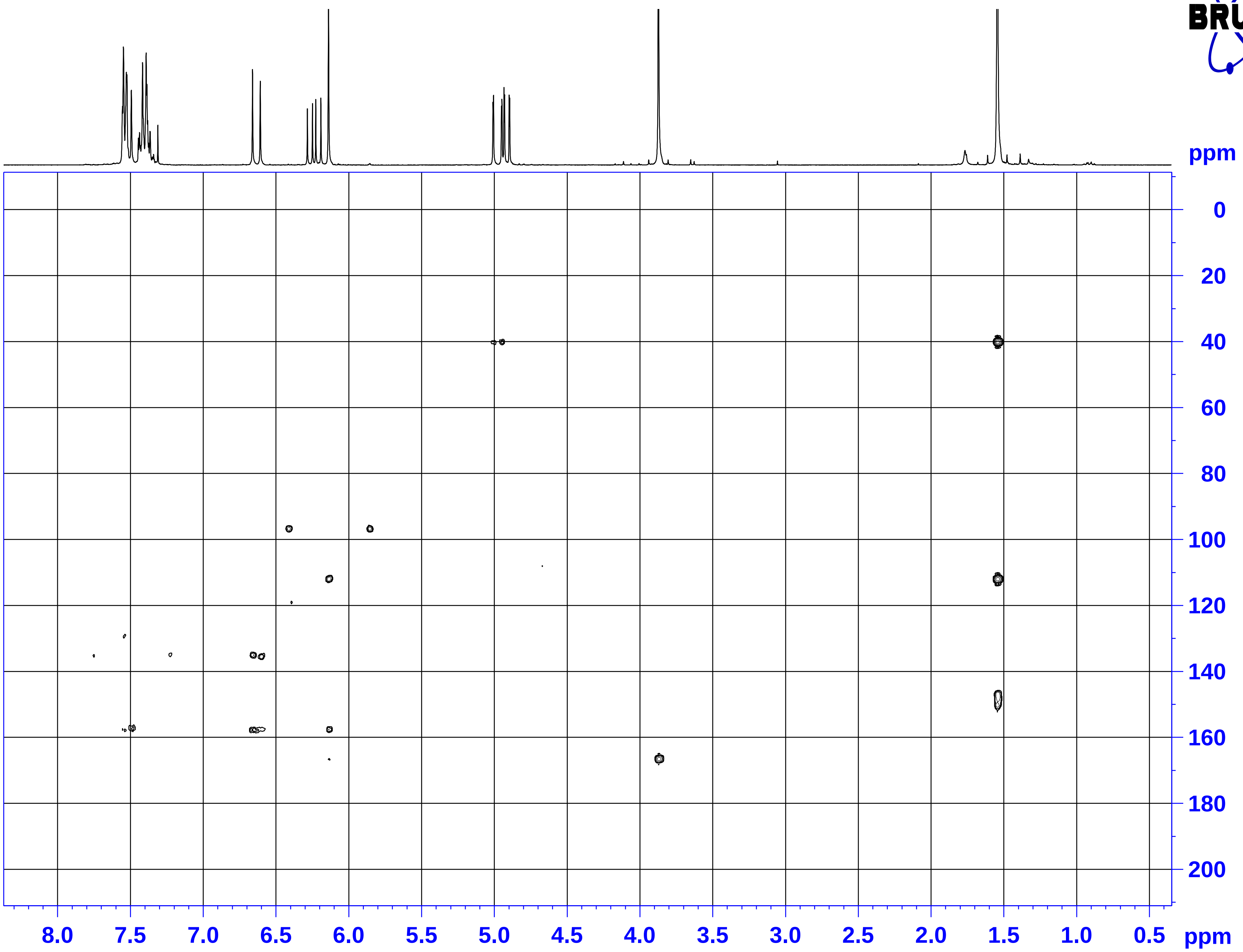

IC4
HMBCGPND CDCl3

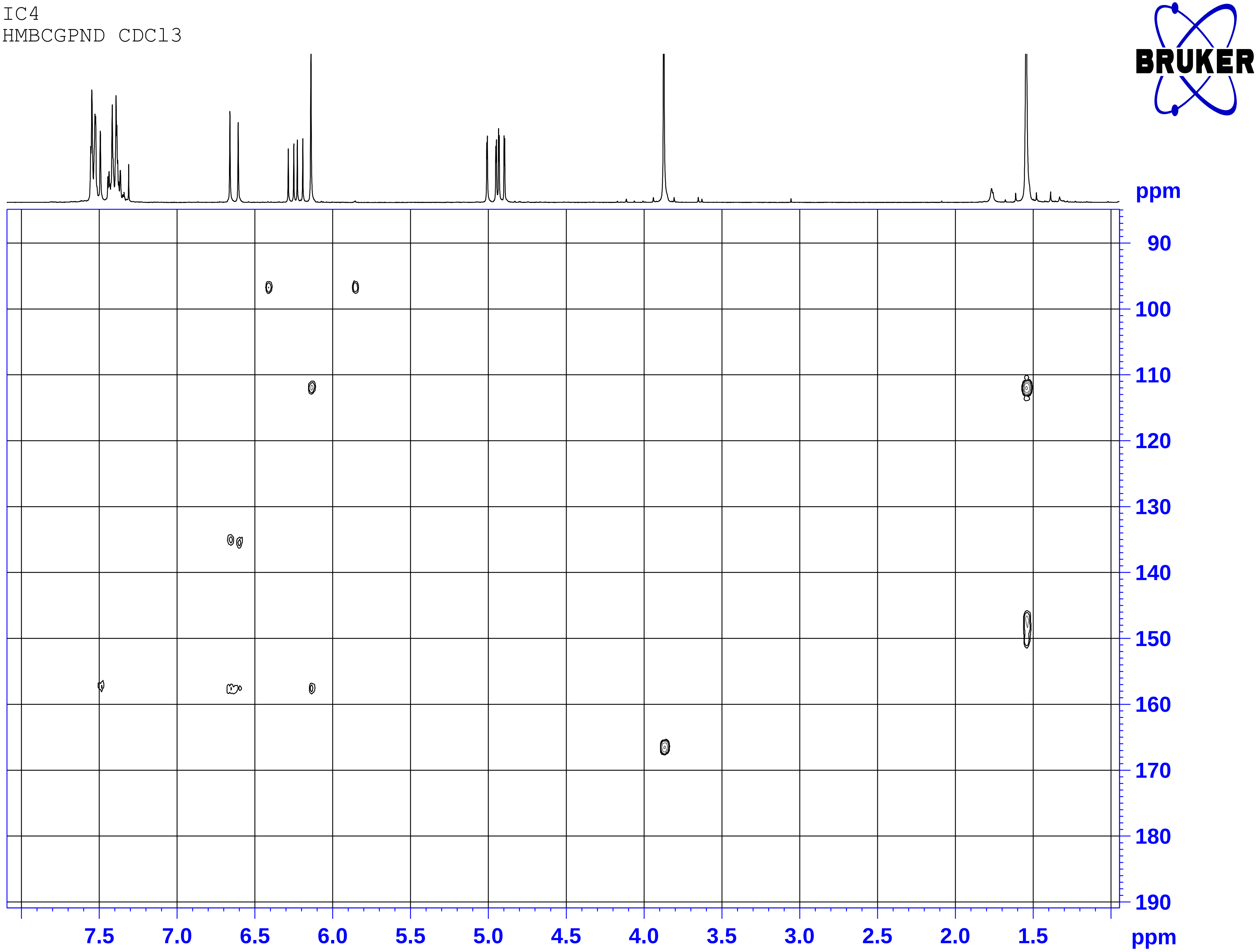

IC4
HMBCGPND CDCl3

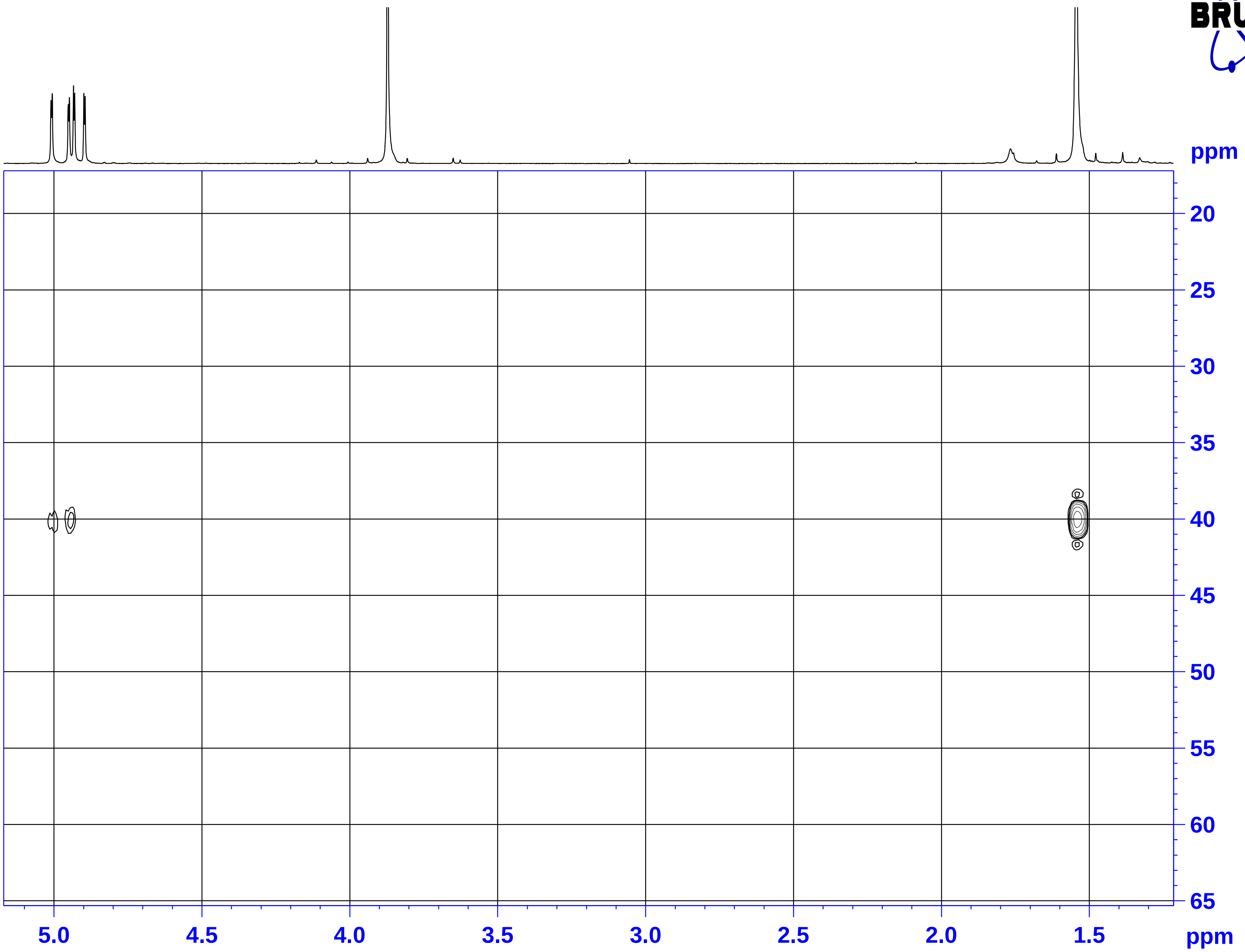

Supplement: Supplementary file 4 — 10.1186/s13065-016-0204-x 1H and 13C NMR spectra of mundulea lactone 4 as well as all the 2D NMR data justifying the revision of the NMR assignment of this compound as shown in Table 2. [file 13065_2016_204_MOESM4_ESM.pdf]
